# HUWE1 Ubiquitylates and Degrades the RAC Activator TIAM1 Promoting Cell-Cell Adhesion Disassembly, Migration, and Invasion

**DOI:** 10.1016/j.celrep.2014.12.012

**Published:** 2014-12-24

**Authors:** Lynsey Vaughan, Chong-Teik Tan, Anna Chapman, Daisuke Nonaka, Natalie A. Mack, Duncan Smith, Richard Booton, Adam F.L. Hurlstone, Angeliki Malliri

**Affiliations:** 1 Cell Signalling Group, Cancer Research UK Manchester Institute, The University of Manchester, Manchester M20 4BX, UK; 2 Faculty of Life Sciences, The University of Manchester, Oxford Road, Manchester M13 9PT, UK; 3 Department of Histopathology, The Christie Hospital and Institute of Cancer Sciences, The University of Manchester, Manchester M20 4BX, UK; 4 Biological Mass Spectrometry, Cancer Research UK Manchester Institute, The University of Manchester, Manchester M20 4BX, UK; 5 Respiratory Research Group, Institute of Inflammation and Repair, The University of Manchester and North West Lung Centre, University Hospital of South Manchester, Manchester M23 9LT, UK

## Abstract

The E3 ubiquitin ligase HUWE1, deregulated in carcinoma, has been implicated in tumor formation. Here, we uncover a role for HUWE1 in cell migration and invasion through degrading the RAC activator TIAM1, implying an additional function in malignant progression. In MDCKII cells in response to HGF, HUWE1 catalyzes TIAM1 ubiquitylation and degradation predominantly at cell-cell adhesions, facilitating junction disassembly, migration, and invasion. Depleting HUWE1 or mutating the TIAM1 ubiquitylation site prevents TIAM1 degradation, antagonizing scattering, and invasion. Moreover, simultaneous depletion of TIAM1 restores migration and invasion in HUWE1-depleted cells. Significantly, we show that HUWE1 stimulates human lung cancer cell invasion through regulating TIAM1 stability. Finally, we demonstrate that HUWE1 and TIAM1 protein levels are inversely correlated in human lung carcinomas. Thus, we elucidate a critical role for HUWE1 in regulating epithelial cell-cell adhesion and provide additional evidence that ubiquitylation contributes to spatiotemporal control of RAC.

## Highlights

• HGF-induced cell-cell adhesion disassembly, migration, and invasion require HUWE1
• HUWE1 ubiquitylates and degrades TIAM1 at cell-cell adhesions in response to HGF
• TIAM1 is the key HUWE1 target regulating cell-cell adhesion, motility, and invasion
• HUWE1 and TIAM1 expression levels are inversely correlated in lung cancer

## Graphical Abstract

**Figure:**
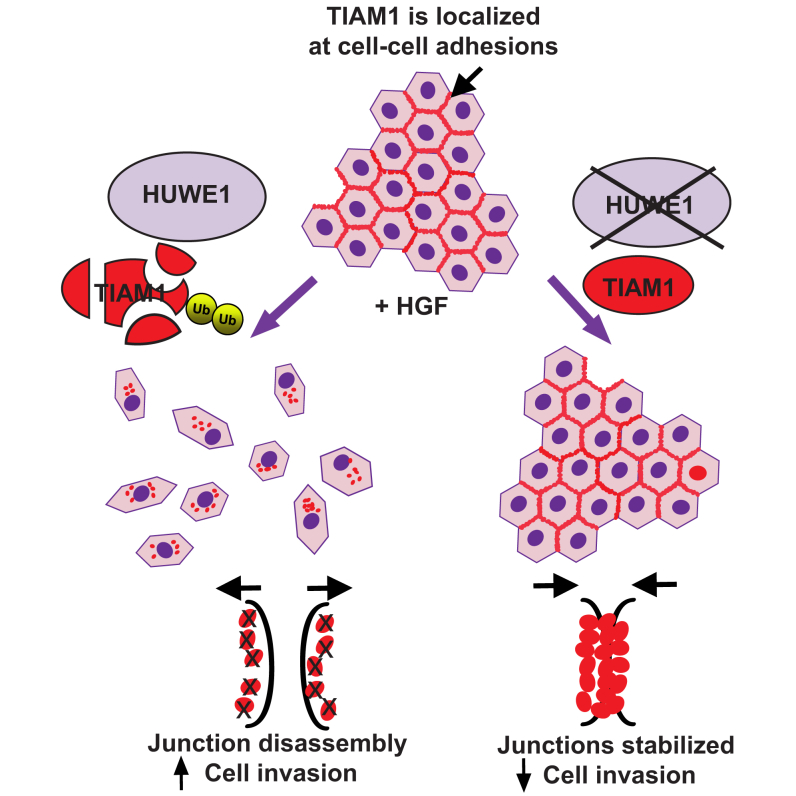

## Teaser

Malignant conversion of epithelial tumor cells is incompletely understood. Here, Vaughan et al. demonstrate that the E3 ligase HUWE1 degrades the RAC activator TIAM1 following HGF stimulation, promoting cell junction disassembly, motility, and invasion in epithelial cells including lung cancer cells. This indicates a critical role for HUWE1 in lung cancer dissemination.

## Introduction

Metastasis, a multistep process beginning with local invasion and culminating in the colonization of distant organs by cancer cells, is responsible for more than 90% of all cancer deaths (Sleeman and Steeg, 2010). Metastasis of carcinoma cells often commences with the disassembly of junctional complexes and downregulation of other epithelial traits coupled with the acquisition of a migratory and invasive mesenchymal phenotype (so-called epithelial-mesenchymal transition [EMT]). EMT is elicited by growth factors such as hepatocyte growth factor (HGF) secreted by tumor and stromal cells. Acting through its cognate receptor, c-MET, HGF induces rapid disassembly of adherens junctions through stimulating the ubiquitylation and associated proteasomal degradation of junctional proteins like E-CADHERIN (Fujita et al., 2002).

Ubiquitylation—the covalent attachment of ubiquitin to lysine residues on a target protein—is carried out by three enzymes: ubiquitin activating enzyme (E1), ubiquitin-conjugase (E2), and ubiquitin ligase (E3), each comprising a family of proteins. The HECT, UBA, and WWE domain-containing protein 1 (HUWE1) is a member of the HECT E3 ubiquitin ligase family whose substrates include key proteins such as p53 and MYC (Adhikary et al., 2005, Chen et al., 2005), which regulate diverse cellular responses including proliferation and survival with often opposing outcomes. Unsurprisingly, HUWE1 has been ascribed both putative oncoprotein and tumor suppressor functions. Adding to this controversy, HUWE1 is overexpressed in some cancers but downregulated in others (Adhikary et al., 2005, Zhao et al., 2009). Clearly, further investigation is required to resolve the contribution of HUWE1 to tumorigenesis.

The T lymphoma invasion and metastasis inducing protein 1 (TIAM1) is a guanine nucleotide exchange factor (GEF) that activates the small GTPase RAC (Michiels et al., 1995). It shows perturbed expression in various cancers including colon, breast, and lung (Minard et al., 2005, Stebel et al., 2009, Wang and Wang, 2012). Previously, we showed that *Tiam1* knockout mice are resistant to H-Ras-induced skin tumors (Malliri et al., 2002), implying a requirement for TIAM1 in tumor formation consistent with its roles in cell proliferation and survival (Rygiel et al., 2008). Intriguingly, the few tumors developing in *Tiam1*^−/−^ mice were more frequently malignant (Malliri et al., 2002), suggesting that TIAM1 antagonizes malignant progression. Supporting this, TIAM1-RAC activation restored an epithelial-like phenotype and suppressed invasiveness in RAS-transformed MDCKII cells (Hordijk et al., 1997). Additionally, TIAM1 depletion in nontransformed MDCKII cells lead to the disassembly of their cadherin-based adhesions, acquisition of a flattened morphology and increased motility (Malliri et al., 2004). Collectively, these findings indicate that TIAM1 promotes cadherin-based adhesion. Consistent with a role as an invasion suppressor, TIAM1 protein expression is decreased during breast cancer progression (Stebel et al., 2009). However, the TIAM1-RAC signaling module can also enhance cell migration and invasion through promoting lamellipodia and invadopodia (Bourguignon et al., 2000). Promigratory/proinvasive roles of TIAM1-RAC manifest when cells are unable to form intercellular adhesions, e.g., when plated sparsely or on collagen substrates, or in cells intrinsically lacking E-cadherin, e.g., lymphoma cells (Habets et al., 1995, Sander et al., 1998). Reflecting its functional diversity, TIAM1 protein has been detected at intercellular junctions, the Golgi apparatus, the cytosol, and membrane protrusions (Adams et al., 2010, Mack et al., 2012, Michiels et al., 1995, Woodcock et al., 2009). We postulate that changes in TIAM1 local concentration brought about by the ubiquitin-proteasome pathway could impact upon the resultant outcome of TIAM1 stimulation. Potentially, selective degradation of TIAM1 at cell-cell adhesions triggering their disassembly could preserve the growth, survival, and dissemination stimulatory properties of TIAM1-RAC in malignantly transformed cells, while diminishing their dissemination suppressing properties.

Here, we show that in response to HGF, HUWE1 ubiquitylates TIAM1 on lysine 595, triggering its proteasomal degradation predominantly at cell-cell adhesions, thereby enabling disassembly of cell junctions and induction of cell migration and invasion, including in lung carcinoma cells. We also show that TIAM1 and HUWE1 protein levels are negatively correlated in early-stage lung cancer specimens, consistent with this regulatory mechanism operating in human tumors.

## Results

### HGF Stimulates Proteasomal Degradation of TIAM1 at Cell-Cell Junctions

**Figure 1:**
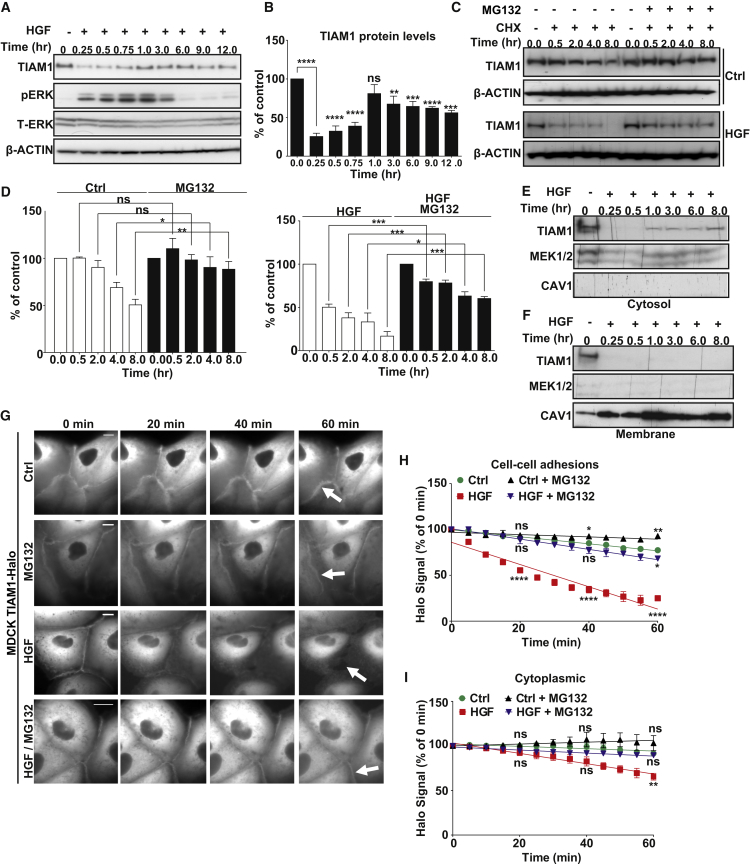
HGF Stimulation Induces TIAM1 Degradation (A) MDCKII cells were treated with 10 ng/ml HGF for the indicated times, and immunoblotting was performed. pERK was used as readout of HGF activity and β-ACTIN and total ERK (T-ERK) as loading controls. (B) Quantification of TIAM1 in (A) from three independent experiments. Mean values ±SE. ^∗∗∗∗^p < 0.0001, ^∗∗∗^p < 0.0005, ^∗∗^p < 0.01; ns, not significant (unpaired t test). (C) MDCKII cells were pretreated with 5 μM MG132 for 3 hr where indicated, and 10 ng/ml HGF and 50 μg/ml cycloheximide (CHX) were added at 0 hr. Protein extracted at the indicated times was analyzed by immunoblotting. (D) Quantification of TIAM1 in (C). Mean values ±SE ^∗∗∗^p < 0.0005, ^∗∗^p < 0.01, and ^∗^p < 0.05; ns, not significant (unpaired t test). (E and F) MDCKII cells were treated with 10 ng/ml HGF for the indicated times. Cytosolic (E) and membrane (F) fractions were prepared and TIAM1 monitored by immunoblotting. MEK1/2 and CAV1 were used as cytosolic and membrane specific markers, respectively. (G) MDCKII cells expressing Halo-tagged TIAM1 were labeled with 50 nM of fluorescent TMR ligand, and Halo-tagged TIAM1 was chased through addition of the HaloTag blocking agent. Live imaging was performed in the presence or absence of HGF for 1 hr. White arrows indicate Halo-tagged TIAM1 at intercellular junctions. Scale bar, 10 μm. (H and I) Fluorescence intensity of Halo-tagged TIAM1 in MDCKII cells was measured for 30 cell-cell adhesions (H) and cytoplasmic pools (I) from three biological replicates. Mean values ±SE ^∗∗∗∗^p < 0.0001, ^∗∗^p < 0.01, ^∗^p < 0.05; ns, not significant (unpaired t test). See also Figure S1.

We reasoned that TIAM1 may be downregulated in response to stimuli that disrupt cell-cell adhesion and induce motility. To test this hypothesis, we utilized MDCKII cells that in response to HGF disassemble their cell-cell adhesions and scatter (Uehara and Kitamura, 1992). We detected a transient and profound decrease of TIAM1 protein during the first hour of HGF treatment, followed by a secondary less-marked reduction persisting to 12 hr after HGF treatment (Figures 1A and 1B). Moreover, we observed scattering of colonies of MDCKII cells within this time frame (Figures S1A and S1B). We then measured the effect of HGF stimulation on the turnover of TIAM1 protein by inhibiting new protein synthesis with cycloheximide. Turnover was greatly increased in cells stimulated with HGF compared to control cells (Figures 1C and 1D). Furthermore, downregulation was via protein degradation as we observed no significant changes in the amount of mRNA following HGF treatment (Figure S1C), and the decrease in TIAM1 protein levels was significantly rescued by treating cells with MG132, a reversible proteasome inhibitor (Figures 1C and 1D).

To investigate if specific subcellular fractions of TIAM1 were subject to preferential degradation, we treated MDCKII cells with HGF and isolated both cytosolic and membrane protein fractions over a time course of 8 hr. A significant decrease of endogenous TIAM1 in both membrane and cytosolic fractions was observed 0.25 hr after HGF treatment (Figures 1E and 1F). However, as seen for the total cell lysate (Figures 1A and 1B), the decrease in cytosolic TIAM1 was partially reversed, whereas the depletion of the membrane fraction was sustained through all subsequent time points (Figures 1E and 1F). To further examine the decrease in TIAM1 seen within the first hour of HGF stimulation, we engineered MDCKII cells to inducibly express Halo-tagged TIAM1 and analyzed the downregulation of TIAM1-Halo at cell-cell adhesions as well as in the cytoplasm using a pulse-chase method described elsewhere (Yamaguchi et al., 2009). We detected significant TIAM1 depletion from cell-cell adhesions but less so from the cytoplasm of HGF-stimulated cells (Figures 1G–1I). Furthermore, TIAM1 depletion could be rescued by inhibiting the proteasome (Figures 1G–1I). We therefore conclude that HGF stimulation induces the preferential depletion of TIAM1 from cell-cell adhesions.

### TIAM1 Is Ubiquitylated in HGF-Stimulated Cells

**Figure 2:**
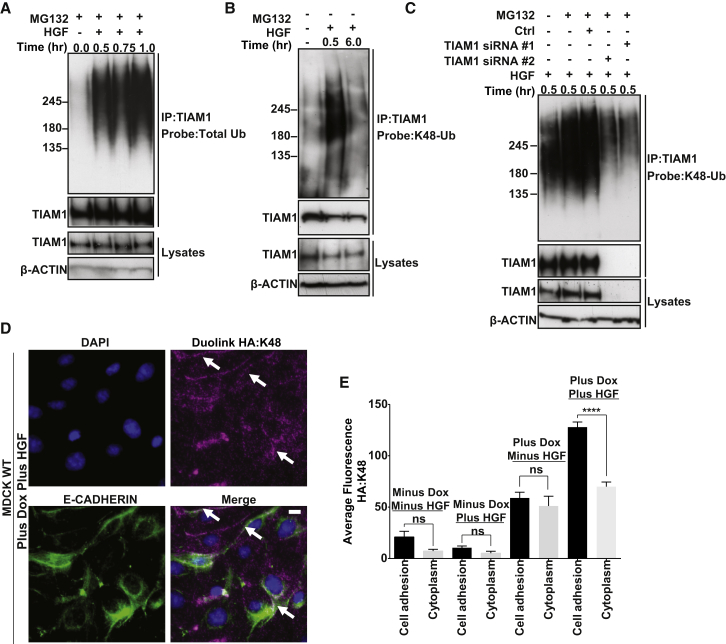
TIAM1 Is Ubiquitylated following HGF Stimulation (A) MDCKII cells were pretreated (3 hr) with 5 μM MG132 and treated with 10 ng/ml HGF for the times indicated. Cells were then lysed and endogenous TIAM1 immunoprecipitated (IP) under denaturing conditions and probed for total ubiquitin. (B) As for (A) but without pretreatment with MG132 and immunoprecipitates were probed using a K48-linkage specific antibody. (C) MDCKII cells were transfected with a nontargeting siRNA or two siRNAs targeting TIAM1 (#1 and #2). At 96 hr following transfection, cells were treated with 10 ng/ml HGF and protein was extracted at 0.5 hr. TIAM1 was immunoprecipitated under denaturing conditions and probed with a K48-linkage specific antibody. (D) MDCKII cells were pretreated (3 hr) with 5 μM MG132 and with 10 ng/ml HGF for 1 hr and fixed, and the Duolink protocol was performed to monitor the extent and localization of K48 ubiquitylated TIAM1-HA induced following addition of doxycycline (Plus Dox). White arrows indicate intercellular junctions. Scale bar, 10 μm. (E) Quantification of Duolink signal in (D) and also in uninduced (Minus Dox) cells. Mean values ±SE of three independent experiments. ^∗∗∗∗^p < 0.0001; ns, not significant (unpaired t test). See also Figure S2.

We next tested whether TIAM1 was modified by ubiquitin (Ub) during the initiation of HGF-induced cell scattering. Endogenous TIAM1 was immunoprecipitated under denaturing conditions and probed for ubiquitin. A significant accumulation of ubiquitylated TIAM1 was observed following HGF treatment compared to untreated control (Figure 2A). K48-Ub chains are considered the primary signal for proteasomal degradation, and attachment of four or more Ub molecules to the protein is sufficient to target proteins to the proteasome (Chau et al., 1989). Using a specific K48-linked ubiquitin antibody to probe TIAM1 immunoprecipitated under denaturing conditions, we detected a significant increase of K48-linked TIAM1 after HGF treatment (Figure 2B). Moreover, this ubiquitin smear was specific to TIAM1, because downregulating TIAM1 using two different small interfering RNAs (siRNAs) substantially decreased it (Figure 2C). As further confirmation, having engineered MDCKII cells to express HA-tagged TIAM1 following addition of doxycycline, the Duolink proximity ligation assay (Duolink PLA) revealed a large increase in TIAM1-HA ubiquitylated with K48-linked Ub in HGF-stimulated cells compared to untreated cells (Figures 2D, 2E, and S2). Although TIAM1 ubiquitylated with K48-linked Ub was distributed throughout the cell, it was enriched at cell-cell adhesions (Figures 2D, 2E, and S2, see white arrows). These data suggest that TIAM1 is ubiquitylated and degraded via K48-linked Ub during the early stages of epithelial cell scattering, and that this degradation occurs more at cell-cell adhesions than in the cytoplasm.

### The E3 Ligase HUWE1 Ubiquitylates TIAM1 in HGF-Stimulated Cells

**Figure 3:**
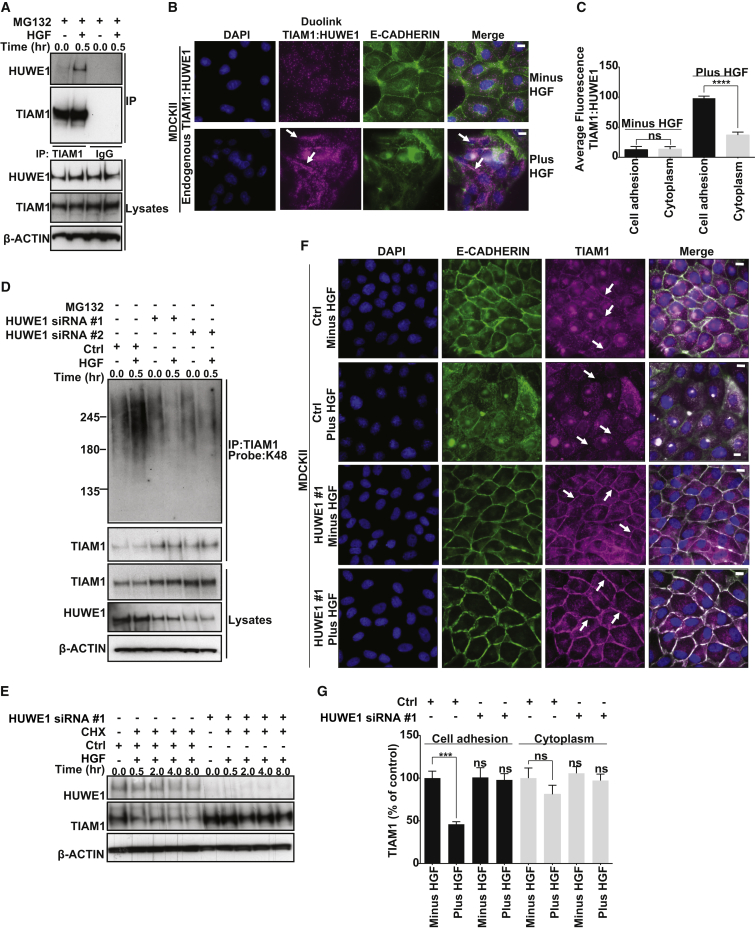
TIAM1 Is Ubiquitylated by HUWE1 (A) MDCKII cells were pretreated (3 hr) with 5 μM MG132 and treated with 10 ng/ml HGF as shown, and protein was extracted at the times indicated. Endogenous TIAM1 was immunoprecipitated, and lysates were probed for HUWE1. Lysates were also subjected to IP with an immunoglobulin G control. (B) MDCKII cells were pretreated with 5 μM MG132 for 3 hr with (Plus HGF) or without (Minus HGF) the addition of 10 ng/ml HGF for 1 hr and fixed, and the Duolink protocol was performed to monitor the extent and localization of the HUWE1 interaction with TIAM1 (both endogenous). White arrows indicate intercellular junctions. Scale bar, 10 μm. (C) Quantification of the Duolink signal in (B). Mean values ±SE of three independent experiments. ^∗∗∗∗^p < 0.0001; ns, not significant (unpaired t test). (D) MDCKII cells were transfected with HUWE1 siRNA #1 or #2 or a nontargeting siRNA (Ctrl). At 96 hr posttransfection, cells were treated with 10 ng/ml HGF for 0.5 hr where indicated. TIAM1 was immunoprecipitated under denaturing conditions and probed with a K48-linkage specific antibody. (E) MDCKII cells were transfected with HUWE1 siRNA #1 or a nontargeting siRNA (Ctrl). Cycloheximide (CHX) (50 μg/ml) was added at 96 hr posttransfection, and cells were treated with HGF for the indicated times. Lysates were immunoblotted for TIAM1 and HUWE1. In (A), (D), and (E), β-ACTIN was used as a loading control. (F) MDCKII cells were transfected with HUWE1 siRNA #1 or a nontargeting siRNA (Ctrl). At 72 hr posttransfection, cells were stimulated with HGF for 1 hr as indicated and fixed. Cells were stained by immunofluorescence (IF) for E-CADHERIN, TIAM1, and DAPI. Panels show representative images from one of three independent experiments. Scale bar, 10 μm. (G) Quantification of TIAM1 IF levels in (F). Mean values ±SE of three independent experiments. ^∗∗∗^p < 0.0005; ns, not significant (unpaired t test). See also Figure S3.

We previously performed tandem affinity purification (TAP) of protein complexes from *Tiam1*^−/−^ mouse embryonic fibroblasts expressing TAP-tagged TIAM1 (Mack et al., 2012). This approach also identified the HUWE1 E3 ubiquitin ligase as a binding partner of TIAM1 (data not shown). This interaction was validated in vivo using coimmunoprecipitation (co-IP) both with endogenous proteins in MDCKII cells and exogenous TIAM1 in HEK293T cells (Figure 3A; Figure S3A, respectively). Notably, the interaction of TIAM1 and HUWE1 was greatly increased in the presence of HGF (Figure 3A). Immunofluorescence revealed that, in addition to its previously described nuclear localization, HUWE1 in MDCKII cells could be clearly observed at cell-cell junctions, colocalizing with both E-CADHERIN and TIAM1 (Figure S3B). The Duolink PLA assay also showed a marked increase in the interaction of endogenous TIAM1 and endogenous HUWE1 following HGF stimulation, which again while visualized throughout the cell was significantly more pronounced along cell-cell adhesions (Figures 3B and 3C).

The documented role of HUWE1 as an E3 ligase indicates that it could be responsible for regulating the stability of TIAM1 through its ubiquitylation activity. We therefore depleted HUWE1 using different shRNA and siRNA sequences. This resulted in stabilization of TIAM1 protein (Figures S3C and S3D) but not mRNA (Figure S3E) and a concomitant reduction of TIAM1 K48-linked ubiquitylation (Figures 3D and S3F–S3H). Moreover, the increased turnover of TIAM1 in cells stimulated with HGF was suppressed by HUWE1 depletion (Figures 3E, S3I, and S3J). Further, immunofluorescence was performed in either control or HUWE1-depleted cells before and after HGF treatment (Figure 3F). In cells treated with a nontargeting control oligo, TIAM1 was significantly depleted from cell-cell adhesions but not the cytoplasm following HGF stimulation (Figures 3F and 3G). Significantly, depletion of HUWE1 was able to stabilize TIAM1 levels at cell-cell adhesions in HGF-treated cells (Figures 3F and 3G). These observations strongly imply that HUWE1 is largely responsible for the polyubiquitylation and degradation of TIAM1 at cell-cell adhesions during the induction of epithelial cell scattering.

### HUWE1-Mediated TIAM1 Degradation Controls HGF-Induced Motility and Invasion of Epithelial Cells

**Figure 4:**
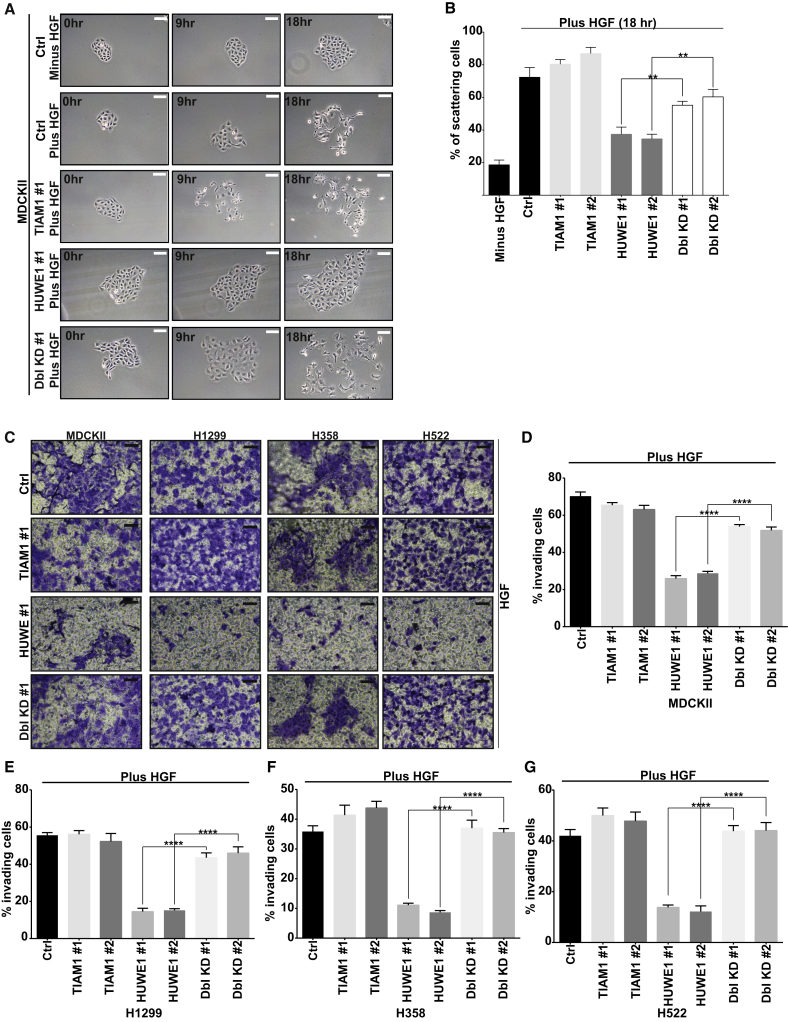
HUWE1-Mediated TIAM1 Degradation Controls HGF-Induced Motility and Invasion (A) MDCKII cells were transfected with a nontargeting siRNA (Ctrl) or with siRNAs targeting TIAM1 or HUWE1 alone or in combination. Ninety-six hours later, cells were stimulated with 20 ng/ml HGF for 18 hr. Resultant cell scattering was monitored by phase contrast microscopy. Scale bar, 50 μm. (B) Quantification of (A). Percentage of cell scattering was calculated by counting the percentage of cells with less than three cell-cell adhesions remaining in each colony. At least ten colonies were counted in each of three independent biological replicates. ^∗∗^p < 0.001 (unpaired t test). (C) MDCKII, H1299, H358, and H522 cells were transfected with a nontargeting siRNA (Ctrl) or siRNAs targeting TIAM1 or HUWE1 alone or in combination and seeded in a modified Boyden chamber coated with collagen I to assay for invasion in the presence of 10 ng/ml HGF. After 1 day, invading cells were stained with crystal violet. Panels show representative images from one of at least three independent experiments. Scale bar, 150 μm. (D–G) Crystal violet from (C) was extracted and absorbance measured at 600 nm. Relative invasion was determined for (D) MDCKII (E) H1299, (F) H358, and (G) H522 cells by relating optical density to a standard curve of the appropriate cells and normalizing this to total cell number for each condition and cell line. Mean values ±SE from three independent experiments. ^∗∗∗∗^p < 0.0001 (unpaired t test). See also Figure S4.

To investigate the potential role of HUWE1 in cell-cell adhesion and migration and whether this might be through regulating TIAM1, we depleted HUWE1 and TIAM1 alone and in combination and assessed the effect on HGF-induced cell motility. Cell scattering was monitored by phase contrast microscopy (Figures 4A and S4A) and cells were lysed at 18 hr to confirm TIAM1 and HUWE1 knockdown by immunoblotting (Figure S4B). MDCKII cells with depleted HUWE1 scattered significantly less than cells transfected with the nontargeting control, whereas, in contrast, scattering in cells with depleted TIAM1 was moderately enhanced following HGF treatment (Figures 4A, 4B, and S4A). To examine if the decreased scattering of HUWE1-depleted cells was due to an increase in TIAM1 at cell-cell adhesions (as seen in Figures 3F and 3G), we knocked down both HUWE1 and TIAM1 using two different combinations of siRNA in MDCKII cells. Interestingly, simultaneous depletion of both TIAM1 and HUWE1 significantly rescued the scattering of MDCKII cells (Figures 4A, 4B, and S4A).

Next, we investigated the role of HUWE1-mediated TIAM1 degradation in cell invasion. Seventy-two hours posttransfection, MDCKII cells in which HUWE1, TIAM1, or a combination of both were depleted were assayed for short-term (24 hr) and long-term (120 hr) invasive potential using modified Boyden chamber assays coated with collagen. Transfected cells were also seeded in 24-well plates to measure total cell number (in order to normalize cellular invasion to cell number) and 6-well plates to measure knockdown of TIAM1 and HUWE1 protein expression (Figure S4C, top) at the end of the experiment. Invasion was quantified by crystal violet staining of cells, which had invaded through both collagen and insert membrane and normalized to total cell number. Both short-term (Figures 4C and 4D) and long-term (Figures S4D and S4E) invasion assays resulted in similar patterns of invasion. Depletion of HUWE1 alone greatly inhibited the invasion of MDCKII cells stimulated by HGF. In contrast, the invasion of TIAM1-depleted cells showed no significant change (Figures 4C, 4D, S4D, and S4E). Interestingly, the ability of MDCKII cells to invade was significantly restored in cells where both HUWE1 and TIAM1 were downregulated simultaneously (Figures 4C, 4D, S4D, and S4E).

The above findings in MDCKII cells raised the possibility that aberrant HUWE1 regulation of TIAM1 might occur during tumorigenesis, potentially contributing to malignant progression. HUWE1 is significantly overexpressed in a number of epithelial tumors including lung carcinoma (Confalonieri et al., 2009). However, no invasive role had thus far been attributed to HUWE1. To understand if the above mechanism is relevant in lung carcinoma, we obtained a number of lung carcinoma cell lines, including the H1299, H358, H522, and H596 cell lines, which contain nonfunctional p53 (as depletion of HUWE1 in some settings has been shown to result in stabilization of wild-type p53 and consequent cell death [Kon et al., 2012]). Similar to MDCKII cells, depletion of HUWE1 in H1299, H358, H522, and H596 cell lines resulted in increased TIAM1 protein levels (Figure S4C; data not shown). Further and again comparable to the phenotype we observed in MDCKII cells, depletion of HUWE1 in H1299, H358, H522, and H596 cell lines significantly inhibited HGF-induced invasion. In contrast, TIAM1 depletion did not significantly affect invasiveness (Figures 4C, 4E–4G, S4D, and S4F; data not shown). Again, the ability of H1299, H358, H522, and H596 cells to invade was significantly restored in cells where both proteins were downregulated simultaneously (Figures 4C, 4E–4G, S4D, and S4F; data not shown). These results reveal a role for HUWE1 in the invasion of lung carcinoma cells through mediating degradation of TIAM1.

### TIAM1 Is Ubiquitylated on Lysine Residue 595

**Figure 5:**
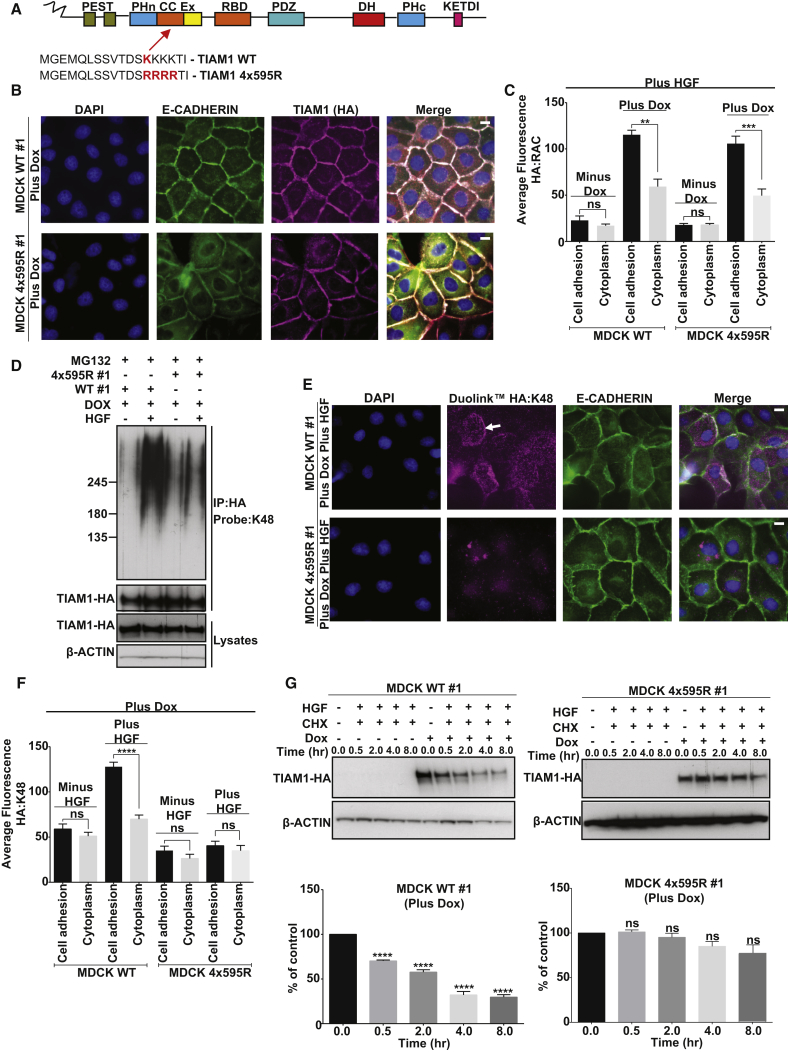
TIAM1 Is Ubiquitylated at K595 (A) Schematic representation of TIAM1 with the lysine 595 ubiquitylation site highlighted in red. (B) MDCKII cells inducibly expressing (Plus Dox) either WT HA-tagged TIAM1 (WT #1) or mutant HA-tagged 4x595R TIAM1 (4x595R #1) were seeded on glass coverslips for 48 hr, fixed, and stained by IF for E-CADHERIN, TIAM1-HA, and DAPI. Representative images from MDCK WT #1 and MDCK 4x595R #1 clones are shown. Scale bar, 10 μm. (C) Quantification of RAC:TIAM1-HA Duolink signal generated by seeding MDCKII cells noninduced (Minus Dox) or inducibly expressing (Plus Dox) either WT TIAM1 (WT #1) or mutant 4x595R TIAM1 (4x595R #1) and stimulating with 10 ng/ml HGF for 30 min. (D) MDCKII cells inducibly expressing (+ Dox) either WT HA-tagged TIAM1 (WT #1) or mutant HA-tagged 4x595R TIAM1 (4x595R #1) were treated, where indicated, with 10 ng/ml HGF, and protein was extracted at 30 min. HA-tagged TIAM1 was immunoprecipitated under denaturing conditions and probed with a K48-linkage specific antibody. (E) MDCKII cells inducibly expressing (Plus Dox) either HA-tagged WT TIAM1 or 4x595R mutant TIAM1 were pretreated for 3 hr with 5 μM MG132 and treated with 20 ng/ml HGF for 1 hr and fixed, and the Duolink protocol was performed to monitor the extent and localization of K48 ubiquitylated TIAM1. White arrow indicates intercellular junctions. Scale bar, 10 μm. (F) Quantification of Duolink signal in (E) and also in unstimulated cells (Minus HGF). Mean values ±SE ^∗∗∗∗^p < 0.0001; ns, not significant (unpaired t test). (G) MDCKII cells noninduced (− Dox) or inducibly expressing (+ Dox) either WT TIAM1 (WT #1) or mutant 4x595R TIAM1 (4x595R #1) were treated with 50 μg/ml cycloheximide (CHX) and HGF (10 ng/ml) for the indicated times after which protein was extracted. Lysates were immunoblotted for TIAM1-HA. Quantification of TIAM1 from three independent experiments. β-ACTIN was used as a loading control in (D) and (G). Mean values ±SE ^∗∗∗∗^p < 0.0001; ns, not significant (unpaired t test). See also Figure S5.

Using mass spectrometry, exogenous TIAM1 immunoprecipitated from HEK293T cells was found to be ubiquitylated by HUWE1 at lysine 595 (K595) (Figure S5A) located in the MGEMQLSSVTDSKKKKTI peptide in the coiled coil region (Figure 5A). In vitro ubiquitylation assays revealed a ladder consistent with the attachment of ubiquitin to TIAM1, which was absent when Ub or ATP was not present (Figure S5B, left) and again mass spectrometry identified lysine 595 of TIAM1 as a HUWE1 ubiquitylation site (data not shown). p53, a previously identified substrate of HUWE1, was used as a positive control for the reaction (Figure S5B, right).

To confirm that K595 acts as a bona fide acceptor site for ubiquitin, site-directed mutagenesis was performed to construct a TIAM1 ubiquitylation mutant. K595 and three lysine residues that follow were mutated to arginine (Figure 5A) to suppress lysine acceptor promiscuity, which has been previously documented (Danielsen et al., 2011). MDCKII cells were engineered to allow doxycycline (Dox)-induced expression of either wild-type (WT) or 4x595R mutant TIAM1 (4x595R). Both 4x595R and WT TIAM1 were clearly observed to colocalize with E-CADHERIN at cell-cell adhesions (Figures 5B and S5C). Furthermore, we found that following HGF treatment the 4x595R mutant TIAM1 was able to both interact with RAC along cell-cell adhesions (Figures 5C and S5D) and activate RAC similarly to WT TIAM1 (Figure S5E). Thus, 4x595R mutant TIAM1 functions similarly to WT TIAM1 in these settings. To investigate if the 4x595R mutant TIAM1 was however defective in ubiquitylation and degradation, expression of HA-tagged WT and 4x595R mutant TIAM1 were induced in MDCKII cells that were subsequently pretreated for 3 hr with MG132 and stimulated with HGF for 30 min. HA-tagged proteins were immunoprecipitated and probed for K48-linked ubiquitin. HGF treatment promoted K48-linked ubiquitylation of WT, but not of 4x595R mutant TIAM1 (Figures 5D and S5F). Furthermore, the Duolink PLA assay revealed that, whereas WT TIAM1 and K48-Ub interacted extensively in HGF-stimulated cells and this interaction was enriched along cell-cell adhesions (Figures 5E, top, 5F, and S5G, top), 4x595R TIAM1 and K48-Ub showed only limited interaction, which did not appear to be localized to cell-cell adhesions (Figures 5E, bottom, 5F, and S5G, bottom). Moreover, MDCK 4x595R cells showed significantly increased TIAM1 stability compared to MDCK WT cells (Figures 5G and S5H). These data demonstrate that mutation of the K595 ubiquitylation site on TIAM1 results in a considerable decrease of HUWE1-mediated K48-linked ubiquitylation along cell-cell adhesions and a resultant increase in overall protein stability.

### Ubiquitylation of TIAM1 at Lysine 595 Regulates Cell-Cell Adhesion Disassembly, Scattering, and Invasion

**Figure 6:**
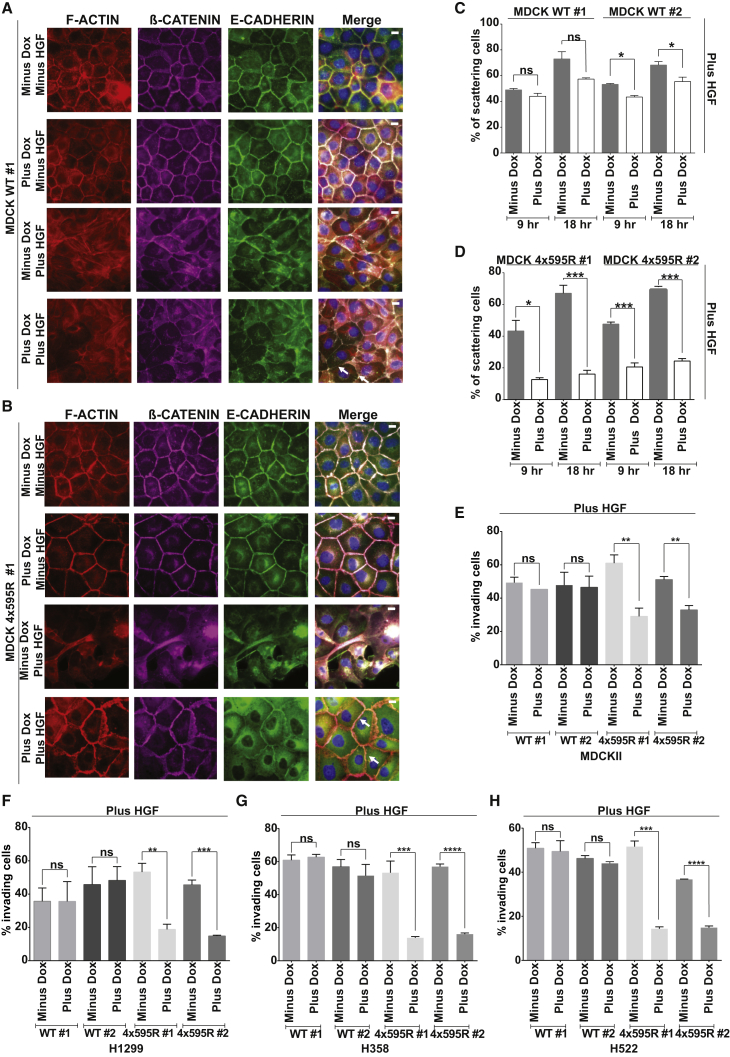
Ubiquitylation of TIAM1 at Lysine 595 Regulates Cell-Cell Adhesion Disassembly, Migration, and Invasion (A and B) MDCKII cells noninduced (Minus Dox) or inducibly expressing (Plus Dox) either TIAM1 WT (WT #1) (A) or TIAM1 4x595R (4x595R #1) (B) were incubated for 1 hr in the presence (Plus HGF) or absence (Minus HGF) of 20 ng/ml HGF, fixed, and costained by IF for F-ACTIN, E-CADHERIN, β-CATENIN, and DAPI. Representative images from one of two independent MDCKII WT and 4x595R clones are shown. Scale bar, 10 μm. (C and D) MDCKII cells noninduced (Minus Dox) or inducibly expressing (Plus Dox) either TIAM1 WT (WT #1 and WT #2) or TIAM1 4x595R (4x595R #1 and 4x595R #2) were incubated for the indicated times with 20 ng/ml HGF. Cell scattering was monitored by phase contrast microscopy and quantitation performed for two independent MDCK WT (C) and MDCK 4x595R (D) clones by calculating the percentage of cells with less than three cell-cell adhesions remaining in each colony. At least ten colonies were counted in each of three independent biological replicates. ^∗^p < 0.05, ^∗∗∗^p < 0.0005; ns, not significant (unpaired t test). (E–H) Noninduced (Minus Dox) MDCKII, H1299, H358, and H522 cells or the same cells inducibly expressing (Plus Dox) either TIAM1 WT or TIAM1 4x595R were seeded in a modified Boyden chamber to assay for invasion in the presence of 10 ng/ml HGF. Cells were left to invade for 1 day, fixed, and stained with crystal violet. Crystal violet was eluted, absorbance was measured at 600 nm, and relative invasion was determined for (E) MDCKII, (F) H1299, (G) H358, and (H) H522 by relating optical density to a standard curve of the appropriate cell type and then normalizing this to total cell number for each condition. Mean values ±SE ^∗∗^p < 0.001, ^∗∗∗^p < 0.0005, ^∗∗∗∗^p < 0.0001; ns, not significant (unpaired t test). See also Figure S6.

To further investigate the connection between TIAM1 ubiquitylation and cell-cell adhesion disassembly and migration, we compared MDCK WT to MDCK 4x595R cells in their ability to disassemble their cell-cell junctions following HGF stimulation. In unstimulated MDCKII cells in which expression of either WT or 4x595R mutant TIAM1 was not induced (minus Dox, minus HGF), E-CADHERIN, β-CATENIN, and F-ACTIN were localized at cell-cell contacts forming a characteristic honeycomb pattern (Figures 6A and 6B, top row). Unstimulated MDCKII cells in which WT or 4x595R mutant TIAM1 expression had been induced (plus Dox, minus HGF) exhibited a phenotype similar to that of control cells (minus Dox, minus HGF) (Figures 6A and 6B, second row). Treatment of noninduced cells with HGF for 1 hr (minus Dox, plus HGF) resulted in disassembly of cell-cell junctions accompanied by a redistribution of junctional markers that acquired intracellular localization (Figures 6A and 6B, third row). HGF-stimulated and induced MDCK WT cells (plus Dox, plus HGF) behaved similarly to their noninduced counterparts (minus Dox, plus HGF; Figure 6A, fourth row, see white arrows) becoming less compact and disassembling their cell-cell junctions. In contrast, disassembly of cell-cell adhesions was markedly delayed in HGF-treated and induced MDCK 4x595R cells (plus Dox, plus HGF), which retained their junctional organization as visualized by E-CADHERIN, F-ACTIN, and β-CATENIN staining (Figure 6B, fourth row, see white arrows).

To extend these observations, we compared the scattering of MDCK WT and 4x595R cells. Induced MDCK WT cells scattered following HGF treatment for either 9 or 18 hr (Figures 6C and S6A), whereas induced 4x595R cells showed a substantial decrease in scattering at both time points (Figures 6D and S6B). Protein levels were measured to ensure equivalent TIAM1 expression in doxycycline-induced MDCK WT and 4x595R cells (Figure S6C). Together, these results suggest that HUWE1-mediated ubiquitylation of TIAM1 on lysine 595 regulates HGF-induced cell-cell adhesion disassembly and scattering in MDCKII cells.

We next investigated whether the reduced scattering of cells expressing the 4x595R ubiquitylation defective TIAM1 mutant would lead to impaired cell invasion. Both short- (24 hr; Figures 6E and S6D) and long-term (120 hr; data not shown) invasion assays were performed as described above. Neither MDCK WT nor 4x595R cells invaded without HGF (data not shown). Moreover, noninduced MDCK WT and 4x595R cells readily invaded when stimulated with HGF (minus Dox; Figures 6E and S6D). Likewise, MDCK WT clones induced to express WT TIAM1 also invaded following HGF stimulation (plus Dox; Figures 6E and S6D). However, MDCK 4x595R cells induced to express the 4x595R mutant (plus Dox) invaded significantly less than noninduced 4x595R (minus Dox) or induced WT cells (plus Dox) stimulated with HGF (Figures 6E and S6D). Once again, to understand if the same mechanism is operating in lung carcinoma cells, we performed invasion assays in H1299, H358, and H522 lung cancer cell lines overexpressing either WT or 4x595R TIAM1. As for parental cells, no significant invasion of cells expressing either WT or 4x595R TIAM1 was seen without HGF stimulation (data not shown). However, both noninduced WT and 4x595R cells (minus Dox) as well as cell lines induced to express WT TIAM1 (plus Dox) readily invaded with HGF. In contrast, cells induced to express 4x595R TIAM1 (plus Dox) invaded significantly less than noninduced 4x595R (minus Dox) or induced WT cells (plus Dox) stimulated with HGF (Figures 6F–6H and S6E–S6G). Western blotting was performed to ensure equivalent expression of WT and 4x595R TIAM1 (Figures S6H–S6J). Based on this evidence, we conclude that aberrant HUWE1-mediated ubiquitylation of TIAM1 could contribute to the enhanced invasiveness of lung carcinoma cells.

**Figure 7:**
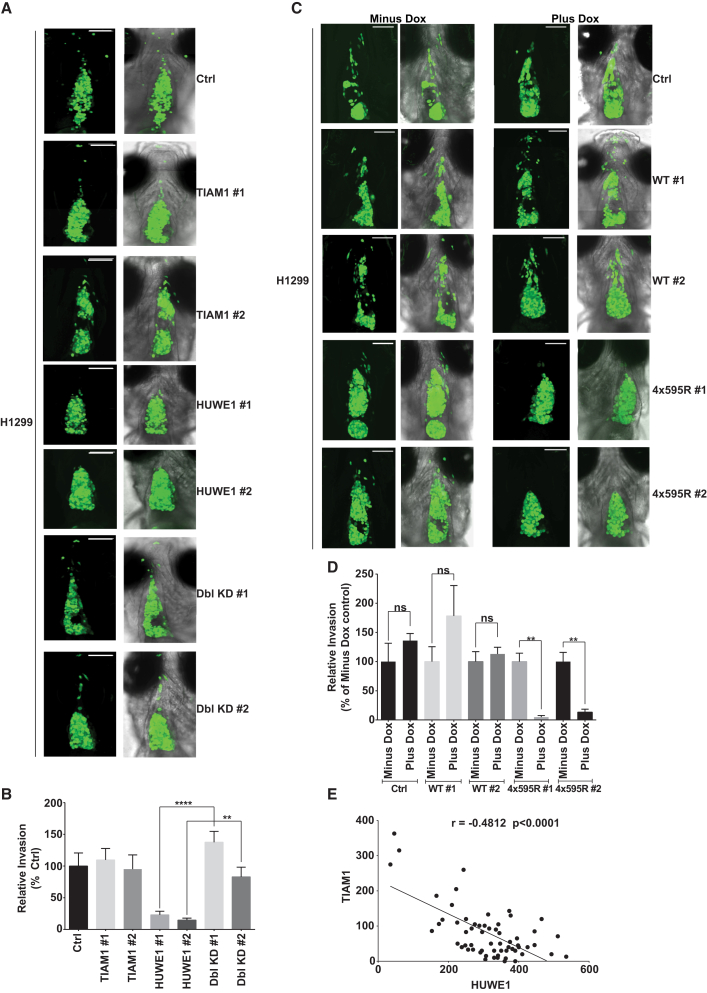
HUWE1-Mediated TIAM1 Degradation Controls Lung Carcinoma Cell Invasion In Vivo and HUWE1 and TIAM1 Are Negatively Correlated in Stage I and II Lung Carcinoma (A) Green fluorescent H1299 cells were transfected with a nontargeting siRNA (Ctrl) or siRNAs targeting TIAM1 or HUWE1 alone or in combination and injected into the pericardial cavity of zebrafish embryos. Xenografted cells were imaged at 4 days postinjection (dpi). Right column of images show merge with bright field. Scale bar, 100 μm. (B) Quantitation of invasion depicted in (A). Invasion was normalized to Ctrl. Mean values ±SE from three independent experiments. ^∗∗^p < 0.01, ^∗∗∗∗^p < 0.0001 (Kruskal-Wallis one-way analysis of variance). (C) Parental green fluorescent H1299 cells (Ctrl), noninduced (Minus Dox), or inducibly expressing (Plus Dox) either WT TIAM1 (WT #1 and WT #2) or 4x595R TIAM1 (4x595R #1 and 4x595R #2) were injected into the pericardial cavity of zebrafish embryos. Xenografted cells were imaged at 4 dpi. Second and fourth column of images show merge with bright field. Scale bar, 100 μm. (D) Quantitation of invasion depicted in (C). In each case, invasion was normalized to the corresponding Minus Dox. Mean values ±SE from three independent experiments. ^∗∗^p < 0.01 (Kruskal-Wallis one-way analysis of variance). (E) Scatterplot depicting the statistically significant Spearman’s negative correlation between HUWE1 and TIAM1 in corresponding tissue sections. See also Figure S7.

The results above indicate that HUWE1 plays a role in cell-cell adhesion, motility, and invasion of epithelial cells in vitro by regulating TIAM1. To investigate this role in vivo, we used a zebrafish xenograft model. Fluorescently labeled H1299 cells were injected into the pericardial cavity of 2-day-old zebrafish embryos. Seventy-two hours postinjection, tumor cells were observed to have largely filled the approximately conical shaped cavity. Several cells were also observed to have disseminated outside the pericardial cavity, considered to have undergone local invasion (Figure S7A). H1299 cells treated with both a nontargeting (Ctrl) siRNA and TIAM1 targeting siRNAs also disseminated out of the pericardial cavity. However, dissemination of H1299 cells in which HUWE1 was depleted was significantly reduced (Figures 7A and 7B). Remarkably, the ability of H1299 cells to disseminate was completely restored when HUWE1 and TIAM1 were downregulated simultaneously (Figures 7A and 7B). To further examine the requirement of TIAM1 ubiquitylation for invasion in vivo, we injected H1299 cells expressing either WT or 4x595R TIAM1 into the pericardial cavity. All noninduced (minus Dox) H1299 cells and also H1299 cells expressing WT TIAM1 (plus Dox) disseminated away from the pericardial region similarly to parental H1299 cells (Ctrl), whereas cells expressing the nondegradable 4x595R TIAM1 (plus Dox) showed a strikingly diminished ability to disseminate (Figures 7C and 7D). Thus, HUWE1 appears essential for the invasion of lung carcinoma cells and this is entirely dependent on ubiquitylation of TIAM1.

### HUWE1 Expression Is Negatively Correlated with TIAM1 in Squamous Cell Lung Carcinomas

To substantiate the clinical relevance of the above findings in lung carcinoma, we obtained tissue microarrays containing tumor samples from stage I and stage II squamous cell lung carcinoma patients. These were stained using immunohistochemistry (IHC) with specific antibodies recognizing HUWE1, c-MET, and TIAM1. (c-MET expression has already been shown to correlate with poor patient outcome in lung cancer [Siegfried et al., 1997].) In this cohort, 94.3%, 79.8%, and 98.6% of patients were positive for c-MET, TIAM1, and HUWE1 expression, respectively. Example images of lung cancer specimens with low and high HUWE1, TIAM1, and c-MET expression are shown in Figure S7B. A positive and statistically significant correlation was observed between HUWE1 and c-MET (Spearman coefficient r = 0.3953, p = 0.0011, 95% confidence interval [CI] 0.1518–0.5935; Figure S7C), and, strikingly, a strong inverse correlation was observed between HUWE1 and TIAM1 expression (Spearman coefficient r = −0.4812, p < 0.0001, 95% CI −0.6582 to −0.2539; Figure 7E) and TIAM1 and c-MET expression (Spearman coefficient r = −0.3497, p = 0.0057, 95% CI −0.5582 to −0.09977; Figure S7D). Taken together, these results suggest that the spatiotemporal regulation of TIAM1 protein expression by the E3 ligase HUWE1 could be deregulated during lung tumorigenesis.

## Discussion

In this study, we have demonstrated a critical role for the E3 ligase HUWE1 in regulating cell-cell adhesion, cell motility, and invasion. We show that in epithelial cells, including lung carcinoma cells, stimulated with HGF, TIAM1—a critical regulator of cadherin-associated adhesion— is rapidly targeted for proteasome-dependent degradation via HUWE1-mediated ubiquitylation. Failure to degrade TIAM1 or depletion of HUWE1 in cells results in delayed adhesion disassembly and prevents the HGF-induced stimulation of migration and invasion. These findings reveal a molecular mechanism by which MET/HGF signaling, whose hyperactivation is associated with invasive growth of many neoplasms, stimulates epithelial cell motility and invasion.

Intriguingly, both oncogenic (Adhikary et al., 2005) and tumor suppressor (Inoue et al., 2013) roles have been attributed to HUWE1. HUWE1 is overexpressed in cancers of the lung, breast, colon, prostate, liver, pancreas, and thyroid but downregulated in stomach and uterine cancer as well as glioblastomas (Adhikary et al., 2005, Confalonieri et al., 2009). The conflicting reports surrounding HUWE1 function can in part be explained by the nature of its varying substrates, which are involved in a wide variety of processes including apoptosis (p53, [Adhikary et al., 2005]), DNA damage response (CDC6 [Hall et al., 2007]), and transcriptional regulation (e.g., p53, c-MYC [Adhikary et al., 2005, Inoue et al., 2013]). Our findings here suggest that effects of HUWE1 on cell-cell adhesion and invasion via regulating TIAM1 degradation would promote epithelial tumor progression.

Although both HUWE1 and TIAM1 are expressed in most tissues, it is possible that the mechanism described here for regulating migration is restricted to cells capable of forming cell-cell adhesions, because it is the pool of TIAM1 expressed at cell-cell junctions that appears to be preferentially degraded by HUWE1, allowing cells to move away from each other and become motile and invasive. It is therefore interesting that many solid tumors of epithelial origin demonstrate deregulation of both HUWE1 and TIAM1 protein expression. Indeed, we have shown here that an inverse correlation exists between TIAM1 and HUWE1 (and TIAM1 and c-MET) in squamous cell lung carcinoma. We speculate that, in neoplasms characterized by aberrant HGF/MET signaling, HUWE1 overexpression results in an increased turnover of TIAM1 at cell-cell adhesions, permitting junction disassembly and stimulating cell motility and invasion—steps vital under certain circumstances for the initiation of the metastatic cascade. Potentially, pharmacological agents that disrupt the HUWE1-TIAM1 interaction could be beneficial in decreasing the HGF/MET-driven metastatic dissemination of cancer cells.

The TIAM1-RAC signaling module is detected at various subcellular compartments and orchestrates multiple cellular processes. Spatiotemporal modulation of TIAM1 and RAC stability and/or activity is likely to be an important means of regulating TIAM1-RAC signaling. From our data, we infer that HUWE1-mediated ubiquitylation is an effective mechanism to achieve spatiotemporal modulation of TIAM1 protein levels and thereby regulate motility selectively. In our proposed model (Figure S7E), TIAM1 primarily at cell-cell adhesions is targeted for proteasomal degradation in cells stimulated with HGF by HUWE1-mediated ubiquitylation, sparing a significant cytoplasmic pool, which potentially could continue to promote cell growth, survival, and migration. Alternative mechanisms for controlling TIAM1 stability have emerged. Previously, we have shown that calpain cleavage of TIAM1 also regulates TIAM1 stability at adherens junctions but in the context of oncogenic SRC activation (Woodcock et al., 2009). Recently, it was shown that TIAM1 can also be targeted for ubiquitylation by SCF ubiquitin ligase containing β-TRCP (Magliozzi et al., 2014, Zhu et al., 2014). Intriguingly, we found that β-TRCP depletion did not impair HGF-induced scattering of MDCKII cells (data not shown), most likely due to targeting of a different pool of TIAM1 by β-TRCP compared to HUWE1. We have also recently shown ubiquitylation of RAC following HGF treatment of MDCKII cells mediated by the E3 ligase HACE (Castillo-Lluva et al., 2013). Thus, regulation of TIAM1-RAC signaling through ubiquitylation and proteasomal degradation appears pivotal to the rapid response of cells to extracellular signals.

In conclusion, we show that the E3 ligase HUWE1 is a regulator of cell-cell adhesion, migration, and invasion through the spatiotemporal modulation of the TIAM1 signaling network. In epithelial cells with activated c-MET or potentially simply overexpressing HUWE1, ubiquitylation of TIAM1 at cell-cell junctions facilitates both cell-cell adhesion disassembly and invasion, thus overcoming the dissemination suppressing properties previously associated with the TIAM1/RAC signaling axis. Because ubiquitin-coupled degradation of numerous junctional proteins now appears critical in epithelial dedifferentiation and acquisition of a motile and invasive phenotype, pharmacological interventions targeting the ubiquitin-proteasome pathway could impact on carcinoma cell invasion and metastasis.

## Experimental Procedures

Constructs, antibodies, cell lines, and siRNA sequences are described in detail in the Supplemental Experimental Procedures.

### Protein Analysis

Cells were lysed in lysis buffer (50 mM Tris-HCl [pH 7.5], 150 mM NaCl, 1% [v/v] Triton X-100, 10% [v/v] glycerol, 2 mM EDTA, 25 mM NaF, and 2 mM NaH_2_PO4) containing protease and phosphatase inhibitor cocktails (Sigma) and proteins resolved by SDS-PAGE for western blotting. In protein turnover experiments, cells were treated with 50 μg/ml CHX for 0–8 hr prior to lysis. For proteasomal inhibition, cells were incubated for 3 hr with 5 μM MG132. Biochemical cell fractionation, immunoprecipitation, and GST pull-downs are described in the Supplemental Experimental Procedures. Rac activity assays were performed as previously described (Mack et al., 2012).

### Ubiquitylation Experiments

In vivo and in vitro detection of protein ubiquitylation were performed as previously described (Choo and Zhang, 2009) with further details provided in the Supplemental Experimental Procedures.

### Cell Growth

Cell growth was assessed using the Sulforhodamine B assay as previously described (Vichai and Kirtikara, 2006).

### Immunofluorescence Microscopy

Cells were grown on glass coverslips and fixed with 100% ice-cold methanol or 3.7% formaldehyde. Postfixation, cells were washed with PBS and blocked with 3% BSA in 0.1% Triton/PBS for 1 hr before antibodies were added.

### Halo Pulse-Chase Analysis

Halo pulse-chase analysis was carried out as previously described (Yamaguchi et al., 2009) with further details provided in the Supplemental Experimental Procedures.

### Duolink PLA

Duolink PLA was performed using the Duolink II Red or Green Starter Kits (Sigma) following the manufacturer’s instructions and is described in further detail in Supplemental Experimental Procedures.

### Transwell Migration and Invasion Assays

Transwell migration and invasion experiments were performed as previously described (Marshall, 2011). Modifications are detailed in the Supplemental Experimental Procedures.

### Xenograft Assays

Xenograft assay was performed as previously described (Chapman et al., 2014). Further details are provided in the Supplemental Experimental Procedures. Approval for this procedure was given by The University of Manchester Ethical Review Board and performed according to UK Home Office Regulations.

### Immunohistochemical Analysis

The squamous cell tissue microarray included multiple tumor specimens from 83 patients. Immunopositivity for HUWE1, TIAM1, and c-MET protein expression in the TMAs was evaluated independently by two investigators (D.N. and L.V.) as described in Supplemental Experimental Procedures. Ethical approval for these studies was conferred under the MCRC Biobank Research Tissue Bank Ethics (07/H1003/161+5) from NRES Committee North West, Greater Manchester South Ethical.

### Statistical Analysis

The specific statistical tests used are indicated in the figure legends alongside the p values and were carried out using GraphPad Prism version 6.0.

## Author Contributitions

L.V. performed the majority of experiments, data analysis, and manuscript preparation. C.-T.T. generated preliminary data, identified the ubiquitylation site together with D.S., and generated Figures S1C, S3A, S3C, S3E, and S5B. A.C. of A.F.L.H.’s laboratory designed and performed the zebrafish experiments. D.N. is the pathologist who scored the TMA together with L.V. N.A.M. found and confirmed the interaction between TIAM1 and HUWE1. D.S. performed the MS analysis. R.B. was responsible for the construction of the lung TMA used in the study. A.M. was the grant holder and principal investigator who supervised the study and manuscript preparation and made intellectual contributions throughout.

## Supplemental Information

Document S1. Supplemental Experimental Procedures and Figures S1–S7

Document S2. Article plus Supplemental Information
