# Supplementary material for: HUWE1 Ubiquitylates and Degrades the RAC Activator TIAM1 Promoting Cell-Cell Adhesion Disassembly, Migration, and Invasion
Source: Cell Rep. 2014 Dec 24;10(1):88–102. doi: 10.1016/j.celrep.2014.12.012 (PMC4542307; doi:10.1016/j.celrep.2014.12.012)
Supplement: Document S1. Supplemental Experimental Procedures and Figures S1–S7 [file mmc1.pdf]

Cell Reports

Supplemental Information

HUWE1 Ubiquitylates and Degrades the Rac Activator TIAM1 Promoting Cell-Cell Adhesion Disassembly, Migration, and Invasion

Lynsey Vaughan, Chong-Teik Tan, Anna Chapman, Daisuke Nonaka, Natalie Mack, Duncan Smith, Richard Booton, Adam F.L. Hurlstone, and Angeliki Malliri

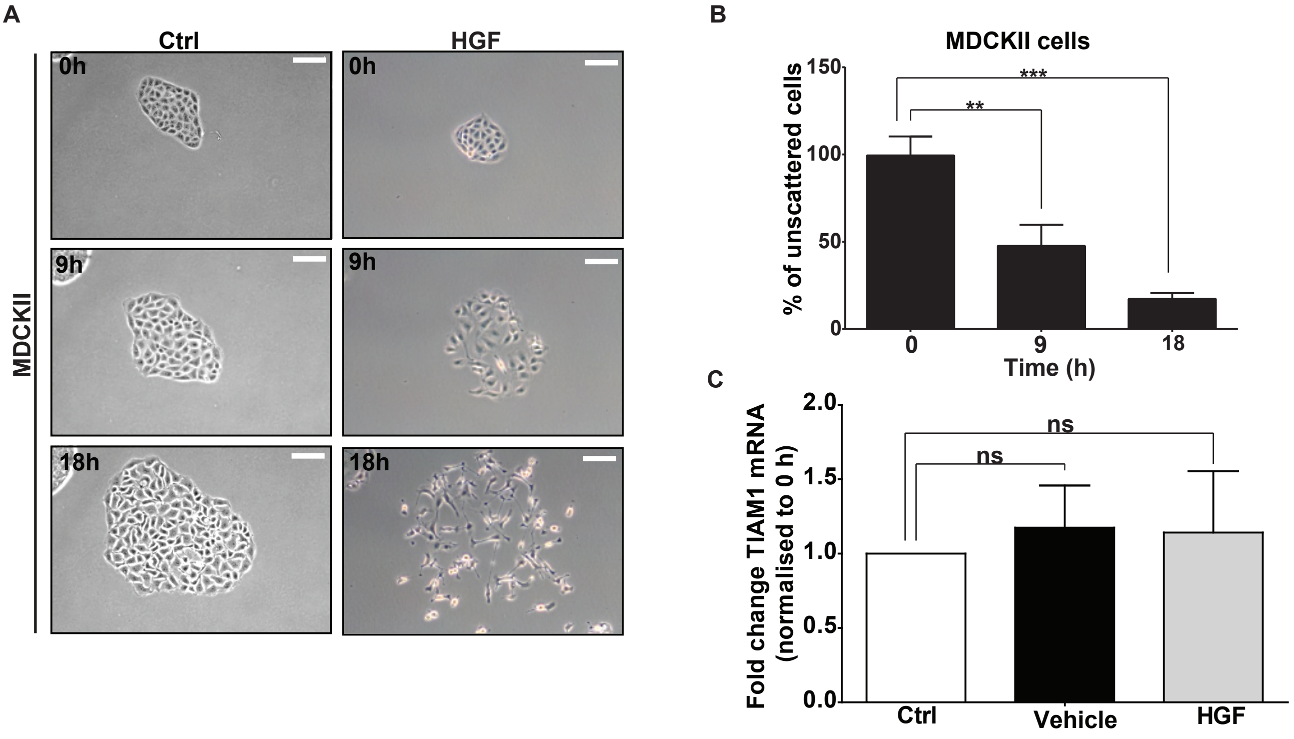
A
Ctrl
HGF
MDCKII
0h
0h
9h
9h
18h
18h
B
MDCKII cells
% of unscattered cells
150
100
50
0
***
**
0
9
18
Time (h)
C
Fold change TIAM1 mRNA (normalised to 0 h)
2.0
1.5
1.0
0.5
0.0
ns
ns
Ctrl
Vehicle
HGF

Figure S1.

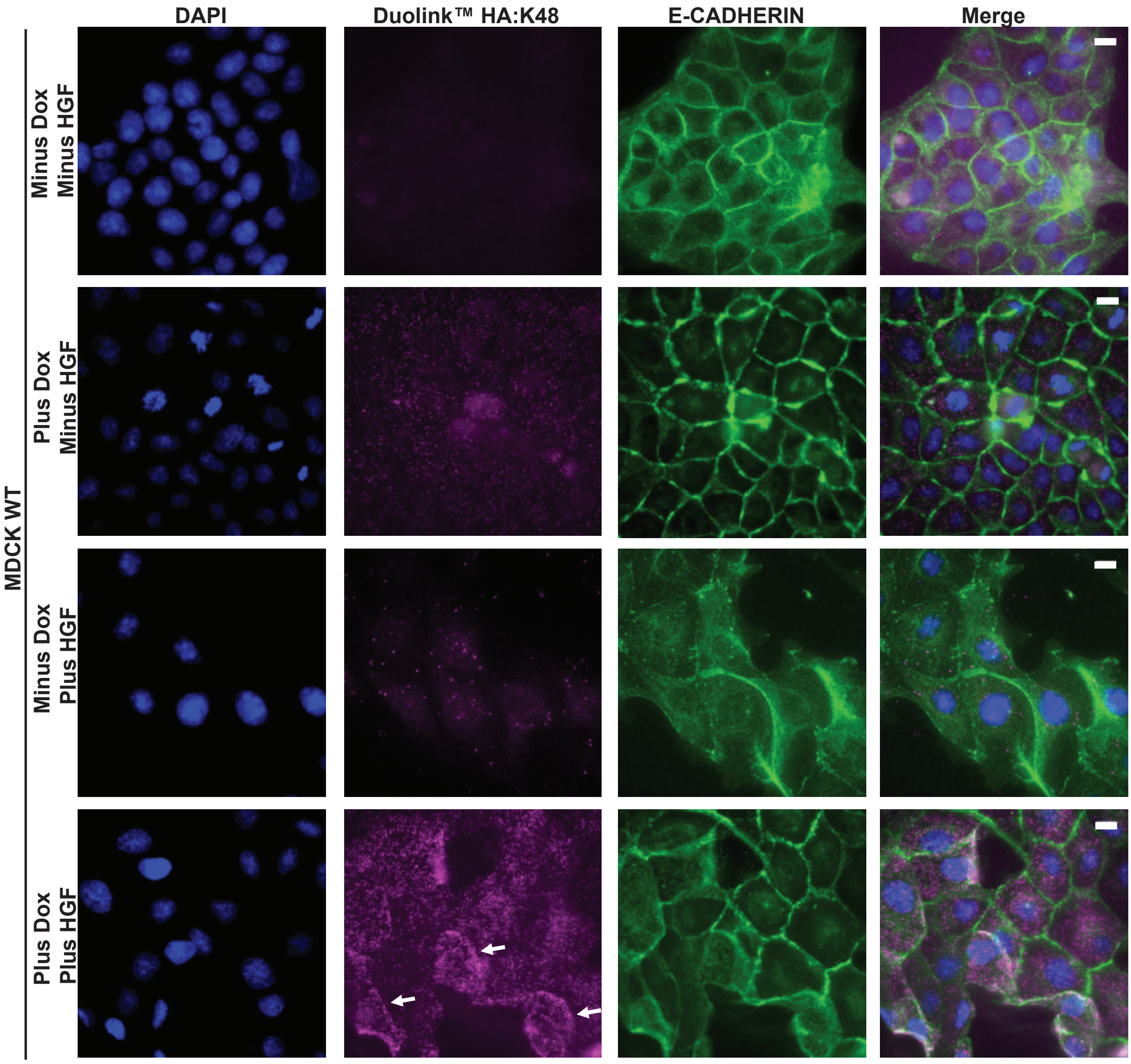

DAPI
Duolink™ HA:K48
E-CADHERIN
Merge
MDCK WT
Minus Dox
Minus HGF
Plus Dox
Minus HGF
Minus Dox
Plus HGF
Plus Dox
Plus HGF

Figure S2.

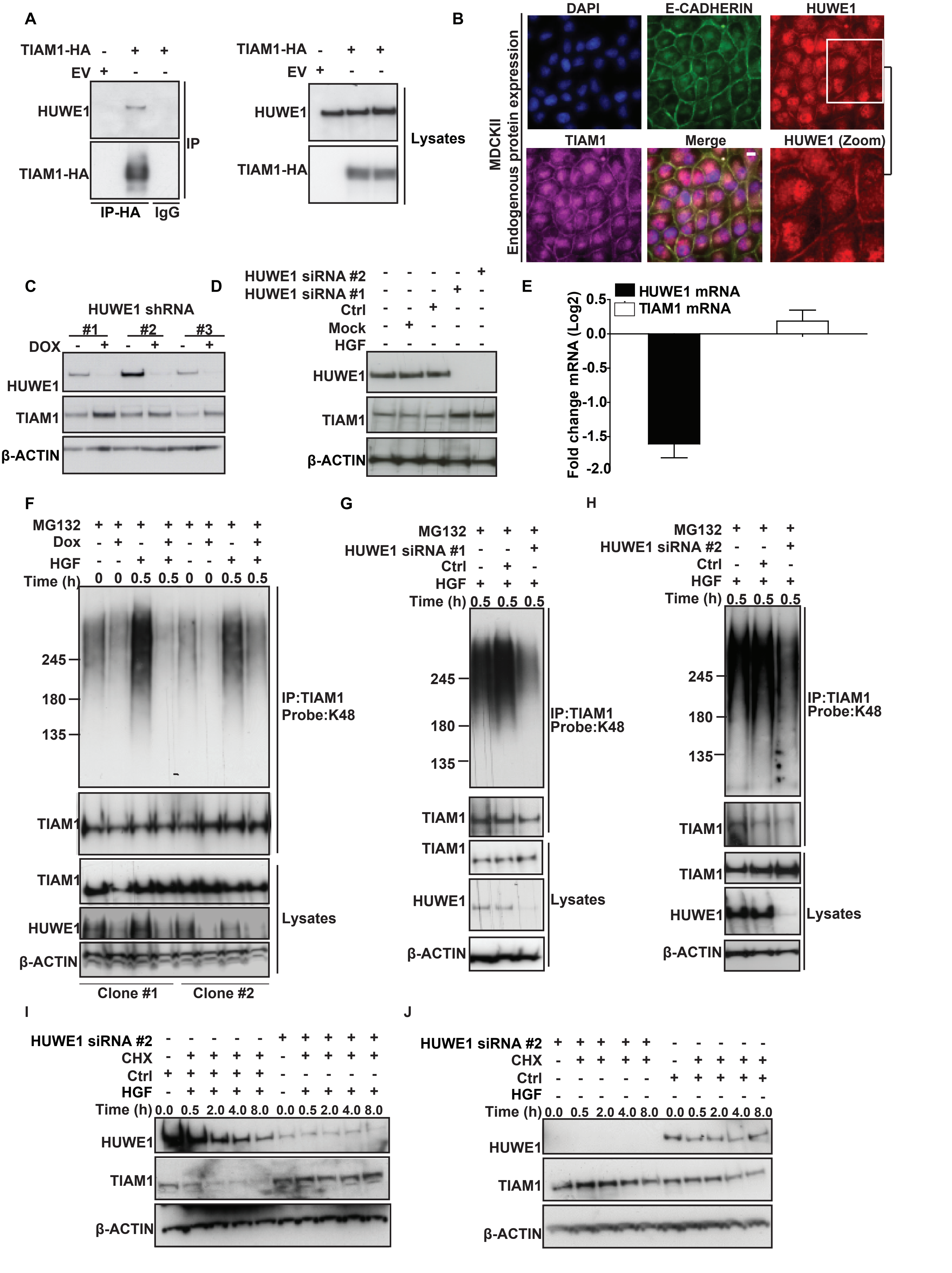
A
TIAM1-HA - + +
EV + - -
HUWE1
TIAM1-HA
IP
IP-HA
IgG
Lysates
B
DAPI
E-CADHERIN
HUWE1
TIAM1
Merge
HUWE1 (Zoom)
MDCKII
Endogenous protein expression
C
HUWE1 shRNA
#1
#2
#3
DOX
β-ACTIN
D
HUWE1 siRNA #2
HUWE1 siRNA #1
Ctrl
Mock
HGF
E
HUWE1 mRNA
TIAM1 mRNA
Fold change mRNA (Log2)
F
MG132
Dox
HGF
Time (h)
IP:TIAM1
Probe:K48
Clone #1
Clone #2
G
H
I
CHX
J

Figure S3.

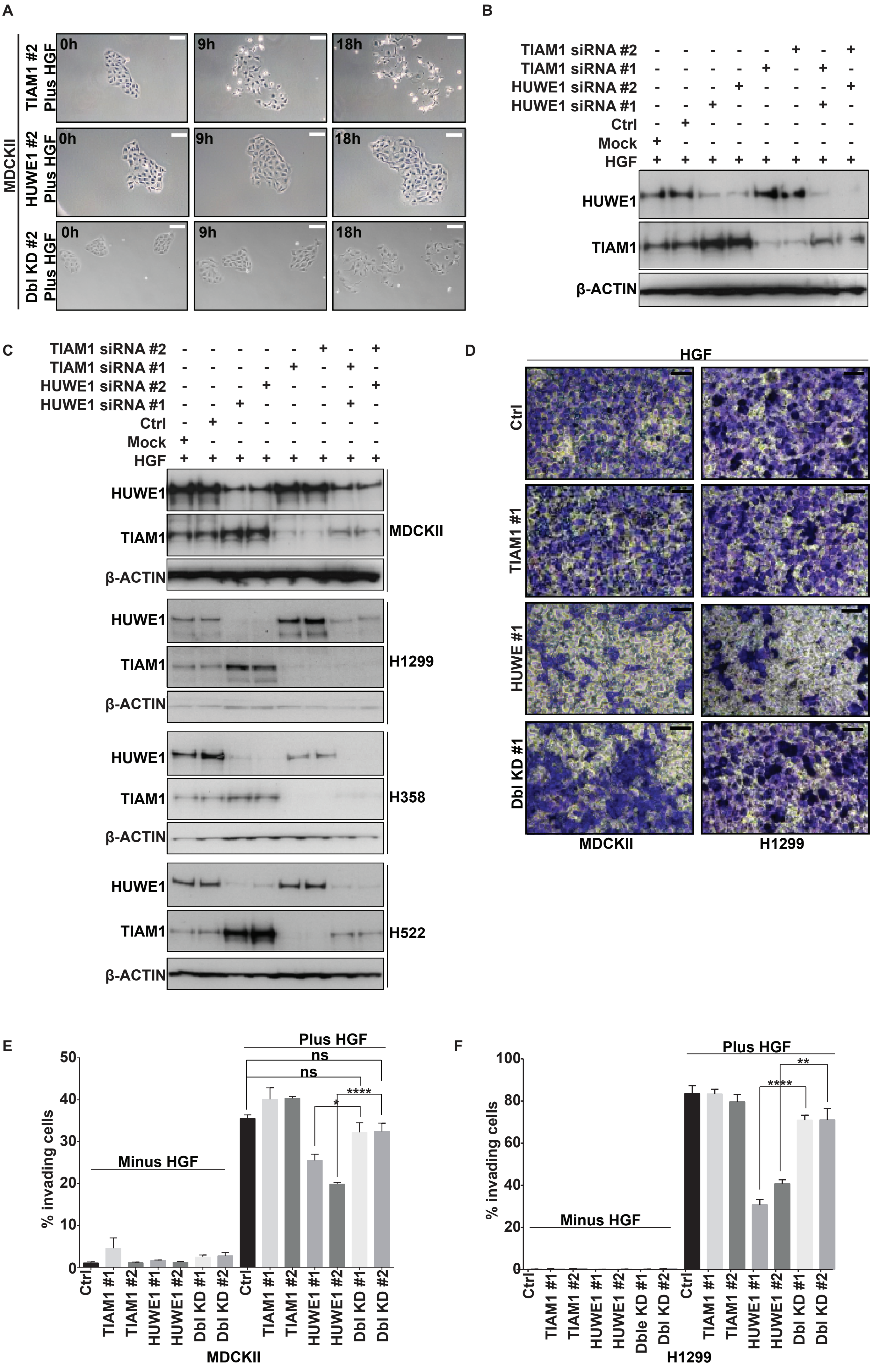
A
MDCKII
TIAM1 #2 Plus HGF
HUWE1 #2 Plus HGF
Dbl KD #2 Plus HGF
0h
9h
18h
B
TIAM1 siRNA #2
TIAM1 siRNA #1
HUWE1 siRNA #2
HUWE1 siRNA #1
Ctrl
Mock
HGF
HUWE1
TIAM1
β-ACTIN
C
MDCKII
H1299
H358
H522
D
HGF
Ctrl
TIAM1 #1
HUWE #1
Dbl KD #1
MDCKII
H1299
E
Plus HGF
Minus HGF
ns
*
****
% invading cells
Ctrl
TIAM1 #1
TIAM1 #2
HUWE1 #1
HUWE1 #2
Dbl KD #1
Dbl KD #2
MDCKII
F
Plus HGF
Minus HGF
****
**
% invading cells
Dble KD #1
H1299

Figure S4.

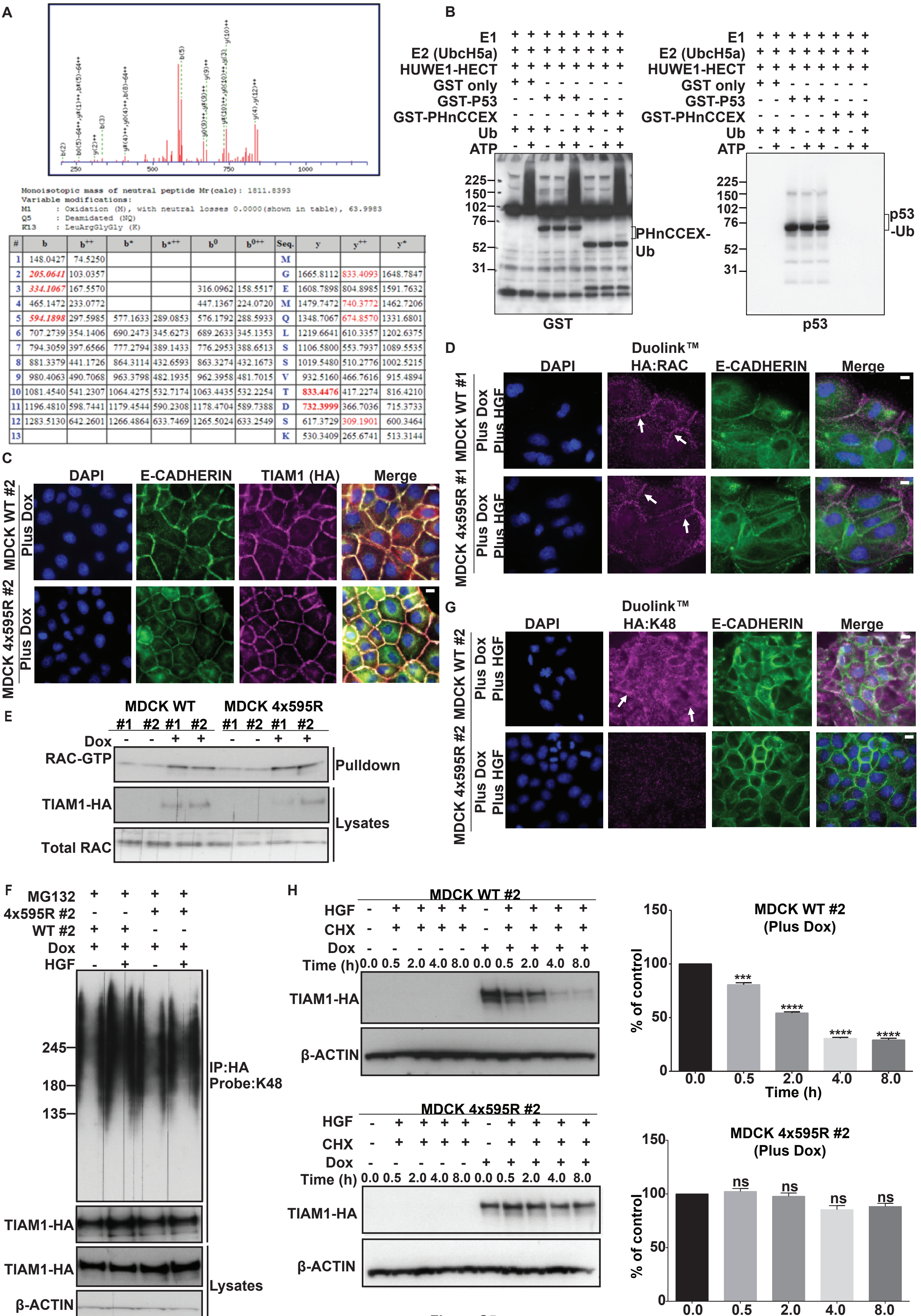

#	b	b++	b*	b*++	b0	b0++	Seq.	y	y++	y*
1	148.0427	74.5250					M			
2	205.0641	103.0357					G	1665.8112	833.4093	1648.7847
3	334.1067	167.5570			316.0962	158.5517	E	1608.7898	804.8985	1591.7632
4	465.1472	233.0772			447.1367	224.0720	M	1479.7472	740.3772	1462.7206
5	594.1898	297.5985	577.1633	289.0853	576.1792	288.5933	Q	1348.7067	674.8570	1331.6801
6	707.2739	354.1406	690.2473	345.6273	689.2633	345.1353	L	1219.6641	610.3357	1202.6375
7	794.3059	397.6566	777.2794	389.1433	776.2953	388.6513	S	1106.5800	553.7937	1089.5535
8	881.3379	441.1726	864.3114	432.6593	863.3274	432.1673	S	1019.5480	510.2776	1002.5215
9	980.4063	490.7068	963.3798	482.1935	962.3958	481.7015	V	932.5160	466.7616	915.4894
10	1081.4540	541.2307	1064.4275	532.7174	1063.4435	532.2254	T	833.4476	417.2274	816.4210
11	1196.4810	598.7441	1179.4544	590.2308	1178.4704	589.7388	D	732.3999	366.7036	715.3733
12	1283.5130	642.2601	1266.4864	633.7469	1265.5024	633.2549	S	617.3729	309.1901	600.3464
13							K	530.3409	265.6741	513.3144

Figure S5.

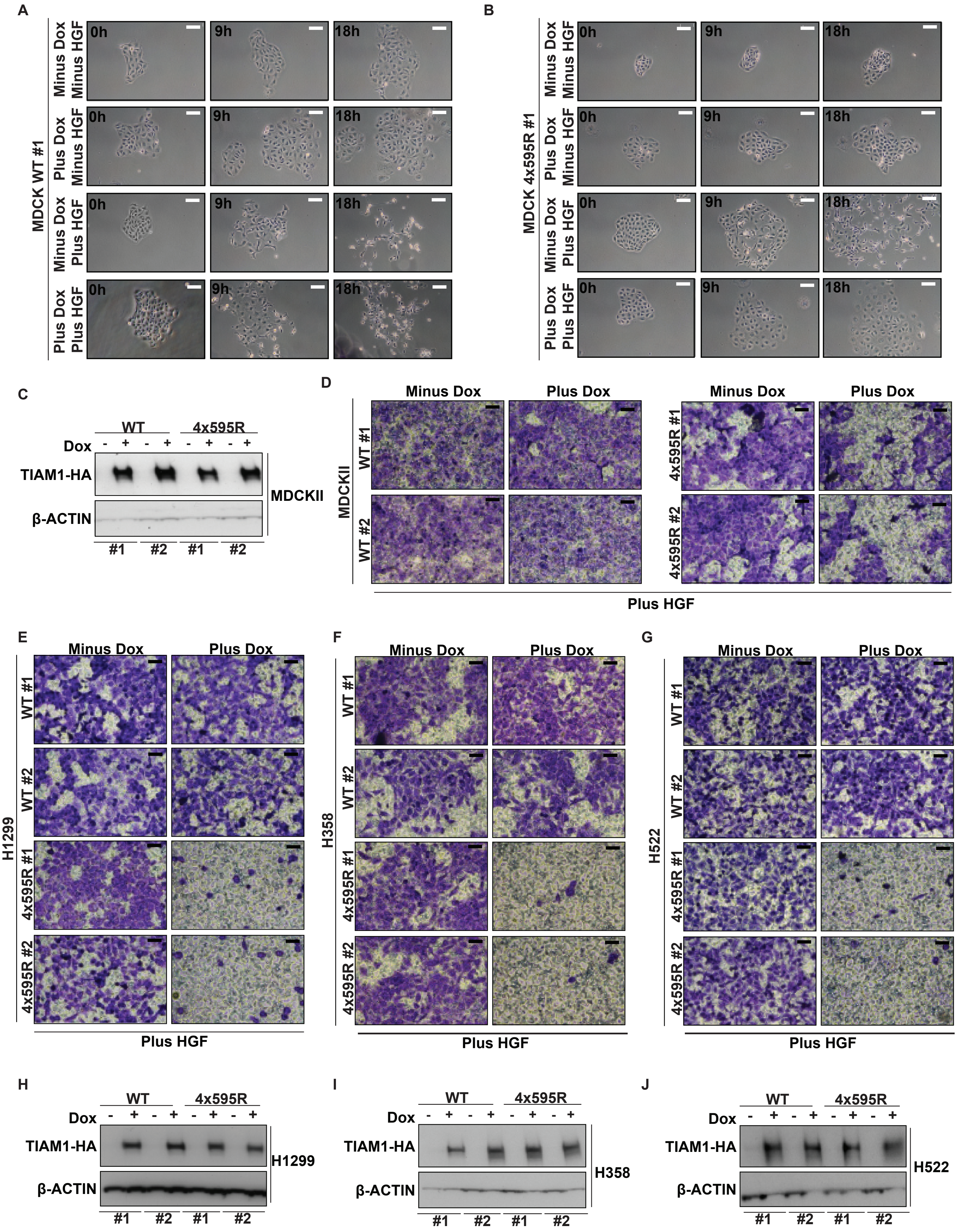
A
MDCK WT #1
Minus Dox Minus HGF
Plus Dox Minus HGF
Minus Dox Plus HGF
Plus Dox Plus HGF
0h
9h
18h
B
MDCK 4x595R #1
C
WT
4x595R
Dox
TIAM1-HA
β-ACTIN
MDCKII
#1
#2
D
Minus Dox
Plus Dox
MDCKII
WT #1
WT #2
4x595R #1
4x595R #2
Plus HGF
E
H1299
F
H358
G
H522
H
I
J

Figure S6.

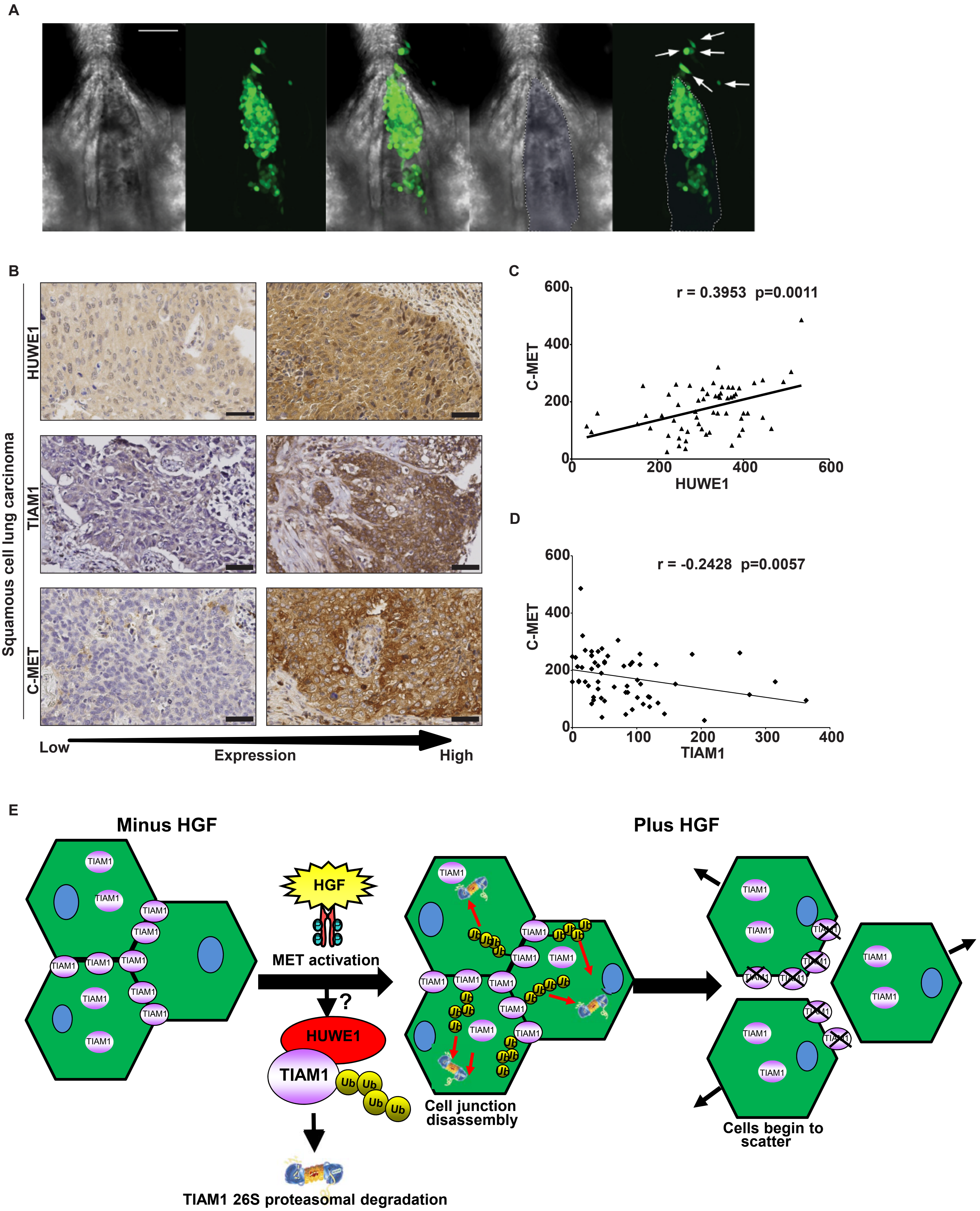
A
B
Squamous cell lung carcinoma
HUWE1
TIAM1
C-MET
Low
Expression
High
C
r = 0.3953 p=0.0011
C-MET
HUWE1
600
400
200
0
0
200
400
600
D
r = -0.2428 p=0.0057
C-MET
TIAM1
600
400
200
0
0
100
200
300
400
E
Minus HGF
Plus HGF
HGF
MET activation
?
HUWE1
TIAM1
Ub
TIAM1 26S proteasomal degradation
Cell junction disassembly
Cells begin to scatter

Figure S7.

SUPPLEMENTAL FIGURE LEGENDS

Figure S1, related to Figure 1. HGF stimulation induces scattering of epithelial MDCKII cells. (A) MDCKII cells were treated in the presence (HGF) or absence (Ctrl) of 10 ng/ml HGF for the indicated times and resultant cell scattering monitored by phase contrast microscopy. Scale bar = 50 µm. (B) Quantification of (A) from three independent experiments is shown. Results are mean values ± SE. ** signifies $p<0.001$ and *** $p<0.0005$ (Unpaired t test). (C) MDCKII cells were treated with 10 ng/ml HGF (HGF), 0.1% BSA (vehicle) or left untreated (Ctrl) for 6 h and relative TIAM1 mRNA levels determined by quantitative RT-PCR. Data were normalised to the hypoxanthine phosphoribosyl transferase 1 (HPRT1) housekeeping gene and were obtained from three biological replicates. Error bars indicate ± SD, ns = not significant.

Figure S2, related to Figure 2. HGF stimulation induces ubiquitylation of TIAM1. MDCKII cells inducibly expressing HA-tagged TIAM1 WT were left untreated (Minus Dox, Minus HGF), pre-treated with 1 µg/ml Doxycycline (Dox) for 24 h to induce exogenous TIAM1-HA expression (Plus Dox, Minus HGF), treated with 10 ng/ml HGF alone (Minus Dox, Plus HGF) or with both Dox and HGF (Plus Dox, Plus HGF) and fixed. The Duolink protocol was then performed to monitor the extent and localisation of K48 ubiquitylated TIAM1. White arrows indicate intercellular junctions. Scale bar = 10 µm.

Figure S3, related to Figure 3. TIAM1 is ubiquitylated by HUWE1. (A) HEK293T cells were transfected with an empty vector (EV, pCDNA3-HA) or an expression vector containing HA-tagged wild-type TIAM1 (TIAM1-HA). 24 hours post-transfection, protein extracts were immunoprecipitated with a mouse HA-specific antibody or non-specific mouse IgG as a negative control and lysates probed for endogenous HUWE1. (B) MDCKII cells were stained by immunofluorescence for HUWE1, E-CADHERIN, TIAM1 and DAPI. Panels show representative images from one of three independent experiments. Scale bar = 10 µm. (C) MDCKII single cell clones were generated allowing inducible knockdown of HUWE1 (shRNA clones #1 - #3). Representative immunoblots of HUWE1 knockdown and TIAM1 protein levels are shown. (D) MDCKII cells were transfected with 2 different siRNAs (#1 and #2) or a non-targeting control siRNA (Ctrl). Cells were lysed, protein extracted and subsequently immunoblotted for TIAM1 and HUWE1 96 h post transfection. (E) HUWE1 was depleted in MDCKII

cells using shRNA and relative TIAM1 and HUWE1 mRNA expression levels determined by quantitative RT-PCR. Data were normalised to the house-keeping gene HPRT1. Results are mean values ± SD from four independent experiments. (F) Endogenous HUWE1 was inducibly depleted in 2 different HUWE1 shRNA knockdown clones and at 48 h post knockdown cells were pre-treated with 5 µM MG132 for 3 h and 10 ng/ml HGF for 0.5 h as indicated and protein harvested. TIAM1 was immunoprecipitated under denaturing conditions with a TIAM1 antibody, and immunoprecipitates probed for K48-linked ubiquitin using a K48-linkage specific antibody. (G and H) MDCKII cells were transfected with 2 different siRNAs (#1 and #2) or a non-targeting control siRNA (Ctrl). At 96 h post knockdown cells were pre-treated (3 h) with 5 µM MG132 and 10 ng/ml HGF for 0.5 h. Protein was then harvested and TIAM1 was immunoprecipitated under denaturing conditions with a TIAM1 antibody, and immunoprecipitates probed for K48-linked ubiquitin using a K48-linkage specific antibody. (I and J) MDCKII cells were transfected with HUWE1 siRNA #2 or non-targeting siRNA (Ctrl) as indicated. Cycloheximide (CHX, 50 µg/ml) was added to cells 96 h post transfection where indicated and cells were treated with HGF for the indicated times (I) or left untreated (J). Western blot analysis was performed to analyse amounts of TIAM1 and HUWE1. In C, D and F-J β-ACTIN was used as loading control.

Figure S4, related to Figure 4. HUWE1 mediated TIAM1 degradation controls HGF-induced motility and invasion. (A) MDCKII cells were transfected with siRNA targeting TIAM1 or HUWE1 alone or in combination. At 96 h post transfection cells were stimulated with 20 ng/ml HGF for 18 h and resultant cell scattering was monitored by phase contrast microscopy. Representative images are shown. Scale bar = 50 µm. (B) Western blot analysis of HUWE1 and TIAM1 expression of cells used in Figure 4A and S4A. (C) Western blot analysis of HUWE1 and TIAM1 expression of cells used in Figure 4C-4G. β-ACTIN was used as a loading control in (B) and (C). (D) MDCKII and H1299 cells were transfected with non-targeting siRNA (Ctrl), or siRNA targeting TIAM1 or HUWE1 alone or in combination and seeded in a modified Boyden chamber (where inserts had been coated with 100 µl layer of 500 µg/ml collagen) to assay for invasion in the presence (HGF) of 10 ng/ml HGF. After 5 days, invading cells were stained with Crystal Violet. Panels show representative images from one of at least three independent experiments. Scale bar = 150 µm. (E-F) Crystal Violet was eluted, absorbance measured at 600 nm and relative invasion determined for (E) MDCKII and (F) H1299

cells by plotting optical density against a standard curve of the appropriate cell type previously prepared and normalising this to total cell number for each condition. Results are mean values ± SE. * signifies $p<0.05$, ** $p<0.001$ 5 and **** $p<0.0001$, ns = not significant (Unpaired t test).

Figure S5, related to Figure 5. TIAM1 is ubiquitylated on lysine residue 595. (A) Composite MS/MS spectrum and fragment ion matrix for MGEMQLSSVTDSK (LeuArgGlyGly) supporting the site of TIAM1 ubiquitylation as K595. The peptide sequence and the absence of the LeuArgGlyGly modification N terminal to K13 are confirmed by a b ion series including b2, b3, & b5. The peptide sequence and localisation of the site of Gly Gly modification as K13 is confirmed by a y/y^{++}ion series including y3, y4, $y2^{++}$, $y9^{++}$,$y10^{++}$, & $y12^{++}$. (B) *In vitro* ubiquitylation assays were performed using E1 activating enzyme (UBE1/Uba1), E2 conjugating enzyme (UBE2D1/UbcH5a), E3 ubiquitin ligase (HUWE1-HECT domain), GST-tagged p53 (positive control), TIAM1 PHnCCEx (tagged with GST-His dual tags), GST-only, ubiquitin (Ub) and ATP. ATP or ubiquitin were omitted from the negative controls. Brackets indicate the ubiquitylated isoforms of TIAM1 and p53. Following the *in vitro* reactions protein samples were immunoblotted for either GST (left panel) or p53 (right panel). (C) MDCKII cells inducibly expressing (Plus Dox) either WT TIAM1 (WT #2) or mutant 4x595R TIAM1 (4x595R #2) were seeded on glass coverslips for 48 h. Cells were then fixed and stained by immunofluorescence for E-CADHERIN, TIAM1-HA and DAPI. Panels show representative images from MDCK WT #2 and MDCK 4x595R #2 clones. Scale bar = 10 µm. (D) MDCKII cells inducibly expressing (Plus Dox) either WT TIAM1 (WT #1) or mutant 4x595R TIAM1 (4x595R #1) were seeded on glass coverslips for 48 h, treated with 10 ng/ml HGF for 0.5 h and fixed. The Duolink protocol was then performed to monitor the extent and co-localisation of RAC1 with TIAM1-HA. White arrows indicate co-localisation at intercellular junctions. Scale bar = 10µm. (E) MDCKII cells inducibly expressing (+ Dox) either WT TIAM1 (WT #1 and #2) or mutant 4x595R TIAM1 (4x595R #1 and #2) were lysed and assayed for RAC activity. (F) MDCKII cells inducibly expressing (+ Dox) either WT TIAM1 (WT #2) or mutant 4x595R TIAM1 (4x595R #2) were treated with 5 µM MG132 for 3 h and either left untreated or treated with 10 ng/ml HGF and protein harvested 0.5 h post stimulation. HA-tagged TIAM1 was immunoprecipitated under denaturing conditions with an HA antibody and probed for K48-linked ubiquitin using a K48-linkage specific antibody. (G) MDCKII cells inducibly expressing (Plus Dox) either exogenous HA-tagged WT TIAM1 or mutant 4x595R TIAM1 were treated for 3 h

with 5 µM MG132 and with 20 ng/ml HGF for 1 h, fixed and the Duolink protocol performed to monitor the extent and localisation of K48-linked ubiquitylated TIAM1. White arrows indicate intercellular junctions. Scale bar = 10 µm. (H) MDCKII cells inducibly expressing (+ Dox) either WT TIAM1 (WT #2) or mutant 4x595R TIAM1 (4x595R #2) were treated with 50 µg/ml cycloheximide (CHX) as indicated and HGF (10 ng/ml) for the indicated times, after which protein was extracted. Lysates were immunoblotted for TIAM1-HA expression. β-ACTIN was used as a loading control in F and H. Results are mean values ± SE. *** signifies $p<0.005$ and **** $p<0.0001$, ns = not significant (Unpaired t test).

Figure S6, related to Figure 6. Ubiquitylation of TIAM1 at lysine residue 595 regulates cell migration and invasion. (A-B) MDCKII cells inducibly expressing (Plus Dox) either TIAM1 WT (WT #1) (A) or TIAM1 4x595R (4x595R #1) (B) were stimulated with 20 ng/ml HGF as indicated (Plus HGF). Resultant cell scattering was monitored by phase contrast microscopy. Scale bar = 50 µm. (C) Representative western blot analysis of WT TIAM1 and 4x595R mutant TIAM1 induction in cells used in Figure 6C and 6D and (A) and (B) above. (D) MDCKII, (E) H1299**,** (F) H358 and (G) H522 cells inducibly expressing (Plus Dox) either TIAM1 WT (WT #1 and WT #2) or TIAM1 4x595R (4x595R #1 and 4x595R #2) were seeded in a modified Boyden chamber coated with collagen and left to invade for 1 day in the presence of 10 ng/ml HGF. Invading cells were fixed in 100% methanol and stained with 0.4% Crystal Violet. Panels show representative images from one of at least three independent experiments. Scale bar = 150 µm. (H-J) Representative western blot analysis of WT TIAM1 and 4x595R mutant TIAM1 induction in cells used in Figure 6F-6H and E-G above.

Figure S7, related to Figure 7. HUWE1 mediated TIAM1 degradation controls lung cancer cell invasion *in vivo*. HUWE1 and TIAM1 protein expression are negatively correlated in stage I and stage II squamous cell lung carcinoma. (A) Green fluorescent H1299 cells injected into the pericardial cavity of a Zebrafish embryo. Engrafted cells were imaged at 4 days post injection (dpi). Dotted line in fourth panel indicates the pericardial cavity of the Zebrafish embryo and white arrows in the fifth panel invading cells. Scale bar = 100 μm. (B) Representative immunohistochemistry images of lung squamous cell carcinomas expressing low or high HUWE1, TIAM1 and C-MET protein. Scale bar = 50 µm. (C) Scatter plot depicting the statistically significant Spearman's positive correlation between HUWE1 and C-MET in corresponding tissue sections. (D) Scatter plot depicting the

statistically significant Spearman's negative correlation between C-MET and TIAM1 in corresponding tissue sections. (E) Model describing the HGF-induced ubiquitylation and degradation of TIAM1 by HUWE1 predominantly at cell–cell adhesions.

SUPPLEMENTAL EXPERIMENTAL PROCEDURES

Constructs. cDNA encoding full-length mouse TIAM1 (GenBank accession NM_009384) carrying a C-terminal HA tag in pCDNA3 expression vector has previously been described (Mack et al., 2012; Michiels et al., 1997; Woodcock et al., 2009). pET-TIAM1 PHCCEx-His-GST was a kind gift from T. Hakoshima (Nara Institute of Science and Technology) and has been previously described (Terawaki et al., 2010). pRetroXT-TIAM1-HA (puro) was cloned by N. Mack (Mack et al., 2012). HA-EGFP dual tags version of this construct was made by subcloning the EGFP tag fused to TIAM1 in pEFGP-TIAM1-HA into pRetro-XT-TIAM1-HA (puro). pRetro-XT-TIAM1-Halo (puro) was made by insertion of the Halo tag amplified from pFC14A-Halo (Promega). pFastBac-TIAM1-HA-His-CTOPO was cloned by sub-cloning full-length Tiam1 into pFastBac-His-CTOPO expression vector (Invitrogen). pCDNA3.1(+)-myc-HisA, pcDNA3, pCDNA3-HA and pCDNA4-TO were purchased from Invitrogen. pGEX-GST-HUWE1-HECT (GST-ARF-BP1 3760-4374) was provided by W. Gu (Columbia University) and has been previously described (Chen et al., 2005) and pSuperior.si5635(puro) was a kind gift from W. Xiaodong and has been previously described (Zhong et al., 2005).

Antibodies. All antibodies used are detailed below.

Primary Antibody	*Species*	*Supplier*
ACTIN	Mouse	Sigma-Aldrich
CAVEOLIN 1	Mouse	BD Biosciences
E-CADHERIN	Rat	Abcam
ERK	Rabbit	Cell Signaling Technology
RAC	Mouse	BD Biosciences
HA-tag (12CA5)	Mouse	Roche
HA-tag	Rabbit	Abcam
HUWE1	Rabbit	Bethyl Lab.
MEK1/2	Rabbit	Cell signalling
Phospho-ERK (Thr202/Tyr204)	Mouse	Cell Signaling Technology
TIAM1	Rabbit	Bethyl Lab.
TIAM1	Sheep	R&D System
Ubiquitin (P4D1)	Mouse	Enzo Lifesciences
Ubiquitin (K48)	Rabbit	Enzo Lifesciences

Secondary antibodies utilized for immunofluorescence were as follows: IgG peroxidase-conjugated (GE Healthcare); Alexa Fluor 488, 568 and 647 (Molecular Probes).

Cell culture. MDCKII and HEK293T cells were maintained in DMEM (Sigma) with 10% FBS (GIBCO) or 10% tetracycline-free FBS (Autogen Bioclear) and supplemented with the appropriate antibiotics (G418, 1 mg/ml; puromycin, 2 µg/ml; blasticidin 1 µg/ml, all from Sigma). The H1299, H358, H522 and H596 lung carcinoma cell lines were all maintained in RPMI (Sigma) with 4mM Glutamine and tetracycline-free 10% FBS. Antibiotic selection was withheld during the experiments.

Generation of cell lines. Retroviral transduction was performed as previously described (Woodcock et al., 2009) and transfections were performed with TransIT-LT1 (Mirus) according to the Manufacturer's instructions followed by appropriate antibiotic selection. For inducible expression, cells were treated with doxycycline (100 ng/ml for confluent cells, 50 ng/ml for sub confluent cells) for at least one day before experimental analysis.

Transient siRNA silencing. Transient silencing of HUWE1 or TIAM1 was achieved by transfection of siRNA oligos from Eurofins MWG operon using RNAiMAX (Invitrogen) following the manufacturer's instructions. Cells were subjected to 'reverse' (cells in suspension) transfection on day 1 and processed and analysed 48-96 h after transfection as indicated in each individual experiment.

Three different siRNA oligos were used to target HUWE1:

Huwe1#1 5'-GAGUUUGGAGUUUGUGAAGUU-3'

Huwe1 #2 5'- UGCCGCAAUCCAGACAUAU-3'

Huwe1 #3 5-AAUUGCUAUGUCUCUGGGACA-3'

Three different siRNA oligos were used to target TIAM1:

Tiam1 #1 5'-GAGGUUGCAGAUCUGAGCA-3'

Tiam1 #2 5'-5'-AGAGCGCACCUACGUGAAA-3'

Tiam1 #3 5'-GCUUGAGACCUGUGUCUUA-3'

In all of the reported assays, the targeting oligos yielded comparable results and for each experiment a non-targeting oligo control was used (Dharmacon – non-targeting siRNA).

Biochemical fractionation. Fractionation was carried out according to a previously described protocol (Holden and Horton, 2009). Briefly cells treated as indicated were permeabilised on ice using digitonin lysis buffer (50mM Tris-HCl pH 7.5, 150mM NaCl, 1mM EDTA and 0.01% digitonin) for 5

min. After a brief centrifugation the cytoplasmic fraction was removed. The insoluble pellet was resuspended in NP40 lysis buffer (150mM NaCl, 50mM HEPES pH 7.4 and 1% NP40) and incubated on ice for a further 30 min. After centrifugation the membrane fraction was removed. Both buffers contained a protease inhibitor cocktail (Sigma), phosphatase inhibitor cocktails 1 and 2 (Sigma) and 5mM sodium vanadate (Sigma). Western blotting was then performed.

Immunoprecipitation. Protein G beads were pre-blocked with an equal volume of 5%BSA/PBS to make a 50% Protein G slurry and lysates pre-cleared using 50% Protein G beads. Cells were treated as indicated in figure legends and lysed in immunoprecipitation lysis buffer (50mM Tris-HCl pH 7.5, 150mM NaCl, 1% (v/v) Triton-X-100, 10% (v/v) glycerol, 2mM EDTA, 25mM NaF and 2mM NaH_2PO4) containing protease and phosphatase inhibitor cocktails (Sigma). Lysates were then incubated with beads conjugated to the appropriate antibody for 3 h at 4ºC with gentle mixing. Beads were then washed 3 times and eluted with 30 µl of 2X SDS sample buffer. Western blotting was then performed.

***In vivo* ubiquitylation experiments.** Detection of protein ubiquitylation in cultured cells was performed using the method described previously (Choo and Zhang, 2009). Briefly either control cells (to detect endogenous TIAM1 ubiquitylation) or cells expressing HA-tagged TIAM1 WT or TIAM1 4x595R (to detect exogenous TIAM1 ubiquitylation) were washed in ice-cold PBS, lysed in 2% SDS denaturing lysis buffer and boiled for 10 min. DNA was then sheared by sonication and samples diluted in dilution buffer (Choo and Zhang, 2009). Samples were mixed for 30 min at 4ºC and spun at 20000 g for 30 min. Protein concentrations were then measured and lysates incubated overnight at 4ºC with beads pre-coated in the antibody of choice (indicated in the individual figure legend). Post incubation beads were spun down at 5000 g for 5 min and washed twice with wash buffer. The remaining resin was then boiled with 2X SDS loading buffer and samples loaded onto an SDS-PAGE gel for immunoblotting.

***In vitro* ubiquitylation experiments.** *In vitro* ubiquitylation assay was performed as previously described (Choo and Zhang, 2009) with some modifications. Briefly, the ubiquitylation reaction was reconstituted using 10 ng of E1 activating enzyme (UBE1/Uba1) (Ubiquigent), 100 ng of E2 conjugating enzyme (UBE2D1/UbcH5a) (Ubiquigent), 400 ng of E3 ubiquitin ligase (HUWE1 HECT

only), 20 ng of GST-tagged p53 (Millipore), 20 ng of PHnCCEX-TIAM1 tagged with GST-His or GST-only, 5 µg of bovine ubiquitin (Sigma-Aldrich), 3 mM ATP in a reaction buffer containing 50 mM Tris, 5 mM $MgCl_2$ and 2 mM DTT at pH7.5. HUWE1 HECT only, PHnCCEX-TIAM1 and GST only were expressed in Rosetta bacteria and purified by GST-pull down as described below. The reaction was incubated at 30°C for 2 hours and subjected to western blot analysis.

GST-pull down. Bacterial cultures expressing HUWE1 HECT domain, PHnCCEX-TIAM1 or GST-only were centrifuged, bacterial pellets washed in PBS and re-pelleted. The pellets were then lysed and sonicated in PBS-based GST lysis buffer containing 0.5 M NaCl, 1 mM EDTA and complete protease inhibitor cocktail (Roche). Lysates were incubated with Glutathione Sepharose beads (GE healthcare) for 2 h and beads washed in GST lysis buffer. Protein was eluted with 10 mM Glutathione (Sigma-Aldrich), 40 mM Tris-HCl pH 8.0 and dialysed overnight into buffer A containing 50 mM Tris-HCl pH 8.0, 0.5 mM $MgCl_2$ and 1 mM β-mercaptoethanol using a Dialysis cassette (Thermo-scientific).

Mass spectrometry analysis. TIAM1 was immunoprecipitated from HEK293T cells as detailed above and after SDS-PAGE the gel was stained using Coomasie (SimplyBlueSafeStain, Invitrogen). The putative bands containing TIAM1 were excised, de-stained and tryptically digested. Gel sections were de-stained by addition of 1 ml of 200 mM ammonium bicarbonate in 40% (v/v) acetonitrile for 20 min at 37°C (3 changes in total). Gel pieces were then dehydrated by addition of 1 ml acetonitrile for 10 min followed by air drying for 20 min. Proteins were digested by adding 100 µl of 40 mM ammonium bicarbonate, 9% (v/v) acetonitrile containing 100 ng of sequencing grade trypsin (Sigma-Aldrich), gel bands rehydrated and 200 µl of 40 mM ammonium bicarbonate, 9% (v/v) acetonitrile added and incubated with the proteins for 16 h at 37ºC. Resulting peptides were dried and resuspended in water containing 0.1% formic acid. Nano flow liquid chromatography was performed using a nano Acquity Ultra High pressure Liquid Chromatography (LC) system. Sample was loaded onto a trap column (Waters Symmetry C18, 180 µm ID, 20 mm 5 µm particles) at 7 µl/min in 1% acetonitrile, 0.1% formic acid for 5 min. Peptides were separated with a linear gradient of acetonitrile 0.1% formic acid from 1-40% over 40 min at a flow rate of 400 nl/min at 65°C (Waters BEH 130 C18 75 µm, ID 250 mm 1.7 µm particles). The LTQ-Orbitrap XL mass spectrometer was used in a data dependent mode. This consisted of a 60,000 resolution FTMS scan followed by up to 6 (most intense

multiply charged precursors between m/z 400-2000) CID MS/MS (at a normalised collision energy of 35%) events performed in parallel in the linear ion trap. Dynamic exclusion was set to 20 s to reduce oversampling of the same precursor. Data was database searched using mascot 2.0 utilising custom variable K modifications of GG and LRGG in addition to conventional variable modifications of M132 oxidation and S,T,Y phosphorylation. Any potential sites of GG or LRGG modification were manually validated. Similarly *in vitro* reactions were carried out as described above and after SDS-PAGE the gel was stained using Coomasie (SimplyBlueSafeStain, Invitrogen). The putative bands containing ubiquitylated TIAM1 were excised, de-stained and tryptically digested and LC-MS/MS performed as above to identify PHnCCEx TIAM1 domain ubiquitylation sites.

RT-PCR. Total RNA was extracted from MDCKII cells either treated with HGF or expressing the inducible HUWE1 knock-down system using RNeasy® Plus Mini kit (Qiagen) following the supplier's protocol. Cells were lysed in 350 µl of Buffer RLT Plus provided in the kit and homogenised by passing through a 1 ml syringe and 0.5 mm needle 6 times. Subsequent steps were carried out according to the protocol with the following modification: total RNA was eluted in 25 µl of RNase-free water provided. The concentration of total RNA extracted was determined using a nanodrop® spectrometer (Thermo Scientific). cDNA was synthesised from 1 µg of the total RNA using oligo dT_{12-18} primer (Invitrogen) and First-Strand cDNA Synthesis kit (Invitrogen), following the supplier's protocol. The cDNA was then subjected to quantitative real-time PCR (qRT-PCR) using an ABI 7900 system (Applied Biosystems). Relative quantitation was performed using the ΔΔCT method.

Halo pulse chase analysis. Briefly MDCKII cells expressing inducible Halo-tagged TIAM1 were seeded at a density of $2x10^3$ cells on a 35 mm glass bottom dish. The following day, cells were treated with 1 µg/ml of Dox to induce expression of the Halo-tagged TIAM1 protein for 24 h before being serum starved for 18 h. Cells were then labelled with 50 nM of HaloTag® TMR Ligands (Promega, Cat. # G8252) in DMEM for 10 min, washed 3 times and Leibovitz medium (Invitrogen) in the presence or absence of 10 ng/ml HGF was added. During the duration of the HGF (or control) treatment 50 µM of blocking reagent Succinimidyl Ester O4 (Promega) was also added. Cells were visualized by live-imaging on an Olympus IX71 microscope. Z-stacks of 0.2 µm per section for a total height of 10 µm were taken for each cell, every 5 min for a total of 1 h. ImageJ was used to quantify Halo-TIAM1 signal at the cell–cell adhesions by manually selecting the regions and then analysed

using the ‘Intensity’ function. Average intensity values of Halo-TIAM1signals for different time points were normalized to 0 min which was set as 1. At least 30 cells per experiment were quantified from 3 independent experiments.

Duolink PLA®. This technique was performed using the Duolink II Red Starter Kit (Sigma) and its basis can be found in (Soderberg et al., 2006). A video summarizing the steps of this technique can be also found online (www.olink.com/products-services/duolink/how-useduolink). Briefly, cells were seeded at low density on glass coverslips and left to attach for 24 h. Cells were then fixed with 3.7 % formaldehyde in PBS and permeabilised for 10 min in TBS (25 mM Tris-HCl [pH 8.0], 150 mM NaCl, 0.1 % Tween-20) containing 0.5 % Triton X-100. Samples were incubated with 3% BSA for 1 h at 37ºC in a humidity chamber and then overnight at 4ºC with an anti-HA mouse and rabbit polyclonal antibody against K48 linked Ub or alternatively a polyclonal rabbit TIAM1 antibody and mouse monoclonal HUWE1 antibody. Cells were also stained with a rat monoclonal E-CADHERIN antibody to mark cell–cell adhesions. Slides were then incubated for 1 h at 37ºC with a mix of the MINUS (anti-mouse) and PLUS (anti-rabbit) PLA probes. Hybridized probes were ligated using the Ligation-Ligase solution for 30 min at 37ºC and then amplified utilizing the Amplification-Polymerase solution for 100 min at 37ºC. Slides were finally mounted using Duolink II Mounting Medium with DAPI and imaged using the Zeiss Axiovert 200M enclosed in a full environmental chamber (Solent Scientific).

Transwell migration and invasion experiments. MDCKII cells or H1299, H358, H522 or H596 lung cancer cells transfected with non-targeting TIAM1, HUWE1 or a combination of TIAM1/HUWE1 siRNAs or overexpressing WT or 4x595R TIAM1 were seeded onto 8 mm transwell migration chambers (Corning, #3422) whose membranes had been pre-coated with either 500 µg/ml (long term invasion assays) or 50 µg/ml (24 h invasion assays) Collagen type I derived from Rat tail (BD Biosciences, #354429). Cells were incubated with serum-free DMEM (MDCKII cell line) or RPMI (lung cancer cell lines) or DMEM (MDCKII) or RPMI (lung cancer cell lines) with 20ng/ml HGF in 10% FBS, added to the lower chamber. Media, including HGF, was replaced every 2 days for the long term 120 h invasion assay. In addition transfection of TIAM1/HUWE1 in MDCKII and H1299 cells was repeated 72 h after the original transfection. MDCKII cells were left to invade for 5 days and H1299 4 days. Cells on the upper part of the membrane were scraped off using a cotton swab and the migrated cells

were fixed in 100% methanol and stained with 0.4% Crystal Violet in 10% ethanol. The experiment was performed in triplicates for all conditions described. From every transwell at least 10 images were taken under a phase-contrast microscope at x 20 magnification. Crystal Violet was then eluted with 33% (v/v) acetic acid in distilled H_2O and 100 µl transferred to a 96-well plate. Absorbance was read at 600 nM and relative invasion determined by plotting optical density against a standard curve of MDCKII, H1299, H358 or H522 or H596 cells previously prepared and normalizing for cell numbers.

Zebrafish xenograft experiments. Zebrafish xenograft experiments were performed as has been previously described (Chapman et al., 2014). Briefly, adult Zebrafish (*Danio rerio*) were maintained at The University of Manchester Biological Services Unit according to National Home Office regulations under the Animals (Scientific Procedures) Act 1986. *Casper* strain (*roy*$^{-/-}$, *nacre*$^{-/-}$) Zebrafish were used as a transparent model to facilitate imaging. H1299 cells were stained using 20 µM Cell Trace^{TM} CFSE Cell Proliferation Kit (Life Technologies) for 15 min at 37°C, followed by a 30 min wash in RPMI medium. Cells were resuspended on ice at 1.3x10^{7}/ml in PBS with 0.5% polyvinylpyrrolidone K60 solution (PVP, Sigma). 48 hours post fertilisation (hpf) embryos were anaesthetised with 0.1mg/ml MS222 (Sigma) and approximately 500 cells were injected into the pericardial cavity using a micropipette and pump (World Precision Instruments). Engrafted embryos were sorted to remove uninjected embryos and maintained at 34°C for a further 3 days. 4 days post-injection (dpi) engrafted Zebrafish were mounted in 1.5% low melting agarose (LMP, Flowgen Biosciences). Tumours were imaged using a Leica TCS SP5 AOBS upright confocal (Leica Microsystems) using a 20 x 0.50 Plan Fluotar dipping objective and 1.3 x confocal zoom. Captured z stacks were processed using Volocity software (Perkin Elmer, Cambridge, UK). All experiments consist of a minimum of three independent repeats. Relative Invasion Index (RII) is defined as the average number of cells invaded outside the pericardial cavity at 4dpi normalized to the average number in a control group.

Immunohistochemical analysis. Scoring was assessed using semi quantitative scoring (H-scores) of the nucleus, membrane and cytoplasm. H-scores were derived from a semi quantitative assessment of both staining intensity (scale 0–3) and the percentage of positive cells (0–100%), which, when multiplied, generated a score ranging from 0 to 900 for all three proteins. TMA immunohistochemistry was evaluated independently by two investigators (D.N. and L.V) and where

discordance (i.e. different scores given by different investigators) was found cases were re-evaluated and a consensus reached. Ethical approval for these studies was conferred under the MCRC Biobank Research Tissue Bank Ethics (ref: 07/H1003/161+5) from NRES Committee North West - Greater Manchester South Ethical.

SUPPLEMENTAL REFERENCES

Chapman, A., Fernandez del Ama, L., Ferguson, J., Kamarashev, J., Wellbrock, C., and Hurlstone, A. (2014). Heterogeneous tumor subpopulations cooperate to drive invasion. Cell Rep *8*, 688-695.

Chen, D., Kon, N., Li, M., Zhang, W., Qin, J., and Gu, W. (2005). ARF-BP1/Mule is a critical mediator of the ARF tumor suppressor. Cell *121*, 1071-1083.

Choo, Y.S., and Zhang, Z. (2009). Detection of protein ubiquitination. J Vis Exp.

Holden, P., and Horton, W.A. (2009). Crude subcellular fractionation of cultured mammalian cell lines. BMC Res Notes *2*, 243.

Mack, N.A., Porter, A.P., Whalley, H.J., Schwarz, J.P., Jones, R.C., Khaja, A.S., Bjartell, A., Anderson, K.I., and Malliri, A. (2012). beta2-syntrophin and Par-3 promote an apicobasal Rac activity gradient at cell-cell junctions by differentially regulating Tiam1 activity. Nat Cell Biol *14*, 1169-1180.

Michiels, F., Stam, J.C., Hordijk, P.L., van der Kammen, R.A., Ruuls-Van Stalle, L., Feltkamp, C.A., and Collard, J.G. (1997). Regulated membrane localization of Tiam1, mediated by the NH2-terminal pleckstrin homology domain, is required for Rac-dependent membrane ruffling and C-Jun NH2-terminal kinase activation. J Cell Biol *137*, 387-398.

Soderberg, O., Gullberg, M., Jarvius, M., Ridderstrale, K., Leuchowius, K.J., Jarvius, J., Wester, K., Hydbring, P., Bahram, F., Larsson, L.G., *et al.* (2006). Direct observation of individual endogenous protein complexes in situ by proximity ligation. Nat Methods *3*, 995-1000.

Terawaki, S., Kitano, K., Mori, T., Zhai, Y., Higuchi, Y., Itoh, N., Watanabe, T., Kaibuchi, K., and Hakoshima, T. (2010). The PHCCEx domain of Tiam1/2 is a novel protein- and membrane-binding module. EMBO J *29*, 236-250.

Woodcock, S.A., Rooney, C., Liontos, M., Connolly, Y., Zoumpourlis, V., Whetton, A.D., Gorgoulis, V.G., and Malliri, A. (2009). SRC-induced disassembly of adherens junctions requires localized phosphorylation and degradation of the rac activator tiam1. Mol Cell *33*, 639-653.

Zhong, Q., Gao, W., Du, F., and Wang, X. (2005). Mule/ARF-BP1, a BH3-only E3 ubiquitin ligase, catalyzes the polyubiquitination of Mcl-1 and regulates apoptosis. Cell *121*, 1085-1095.
