# Supplementary material for: HUWE1 Ubiquitylates and Degrades the RAC Activator TIAM1 Promoting Cell-Cell Adhesion Disassembly, Migration, and Invasion
Source: Cell Rep. 2014 Dec 24;10(1):88–102. doi: 10.1016/j.celrep.2014.12.012 (PMC4542307; doi:10.1016/j.celrep.2014.12.012)
Supplement: Document S2. Article plus Supplemental Information [file mmc2.pdf]

Cell Reports

Article

HUWE1 Ubiquitylates and Degrades the RAC Activator TIAM1 Promoting Cell-Cell Adhesion Disassembly, Migration, and Invasion

Graphical Abstract

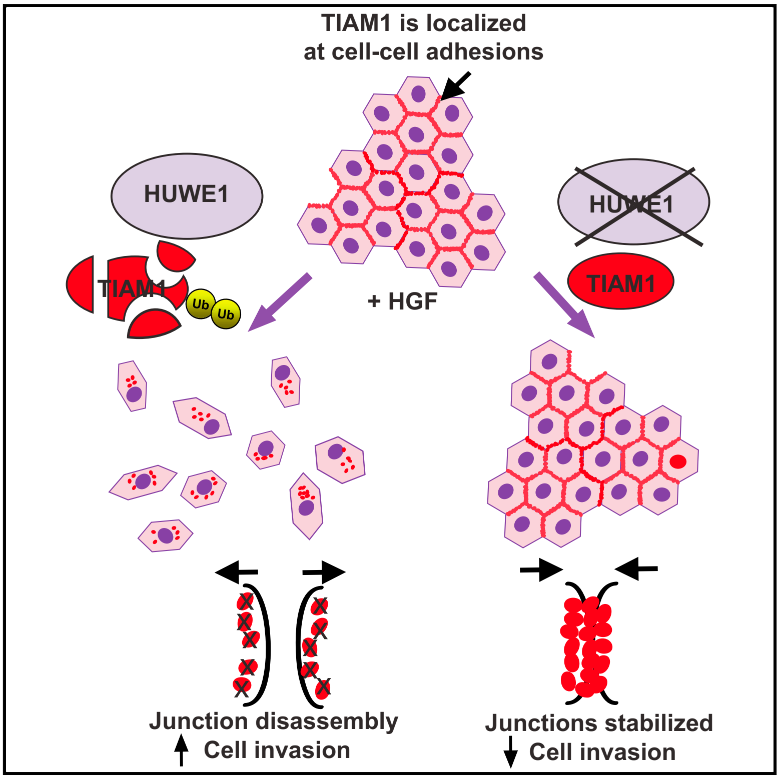

Highlights

- HGF-induced cell-cell adhesion disassembly, migration, and invasion require HUWE1
- HUWE1 ubiquitylates and degrades TIAM1 at cell-cell adhesions in response to HGF
- TIAM1 is the key HUWE1 target regulating cell-cell adhesion, motility, and invasion
- HUWE1 and TIAM1 expression levels are inversely correlated in lung cancer

Authors

Lynsey Vaughan, Chong-Teik Tan, ..., Adam F.L. Hurlstone, Angeliki Malliri

Correspondence

angeliki.malliri@cruk.manchester.ac.uk

In Brief

Malignant conversion of epithelial tumor cells is incompletely understood. Here, Vaughan et al. demonstrate that the E3 ligase HUWE1 degrades the RAC activator TIAM1 following HGF stimulation, promoting cell junction disassembly, motility, and invasion in epithelial cells including lung cancer cells. This indicates a critical role for HUWE1 in lung cancer dissemination.

CrossMark

Vaughan et al., 2015, Cell Reports *10*, 88–102
January 6, 2015 ©2015 The Authors
http://dx.doi.org/10.1016/j.celrep.2014.12.012

CellPress

HUWE1 Ubiquitylates and Degrades the RAC Activator TIAM1 Promoting Cell-Cell Adhesion Disassembly, Migration, and Invasion

Lynsey Vaughan,[1] Chong-Teik Tan,[1] Anna Chapman,[2] Daisuke Nonaka,[3] Natalie A. Mack,[1] Duncan Smith,[4] Richard Booton,[5] Adam F.L. Hurlstone,[2] and Angeliki Malliri[1,*]
[1]Cell Signalling Group, Cancer Research UK Manchester Institute, The University of Manchester, Manchester M20 4BX, UK
[2]Faculty of Life Sciences, The University of Manchester, Oxford Road, Manchester M13 9PT, UK
[3]Department of Histopathology, The Christie Hospital and Institute of Cancer Sciences, The University of Manchester, Manchester M20 4BX, UK
[4]Biological Mass Spectrometry, Cancer Research UK Manchester Institute, The University of Manchester, Manchester M20 4BX, UK
[5]Respiratory Research Group, Institute of Inflammation and Repair, The University of Manchester and North West Lung Centre, University Hospital of South Manchester, Manchester M23 9LT, UK
*Correspondence: angeliki.malliri@cruk.manchester.ac.uk
http://dx.doi.org/10.1016/j.celrep.2014.12.012
This is an open access article under the CC BY license (http://creativecommons.org/licenses/by/3.0/).

SUMMARY

The E3 ubiquitin ligase HUWE1, deregulated in carcinoma, has been implicated in tumor formation. Here, we uncover a role for HUWE1 in cell migration and invasion through degrading the RAC activator TIAM1, implying an additional function in malignant progression. In MDCKII cells in response to HGF, HUWE1 catalyzes TIAM1 ubiquitylation and degradation predominantly at cell-cell adhesions, facilitating junction disassembly, migration, and invasion. Depleting HUWE1 or mutating the TIAM1 ubiquitylation site prevents TIAM1 degradation, antagonizing scattering, and invasion. Moreover, simultaneous depletion of TIAM1 restores migration and invasion in HUWE1-depleted cells. Significantly, we show that HUWE1 stimulates human lung cancer cell invasion through regulating TIAM1 stability. Finally, we demonstrate that HUWE1 and TIAM1 protein levels are inversely correlated in human lung carcinomas. Thus, we elucidate a critical role for HUWE1 in regulating epithelial cell-cell adhesion and provide additional evidence that ubiquitylation contributes to spatiotemporal control of RAC.

INTRODUCTION

Metastasis, a multistep process beginning with local invasion and culminating in the colonization of distant organs by cancer cells, is responsible for more than 90% of all cancer deaths (Sleeman and Steeg, 2010). Metastasis of carcinoma cells often commences with the disassembly of junctional complexes and downregulation of other epithelial traits coupled with the acquisition of a migratory and invasive mesenchymal phenotype (so-called epithelial-mesenchymal transition [EMT]). EMT is elicited by growth factors such as hepatocyte growth factor (HGF) secreted by tumor and stromal cells. Acting through its cognate receptor, c-MET, HGF induces rapid disassembly of adherens junctions through stimulating the ubiquitylation and associated proteasomal degradation of junctional proteins like E-CADHERIN (Fujita et al., 2002).

Ubiquitylation—the covalent attachment of ubiquitin to lysine residues on a target protein—is carried out by three enzymes: ubiquitin activating enzyme (E1), ubiquitin-conjugase (E2), and ubiquitin ligase (E3), each comprising a family of proteins. The HECT, UBA, and WWE domain-containing protein 1 (HUWE1) is a member of the HECT E3 ubiquitin ligase family whose substrates include key proteins such as p53 and MYC (Adhikary et al., 2005; Chen et al., 2005), which regulate diverse cellular responses including proliferation and survival with often opposing outcomes. Unsurprisingly, HUWE1 has been ascribed both putative oncoprotein and tumor suppressor functions. Adding to this controversy, HUWE1 is overexpressed in some cancers but downregulated in others (Adhikary et al., 2005; Zhao et al., 2009). Clearly, further investigation is required to resolve the contribution of HUWE1 to tumorigenesis.

The T lymphoma invasion and metastasis inducing protein 1 (TIAM1) is a guanine nucleotide exchange factor (GEF) that activates the small GTPase RAC (Michiels et al., 1995). It shows perturbed expression in various cancers including colon, breast, and lung (Minard et al., 2005; Stebel et al., 2009; Wang and Wang, 2012). Previously, we showed that *Tiam1* knockout mice are resistant to H-Ras-induced skin tumors (Malliri et al., 2002), implying a requirement for TIAM1 in tumor formation consistent with its roles in cell proliferation and survival (Rygiel et al., 2008). Intriguingly, the few tumors developing in *Tiam1*$^{-/-}$ mice were more frequently malignant (Malliri et al., 2002), suggesting that TIAM1 antagonizes malignant progression. Supporting this, TIAM1-RAC activation restored an epithelial-like phenotype and suppressed invasiveness in RAS-transformed MDCKII cells (Hordijk et al., 1997). Additionally, TIAM1 depletion in nontransformed MDCKII cells lead to the disassembly of

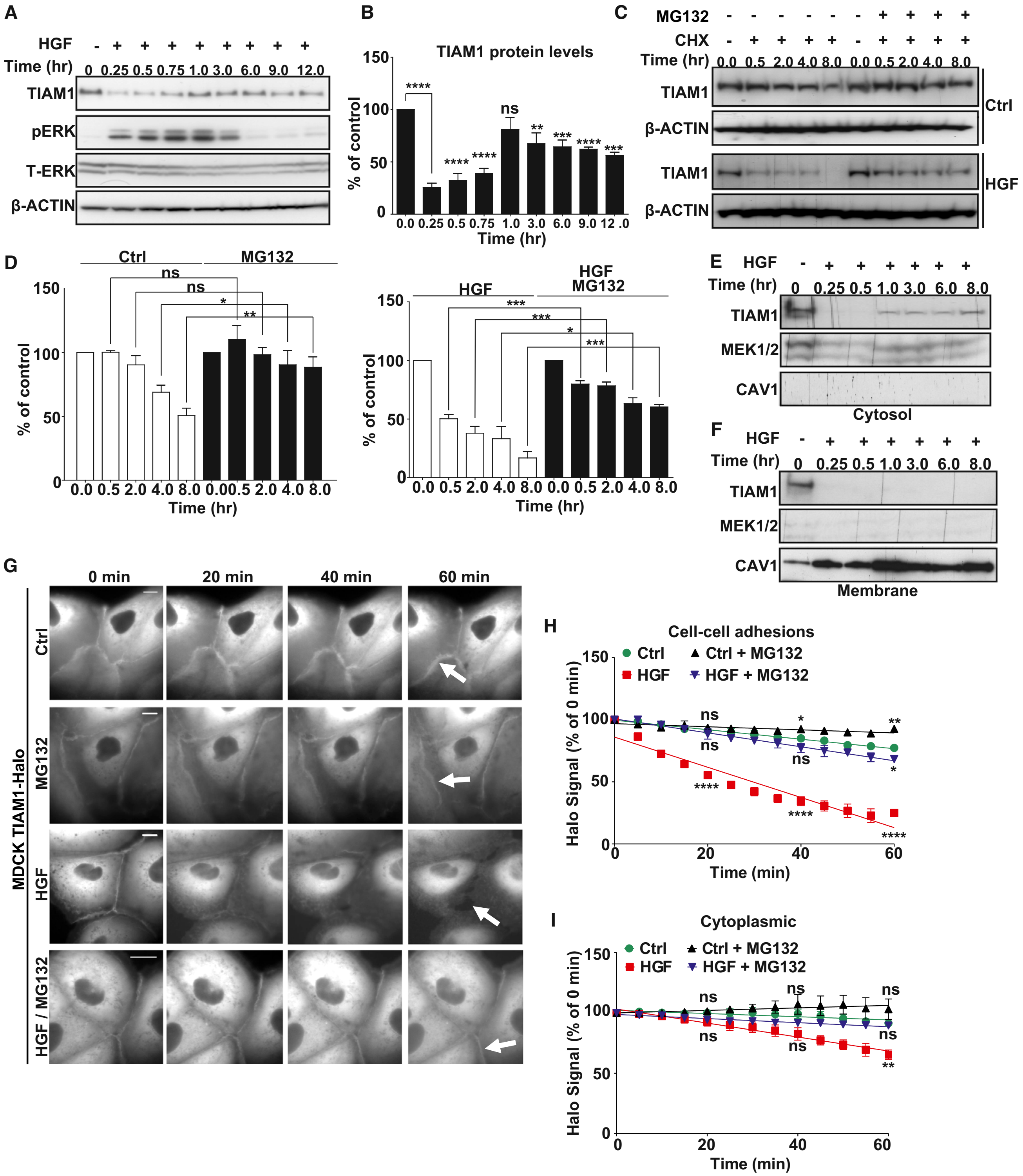

Figure 1. HGF Stimulation Induces TIAM1 Degradation

(A) MDCKII cells were treated with 10 ng/ml HGF for the indicated times, and immunoblotting was performed. pERK was used as readout of HGF activity and β-ACTIN and total ERK (T-ERK) as loading controls.

(B) Quantification of TIAM1 in (A) from three independent experiments. Mean values ±SE. ****$p < 0.0001$, ***$p < 0.0005$, **$p < 0.01$; ns, not significant (unpaired t test).

(legend continued on next page)

OPEN
ACCESS
CellPress

their cadherin-based adhesions, acquisition of a flattened morphology and increased motility (Malliri et al., 2004). Collectively, these findings indicate that TIAM1 promotes cadherin-based adhesion. Consistent with a role as an invasion suppressor, TIAM1 protein expression is decreased during breast cancer progression (Stebel et al., 2009). However, the TIAM1-RAC signaling module can also enhance cell migration and invasion through promoting lamellipodia and invadopodia (Bourguignon et al., 2000). Promigratory/proinvasive roles of TIAM1-RAC manifest when cells are unable to form intercellular adhesions, e.g., when plated sparsely or on collagen substrates, or in cells intrinsically lacking E-cadherin, e.g., lymphoma cells (Habets et al., 1995; Sander et al., 1998). Reflecting its functional diversity, TIAM1 protein has been detected at intercellular junctions, the Golgi apparatus, the cytosol, and membrane protrusions (Adams et al., 2010; Mack et al., 2012; Michiels et al., 1995; Woodcock et al., 2009). We postulate that changes in TIAM1 local concentration brought about by the ubiquitin-proteasome pathway could impact upon the resultant outcome of TIAM1 stimulation. Potentially, selective degradation of TIAM1 at cell-cell adhesions triggering their disassembly could preserve the growth, survival, and dissemination stimulatory properties of TIAM1-RAC in malignantly transformed cells, while diminishing their dissemination suppressing properties.

Here, we show that in response to HGF, HUWE1 ubiquitylates TIAM1 on lysine 595, triggering its proteasomal degradation predominantly at cell-cell adhesions, thereby enabling disassembly of cell junctions and induction of cell migration and invasion, including in lung carcinoma cells. We also show that TIAM1 and HUWE1 protein levels are negatively correlated in early-stage lung cancer specimens, consistent with this regulatory mechanism operating in human tumors.

RESULTS

HGF Stimulates Proteasomal Degradation of TIAM1 at Cell-Cell Junctions

We reasoned that TIAM1 may be downregulated in response to stimuli that disrupt cell-cell adhesion and induce motility. To test this hypothesis, we utilized MDCKII cells that in response to HGF disassemble their cell-cell adhesions and scatter (Uehara and Kitamura, 1992). We detected a transient and profound decrease of TIAM1 protein during the first hour of HGF treatment, followed by a secondary less-marked reduction persisting to 12 hr after HGF treatment (Figures 1A and 1B). Moreover, we observed scattering of colonies of MDCKII cells within this time frame (Figures S1A and S1B). We then measured the effect of HGF stimulation on the turnover of TIAM1 protein by inhibiting new protein synthesis with cycloheximide. Turnover was greatly increased in cells stimulated with HGF compared to control cells (Figures 1C and 1D). Furthermore, downregulation was via protein degradation as we observed no significant changes in the amount of mRNA following HGF treatment (Figure S1C), and the decrease in TIAM1 protein levels was significantly rescued by treating cells with MG132, a reversible proteasome inhibitor (Figures 1C and 1D).

To investigate if specific subcellular fractions of TIAM1 were subject to preferential degradation, we treated MDCKII cells with HGF and isolated both cytosolic and membrane protein fractions over a time course of 8 hr. A significant decrease of endogenous TIAM1 in both membrane and cytosolic fractions was observed 0.25 hr after HGF treatment (Figures 1E and 1F). However, as seen for the total cell lysate (Figures 1A and 1B), the decrease in cytosolic TIAM1 was partially reversed, whereas the depletion of the membrane fraction was sustained through all subsequent time points (Figures 1E and 1F). To further examine the decrease in TIAM1 seen within the first hour of HGF stimulation, we engineered MDCKII cells to inducibly express Halo-tagged TIAM1 and analyzed the downregulation of TIAM1-Halo at cell-cell adhesions as well as in the cytoplasm using a pulse-chase method described elsewhere (Yamaguchi et al., 2009). We detected significant TIAM1 depletion from cell-cell adhesions but less so from the cytoplasm of HGF-stimulated cells (Figures 1G–1I). Furthermore, TIAM1 depletion could be rescued by inhibiting the proteasome (Figures 1G–1I). We therefore conclude that HGF stimulation induces the preferential depletion of TIAM1 from cell-cell adhesions.

TIAM1 Is Ubiquitylated in HGF-Stimulated Cells

We next tested whether TIAM1 was modified by ubiquitin (Ub) during the initiation of HGF-induced cell scattering. Endogenous TIAM1 was immunoprecipitated under denaturing conditions and probed for ubiquitin. A significant accumulation of ubiquitylated TIAM1 was observed following HGF treatment compared to untreated control (Figure 2A). K48-Ub chains are considered the primary signal for proteasomal degradation, and attachment of four or more Ub molecules to the protein is sufficient to target proteins to the proteasome (Chau et al., 1989). Using a specific K48-linked ubiquitin antibody to probe TIAM1 immunoprecipitated under denaturing conditions, we detected a significant increase of K48-linked TIAM1 after HGF treatment (Figure 2B). Moreover, this ubiquitin smear was specific to TIAM1, because downregulating TIAM1 using two different small interfering RNAs (siRNAs) substantially decreased it (Figure 2C). As further confirmation, having engineered MDCKII cells to express

(C) MDCKII cells were pretreated with 5 μM MG132 for 3 hr where indicated, and 10 ng/ml HGF and 50 μg/ml cycloheximide (CHX) were added at 0 hr. Protein extracted at the indicated times was analyzed by immunoblotting.
(D) Quantification of TIAM1 in (C). Mean values ±SE ***$p < 0.0005$, **$p < 0.01$, and *$p < 0.05$; ns, not significant (unpaired t test).
(E and F) MDCKII cells were treated with 10 ng/ml HGF for the indicated times. Cytosolic (E) and membrane (F) fractions were prepared and TIAM1 monitored by immunoblotting. MEK1/2 and CAV1 were used as cytosolic and membrane specific markers, respectively.
(G) MDCKII cells expressing Halo-tagged TIAM1 were labeled with 50 nM of fluorescent TMR ligand, and Halo-tagged TIAM1 was chased through addition of the HaloTag blocking agent. Live imaging was performed in the presence or absence of HGF for 1 hr. White arrows indicate Halo-tagged TIAM1 at intercellular junctions. Scale bar, 10 μm.
(H and I) Fluorescence intensity of Halo-tagged TIAM1 in MDCKII cells was measured for 30 cell-cell adhesions (H) and cytoplasmic pools (I) from three biological replicates. Mean values ±SE ****$p < 0.0001$, **$p < 0.01$, *$p < 0.05$; ns, not significant (unpaired t test).
See also Figure S1.

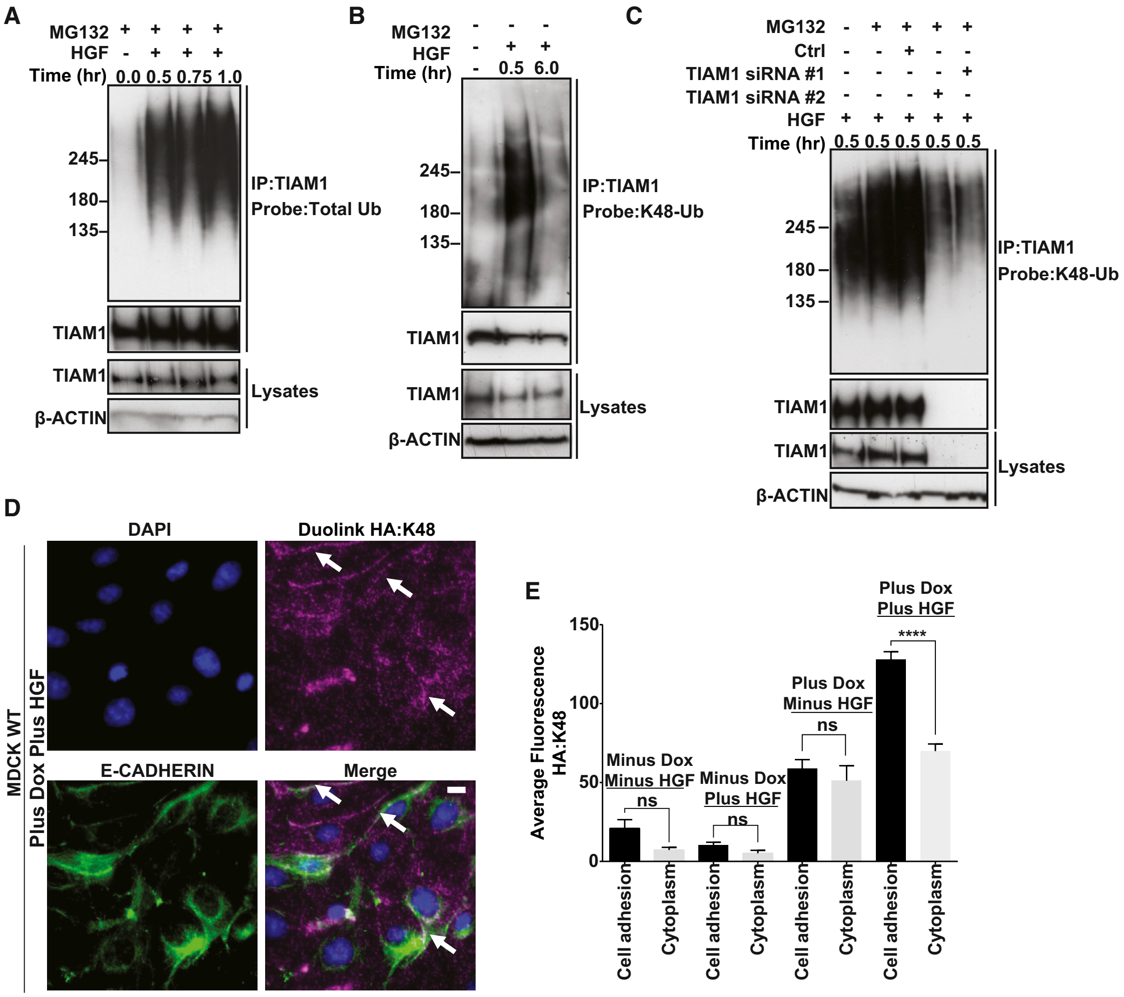

Figure 2. TIAM1 Is Ubiquitylated following HGF Stimulation

(A) MDCKII cells were pretreated (3 hr) with 5 μM MG132 and treated with 10 ng/ml HGF for the times indicated. Cells were then lysed and endogenous TIAM1 immunoprecipitated (IP) under denaturing conditions and probed for total ubiquitin.

(B) As for (A) but without pretreatment with MG132 and immunoprecipitates were probed using a K48-linkage specific antibody.

(C) MDCKII cells were transfected with a nontargeting siRNA or two siRNAs targeting TIAM1 (#1 and #2). At 96 hr following transfection, cells were treated with 10 ng/ml HGF and protein was extracted at 0.5 hr. TIAM1 was immunoprecipitated under denaturing conditions and probed with a K48-linkage specific antibody.

(D) MDCKII cells were pretreated (3 hr) with 5 μM MG132 and with 10 ng/ml HGF for 1 hr and fixed, and the Duolink protocol was performed to monitor the extent and localization of K48 ubiquitylated TIAM1-HA induced following addition of doxycycline (Plus Dox). White arrows indicate intercellular junctions. Scale bar, 10 μm.

(E) Quantification of Duolink signal in (D) and also in uninduced (Minus Dox) cells. Mean values ±SE of three independent experiments. ****$p < 0.0001$; ns, not significant (unpaired t test).

See also Figure S2.

HA-tagged TIAM1 following addition of doxycycline, the Duolink proximity ligation assay (Duolink PLA) revealed a large increase in TIAM1-HA ubiquitylated with K48-linked Ub in HGF-stimulated cells compared to untreated cells (Figures 2D, 2E, and S2). Although TIAM1 ubiquitylated with K48-linked Ub was distributed throughout the cell, it was enriched at cell-cell adhesions (Figures 2D, 2E, and S2, see white arrows). These data suggest that TIAM1 is ubiquitylated and degraded via K48-linked Ub during the early stages of epithelial cell scattering, and that this degradation occurs more at cell-cell adhesions than in the cytoplasm.

The E3 Ligase HUWE1 Ubiquitylates TIAM1 in HGF-Stimulated Cells

We previously performed tandem affinity purification (TAP) of protein complexes from *Tiam1*$^{-/-}$ mouse embryonic fibroblasts expressing TAP-tagged TIAM1 (Mack et al., 2012). This approach also identified the HUWE1 E3 ubiquitin ligase as a binding partner of TIAM1 (data not shown). This interaction was validated in vivo using coimmunoprecipitation (co-IP) both with endogenous proteins in MDCKII cells and exogenous TIAM1 in HEK293T cells (Figure 3A; Figure S3A, respectively). Notably, the interaction of TIAM1 and HUWE1 was greatly

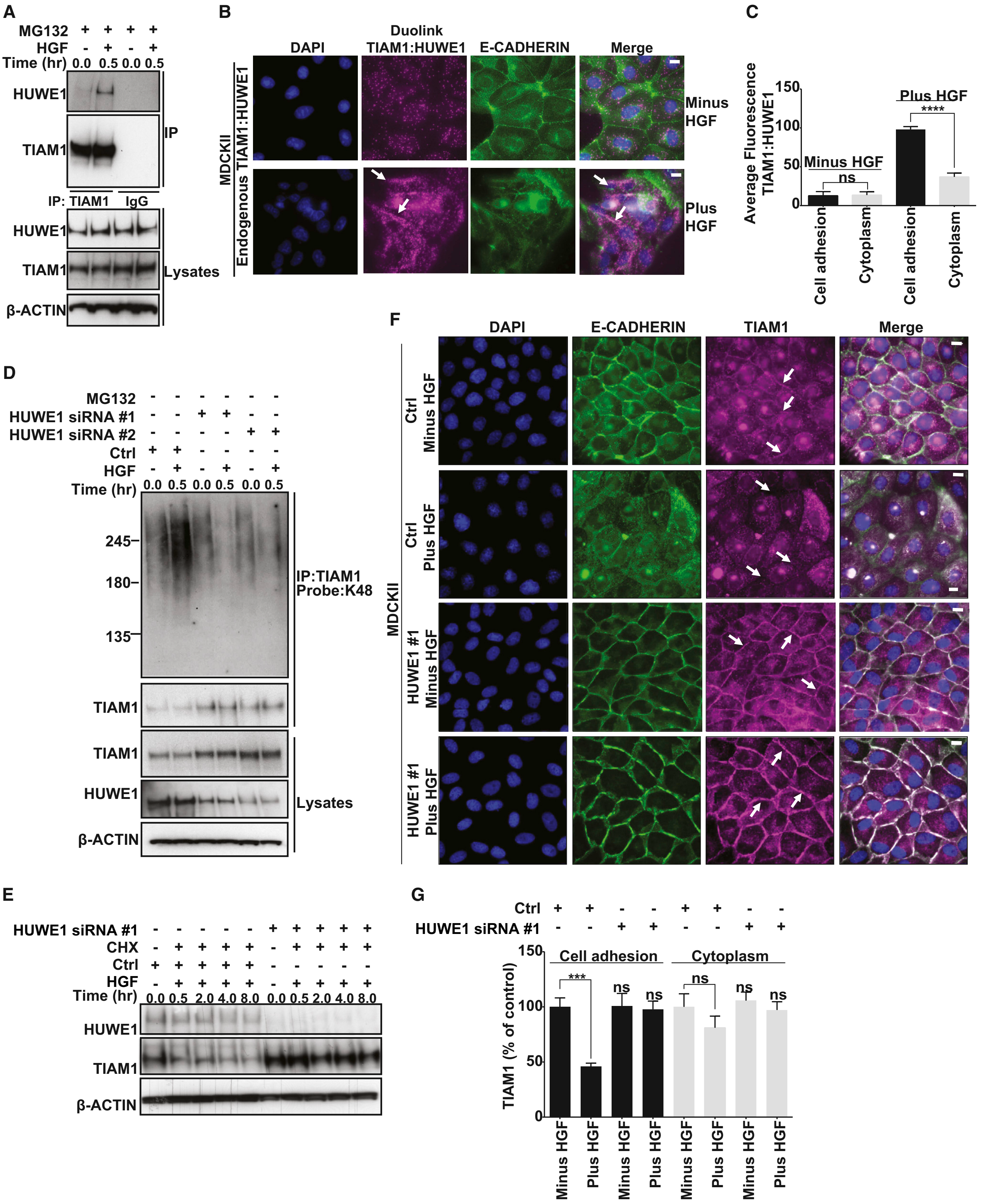

(legend on next page)

increased in the presence of HGF (Figure 3A). Immunofluorescence revealed that, in addition to its previously described nuclear localization, HUWE1 in MDCKII cells could be clearly observed at cell-cell junctions, colocalizing with both E-CADHERIN and TIAM1 (Figure S3B). The Duolink PLA assay also showed a marked increase in the interaction of endogenous TIAM1 and endogenous HUWE1 following HGF stimulation, which again while visualized throughout the cell was significantly more pronounced along cell-cell adhesions (Figures 3B and 3C).

The documented role of HUWE1 as an E3 ligase indicates that it could be responsible for regulating the stability of TIAM1 through its ubiquitylation activity. We therefore depleted HUWE1 using different shRNA and siRNA sequences. This resulted in stabilization of TIAM1 protein (Figures S3C and S3D) but not mRNA (Figure S3E) and a concomitant reduction of TIAM1 K48-linked ubiquitylation (Figures 3D and S3F–S3H). Moreover, the increased turnover of TIAM1 in cells stimulated with HGF was suppressed by HUWE1 depletion (Figures 3E, S3I, and S3J). Further, immunofluorescence was performed in either control or HUWE1-depleted cells before and after HGF treatment (Figure 3F). In cells treated with a nontargeting control oligo, TIAM1 was significantly depleted from cell-cell adhesions but not the cytoplasm following HGF stimulation (Figures 3F and 3G). Significantly, depletion of HUWE1 was able to stabilize TIAM1 levels at cell-cell adhesions in HGF-treated cells (Figures 3F and 3G). These observations strongly imply that HUWE1 is largely responsible for the polyubiquitylation and degradation of TIAM1 at cell-cell adhesions during the induction of epithelial cell scattering.

HUWE1-Mediated TIAM1 Degradation Controls HGF-Induced Motility and Invasion of Epithelial Cells

To investigate the potential role of HUWE1 in cell-cell adhesion and migration and whether this might be through regulating TIAM1, we depleted HUWE1 and TIAM1 alone and in combination and assessed the effect on HGF-induced cell motility. Cell scattering was monitored by phase contrast microscopy (Figures 4A and S4A) and cells were lysed at 18 hr to confirm TIAM1 and HUWE1 knockdown by immunoblotting (Figure S4B). MDCKII cells with depleted HUWE1 scattered significantly less than cells transfected with the nontargeting control, whereas, in contrast, scattering in cells with depleted TIAM1 was moderately enhanced following HGF treatment (Figures 4A, 4B, and S4A). To examine if the decreased scattering of HUWE1-depleted cells was due to an increase in TIAM1 at cell-cell adhesions (as seen in Figures 3F and 3G), we knocked down both HUWE1 and TIAM1 using two different combinations of siRNA in MDCKII cells. Interestingly, simultaneous depletion of both TIAM1 and HUWE1 significantly rescued the scattering of MDCKII cells (Figures 4A, 4B, and S4A).

Next, we investigated the role of HUWE1-mediated TIAM1 degradation in cell invasion. Seventy-two hours posttransfection, MDCKII cells in which HUWE1, TIAM1, or a combination of both were depleted were assayed for short-term (24 hr) and long-term (120 hr) invasive potential using modified Boyden chamber assays coated with collagen. Transfected cells were also seeded in 24-well plates to measure total cell number (in order to normalize cellular invasion to cell number) and 6-well plates to measure knockdown of TIAM1 and HUWE1 protein expression (Figure S4C, top) at the end of the experiment. Invasion was quantified by crystal violet staining of cells, which had invaded through both collagen and insert membrane and normalized to total cell number. Both short-term (Figures 4C and 4D) and long-term (Figures S4D and S4E) invasion assays resulted in similar patterns of invasion. Depletion of HUWE1 alone greatly inhibited the invasion of MDCKII cells stimulated by HGF. In contrast, the invasion of TIAM1-depleted cells showed no significant change (Figures 4C, 4D, S4D, and S4E). Interestingly, the ability of MDCKII cells to invade was significantly restored in cells where both HUWE1 and TIAM1 were downregulated simultaneously (Figures 4C, 4D, S4D, and S4E).

The above findings in MDCKII cells raised the possibility that aberrant HUWE1 regulation of TIAM1 might occur during tumorigenesis, potentially contributing to malignant progression. HUWE1 is significantly overexpressed in a number of epithelial tumors including lung carcinoma (Confalonieri et al., 2009). However, no invasive role had thus far been attributed to HUWE1. To understand if the above mechanism is relevant in lung carcinoma, we obtained a number of lung carcinoma cell lines, including the H1299, H358, H522, and H596 cell lines, which contain nonfunctional p53 (as depletion of HUWE1 in some settings has been shown to result in stabilization of wild-type p53

Figure 3. TIAM1 Is Ubiquitylated by HUWE1

(A) MDCKII cells were pretreated (3 hr) with 5 μM MG132 and treated with 10 ng/ml HGF as shown, and protein was extracted at the times indicated. Endogenous TIAM1 was immunoprecipitated, and lysates were probed for HUWE1. Lysates were also subjected to IP with an immunoglobulin G control.

(B) MDCKII cells were pretreated with 5 μM MG132 for 3 hr with (Plus HGF) or without (Minus HGF) the addition of 10 ng/ml HGF for 1 hr and fixed, and the Duolink protocol was performed to monitor the extent and localization of the HUWE1 interaction with TIAM1 (both endogenous). White arrows indicate intercellular junctions. Scale bar, 10 μm.

(C) Quantification of the Duolink signal in (B). Mean values ±SE of three independent experiments. ****$p < 0.0001$; ns, not significant (unpaired t test).

(D) MDCKII cells were transfected with HUWE1 siRNA #1 or #2 or a nontargeting siRNA (Ctrl). At 96 hr posttransfection, cells were treated with 10 ng/ml HGF for 0.5 hr where indicated. TIAM1 was immunoprecipitated under denaturing conditions and probed with a K48-linkage specific antibody.

(E) MDCKII cells were transfected with HUWE1 siRNA #1 or a nontargeting siRNA (Ctrl). Cycloheximide (CHX) (50 μg/ml) was added at 96 hr posttransfection, and cells were treated with HGF for the indicated times. Lysates were immunoblotted for TIAM1 and HUWE1. In (A), (D), and (E), β-ACTIN was used as a loading control.

(F) MDCKII cells were transfected with HUWE1 siRNA #1 or a nontargeting siRNA (Ctrl). At 72 hr posttransfection, cells were stimulated with HGF for 1 hr as indicated and fixed. Cells were stained by immunofluorescence (IF) for E-CADHERIN, TIAM1, and DAPI. Panels show representative images from one of three independent experiments. Scale bar, 10 μm.

(G) Quantification of TIAM1 IF levels in (F). Mean values ±SE of three independent experiments. ***$p < 0.0005$; ns, not significant (unpaired t test).

See also Figure S3.

OPEN
ACCESS
CellPress

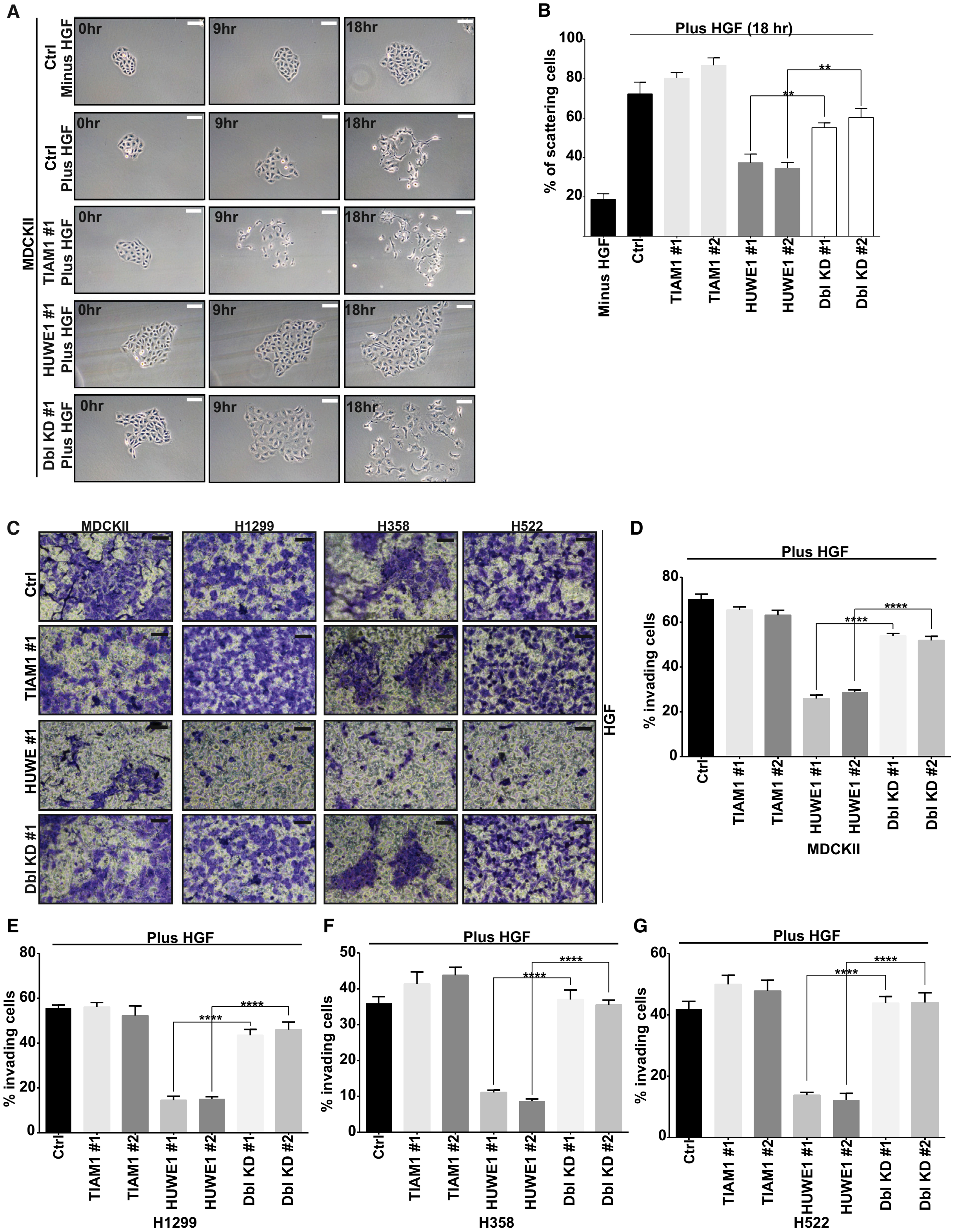

Figure 4. HUWE1-Mediated TIAM1 Degradation Controls HGF-Induced Motility and Invasion

(A) MDCKII cells were transfected with a nontargeting siRNA (Ctrl) or with siRNAs targeting TIAM1 or HUWE1 alone or in combination. Ninety-six hours later, cells were stimulated with 20 ng/ml HGF for 18 hr. Resultant cell scattering was monitored by phase contrast microscopy. Scale bar, 50 μm.

(legend continued on next page)

and consequent cell death [Kon et al., 2012]). Similar to MDCKII cells, depletion of HUWE1 in H1299, H358, H522, and H596 cell lines resulted in increased TIAM1 protein levels (Figure S4C; data not shown). Further and again comparable to the phenotype we observed in MDCKII cells, depletion of HUWE1 in H1299, H358, H522, and H596 cell lines significantly inhibited HGF-induced invasion. In contrast, TIAM1 depletion did not significantly affect invasiveness (Figures 4C, 4E–4G, S4D, and S4F; data not shown). Again, the ability of H1299, H358, H522, and H596 cells to invade was significantly restored in cells where both proteins were downregulated simultaneously (Figures 4C, 4E–4G, S4D, and S4F; data not shown). These results reveal a role for HUWE1 in the invasion of lung carcinoma cells through mediating degradation of TIAM1.

TIAM1 Is Ubiquitylated on Lysine Residue 595

Using mass spectrometry, exogenous TIAM1 immunoprecipitated from HEK293T cells was found to be ubiquitylated by HUWE1 at lysine 595 (K595) (Figure S5A) located in the MGEMQLSSVTDSKKKKTI peptide in the coiled coil region (Figure 5A). In vitro ubiquitylation assays revealed a ladder consistent with the attachment of ubiquitin to TIAM1, which was absent when Ub or ATP was not present (Figure S5B, left) and again mass spectrometry identified lysine 595 of TIAM1 as a HUWE1 ubiquitylation site (data not shown). p53, a previously identified substrate of HUWE1, was used as a positive control for the reaction (Figure S5B, right).

To confirm that K595 acts as a bona fide acceptor site for ubiquitin, site-directed mutagenesis was performed to construct a TIAM1 ubiquitylation mutant. K595 and three lysine residues that follow were mutated to arginine (Figure 5A) to suppress lysine acceptor promiscuity, which has been previously documented (Danielsen et al., 2011). MDCKII cells were engineered to allow doxycycline (Dox)-induced expression of either wild-type (WT) or 4x595R mutant TIAM1 (4x595R). Both 4x595R and WT TIAM1 were clearly observed to colocalize with E-CADHERIN at cell-cell adhesions (Figures 5B and S5C). Furthermore, we found that following HGF treatment the 4x595R mutant TIAM1 was able to both interact with RAC along cell-cell adhesions (Figures 5C and S5D) and activate RAC similarly to WT TIAM1 (Figure S5E). Thus, 4x595R mutant TIAM1 functions similarly to WT TIAM1 in these settings. To investigate if the 4x595R mutant TIAM1 was however defective in ubiquitylation and degradation, expression of HA-tagged WT and 4x595R mutant TIAM1 were induced in MDCKII cells that were subsequently pretreated for 3 hr with MG132 and stimulated with HGF for 30 min. HA-tagged proteins were immunoprecipitated and probed for K48-linked ubiquitin. HGF treatment promoted K48-linked ubiquitylation of WT, but not of 4x595R mutant TIAM1 (Figures 5D and S5F). Furthermore, the Duolink PLA assay revealed that, whereas WT TIAM1 and K48-Ub interacted extensively in HGF-stimulated cells and this interaction was enriched along cell-cell adhesions (Figures 5E, top, 5F, and S5G, top), 4x595R TIAM1 and K48-Ub showed only limited interaction, which did not appear to be localized to cell-cell adhesions (Figures 5E, bottom, 5F, and S5G, bottom). Moreover, MDCK 4x595R cells showed significantly increased TIAM1 stability compared to MDCK WT cells (Figures 5G and S5H). These data demonstrate that mutation of the K595 ubiquitylation site on TIAM1 results in a considerable decrease of HUWE1-mediated K48-linked ubiquitylation along cell-cell adhesions and a resultant increase in overall protein stability.

Ubiquitylation of TIAM1 at Lysine 595 Regulates Cell-Cell Adhesion Disassembly, Scattering, and Invasion

To further investigate the connection between TIAM1 ubiquitylation and cell-cell adhesion disassembly and migration, we compared MDCK WT to MDCK 4x595R cells in their ability to disassemble their cell-cell junctions following HGF stimulation. In unstimulated MDCKII cells in which expression of either WT or 4x595R mutant TIAM1 was not induced (minus Dox, minus HGF), E-CADHERIN, β-CATENIN, and F-ACTIN were localized at cell-cell contacts forming a characteristic honeycomb pattern (Figures 6A and 6B, top row). Unstimulated MDCKII cells in which WT or 4x595R mutant TIAM1 expression had been induced (plus Dox, minus HGF) exhibited a phenotype similar to that of control cells (minus Dox, minus HGF) (Figures 6A and 6B, second row). Treatment of noninduced cells with HGF for 1 hr (minus Dox, plus HGF) resulted in disassembly of cell-cell junctions accompanied by a redistribution of junctional markers that acquired intracellular localization (Figures 6A and 6B, third row). HGF-stimulated and induced MDCK WT cells (plus Dox, plus HGF) behaved similarly to their noninduced counterparts (minus Dox, plus HGF; Figure 6A, fourth row, see white arrows) becoming less compact and disassembling their cell-cell junctions. In contrast, disassembly of cell-cell adhesions was markedly delayed in HGF-treated and induced MDCK 4x595R cells (plus Dox, plus HGF), which retained their junctional organization as visualized by E-CADHERIN, F-ACTIN, and β-CATENIN staining (Figure 6B, fourth row, see white arrows).

To extend these observations, we compared the scattering of MDCK WT and 4x595R cells. Induced MDCK WT cells scattered following HGF treatment for either 9 or 18 hr (Figures 6C and S6A), whereas induced 4x595R cells showed a substantial decrease in scattering at both time points (Figures 6D and S6B). Protein levels were measured to ensure equivalent

(B) Quantification of (A). Percentage of cell scattering was calculated by counting the percentage of cells with less than three cell-cell adhesions remaining in each colony. At least ten colonies were counted in each of three independent biological replicates. **p < 0.001 (unpaired t test).

(C) MDCKII, H1299, H358, and H522 cells were transfected with a nontargeting siRNA (Ctrl) or siRNAs targeting TIAM1 or HUWE1 alone or in combination and seeded in a modified Boyden chamber coated with collagen I to assay for invasion in the presence of 10 ng/ml HGF. After 1 day, invading cells were stained with crystal violet. Panels show representative images from one of at least three independent experiments. Scale bar, 150 μm.

(D–G) Crystal violet from (C) was extracted and absorbance measured at 600 nm. Relative invasion was determined for (D) MDCKII (E) H1299, (F) H358, and (G) H522 cells by relating optical density to a standard curve of the appropriate cells and normalizing this to total cell number for each condition and cell line. Mean values ±SE from three independent experiments. ****p < 0.0001 (unpaired t test).

See also Figure S4.

OPEN ACCESS
CellPress

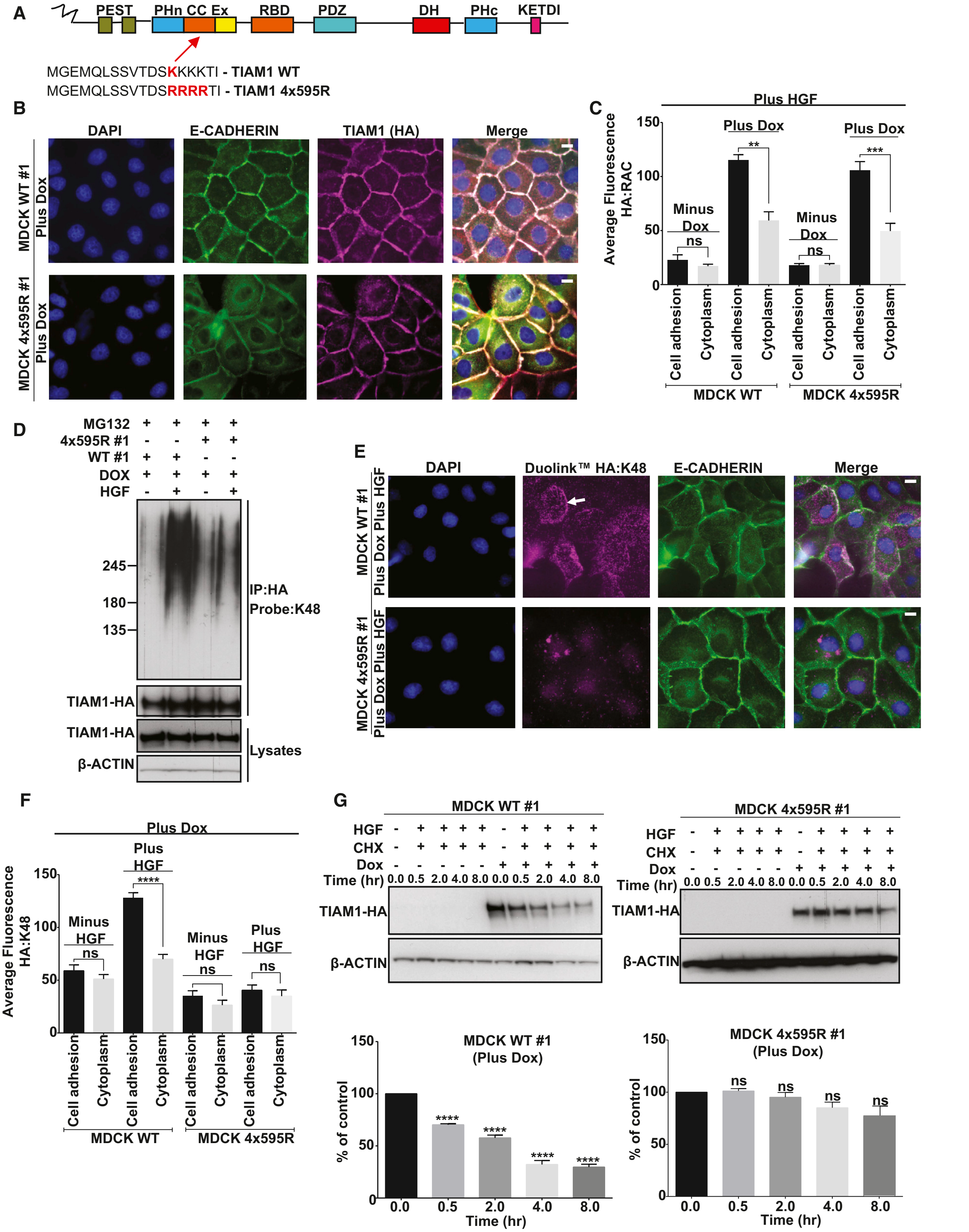

(legend on next page)

TIAM1 expression in doxycycline-induced MDCK WT and 4x595R cells (Figure S6C). Together, these results suggest that HUWE1-mediated ubiquitylation of TIAM1 on lysine 595 regulates HGF-induced cell-cell adhesion disassembly and scattering in MDCKII cells.

We next investigated whether the reduced scattering of cells expressing the 4x595R ubiquitylation defective TIAM1 mutant would lead to impaired cell invasion. Both short- (24 hr; Figures 6E and S6D) and long-term (120 hr; data not shown) invasion assays were performed as described above. Neither MDCK WT nor 4x595R cells invaded without HGF (data not shown). Moreover, noninduced MDCK WT and 4x595R cells readily invaded when stimulated with HGF (minus Dox; Figures 6E and S6D). Likewise, MDCK WT clones induced to express WT TIAM1 also invaded following HGF stimulation (plus Dox; Figures 6E and S6D). However, MDCK 4x595R cells induced to express the 4x595R mutant (plus Dox) invaded significantly less than noninduced 4x595R (minus Dox) or induced WT cells (plus Dox) stimulated with HGF (Figures 6E and S6D). Once again, to understand if the same mechanism is operating in lung carcinoma cells, we performed invasion assays in H1299, H358, and H522 lung cancer cell lines overexpressing either WT or 4x595R TIAM1. As for parental cells, no significant invasion of cells expressing either WT or 4x595R TIAM1 was seen without HGF stimulation (data not shown). However, both noninduced WT and 4x595R cells (minus Dox) as well as cell lines induced to express WT TIAM1 (plus Dox) readily invaded with HGF. In contrast, cells induced to express 4x595R TIAM1 (plus Dox) invaded significantly less than noninduced 4x595R (minus Dox) or induced WT cells (plus Dox) stimulated with HGF (Figures 6F–6H and S6E–S6G). Western blotting was performed to ensure equivalent expression of WT and 4x595R TIAM1 (Figures S6H–S6J). Based on this evidence, we conclude that aberrant HUWE1-mediated ubiquitylation of TIAM1 could contribute to the enhanced invasiveness of lung carcinoma cells.

The results above indicate that HUWE1 plays a role in cell-cell adhesion, motility, and invasion of epithelial cells in vitro by regulating TIAM1. To investigate this role in vivo, we used a zebrafish xenograft model. Fluorescently labeled H1299 cells were injected into the pericardial cavity of 2-day-old zebrafish embryos. Seventy-two hours postinjection, tumor cells were observed to have largely filled the approximately conical shaped cavity. Several cells were also observed to have disseminated outside the pericardial cavity, considered to have undergone local invasion (Figure S7A). H1299 cells treated with both a nontargeting (Ctrl) siRNA and TIAM1 targeting siRNAs also disseminated out of the pericardial cavity. However, dissemination of H1299 cells in which HUWE1 was depleted was significantly reduced (Figures 7A and 7B). Remarkably, the ability of H1299 cells to disseminate was completely restored when HUWE1 and TIAM1 were downregulated simultaneously (Figures 7A and 7B). To further examine the requirement of TIAM1 ubiquitylation for invasion in vivo, we injected H1299 cells expressing either WT or 4x595R TIAM1 into the pericardial cavity. All noninduced (minus Dox) H1299 cells and also H1299 cells expressing WT TIAM1 (plus Dox) disseminated away from the pericardial region similarly to parental H1299 cells (Ctrl), whereas cells expressing the nondegradable 4x595R TIAM1 (plus Dox) showed a strikingly diminished ability to disseminate (Figures 7C and 7D). Thus, HUWE1 appears essential for the invasion of lung carcinoma cells and this is entirely dependent on ubiquitylation of TIAM1.

HUWE1 Expression Is Negatively Correlated with TIAM1 in Squamous Cell Lung Carcinomas

To substantiate the clinical relevance of the above findings in lung carcinoma, we obtained tissue microarrays containing tumor samples from stage I and stage II squamous cell lung carcinoma patients. These were stained using immunohistochemistry (IHC) with specific antibodies recognizing HUWE1, c-MET, and TIAM1. (c-MET expression has already been shown to correlate with poor patient outcome in lung cancer [Siegfried et al., 1997].) In this cohort, 94.3%, 79.8%, and 98.6% of patients were positive for c-MET, TIAM1, and HUWE1 expression, respectively. Example images of lung cancer specimens with low and high HUWE1, TIAM1, and c-MET expression are shown in Figure S7B. A positive and statistically significant correlation was observed between HUWE1 and c-MET (Spearman coefficient $r = 0.3953$, $p = 0.0011$, 95% confidence interval [CI] 0.1518–0.5935; Figure S7C), and, strikingly, a strong inverse correlation was observed between HUWE1 and TIAM1 expression (Spearman

Figure 5. TIAM1 Is Ubiquitylated at K595

(A) Schematic representation of TIAM1 with the lysine 595 ubiquitylation site highlighted in red.

(B) MDCKII cells inducibly expressing (Plus Dox) either WT HA-tagged TIAM1 (WT #1) or mutant HA-tagged 4x595R TIAM1 (4x595R #1) were seeded on glass coverslips for 48 hr, fixed, and stained by IF for E-CADHERIN, TIAM1-HA, and DAPI. Representative images from MDCK WT #1 and MDCK 4x595R #1 clones are shown. Scale bar, 10 μm.

(C) Quantification of RAC:TIAM1-HA Duolink signal generated by seeding MDCKII cells noninduced (Minus Dox) or inducibly expressing (Plus Dox) either WT TIAM1 (WT #1) or mutant 4x595R TIAM1 (4x595R #1) and stimulating with 10 ng/ml HGF for 30 min.

(D) MDCKII cells inducibly expressing (+ Dox) either WT HA-tagged TIAM1 (WT #1) or mutant HA-tagged 4x595R TIAM1 (4x595R #1) were treated, where indicated, with 10 ng/ml HGF, and protein was extracted at 30 min. HA-tagged TIAM1 was immunoprecipitated under denaturing conditions and probed with a K48-linkage specific antibody.

(E) MDCKII cells inducibly expressing (Plus Dox) either HA-tagged WT TIAM1 or 4x595R mutant TIAM1 were pretreated for 3 hr with 5 μM MG132 and treated with 20 ng/ml HGF for 1 hr and fixed, and the Duolink protocol was performed to monitor the extent and localization of K48 ubiquitylated TIAM1. White arrow indicates intercellular junctions. Scale bar, 10 μm.

(F) Quantification of Duolink signal in (E) and also in unstimulated cells (Minus HGF). Mean values ±SE ****$p < 0.0001$; ns, not significant (unpaired t test).

(G) MDCKII cells noninduced (− Dox) or inducibly expressing (+ Dox) either WT TIAM1 (WT #1) or mutant 4x595R TIAM1 (4x595R #1) were treated with 50 μg/ml cycloheximide (CHX) and HGF (10 ng/ml) for the indicated times after which protein was extracted. Lysates were immunoblotted for TIAM1-HA. Quantification of TIAM1 from three independent experiments. β-ACTIN was used as a loading control in (D) and (G). Mean values ±SE ****$p < 0.0001$; ns, not significant (unpaired t test).

See also Figure S5.

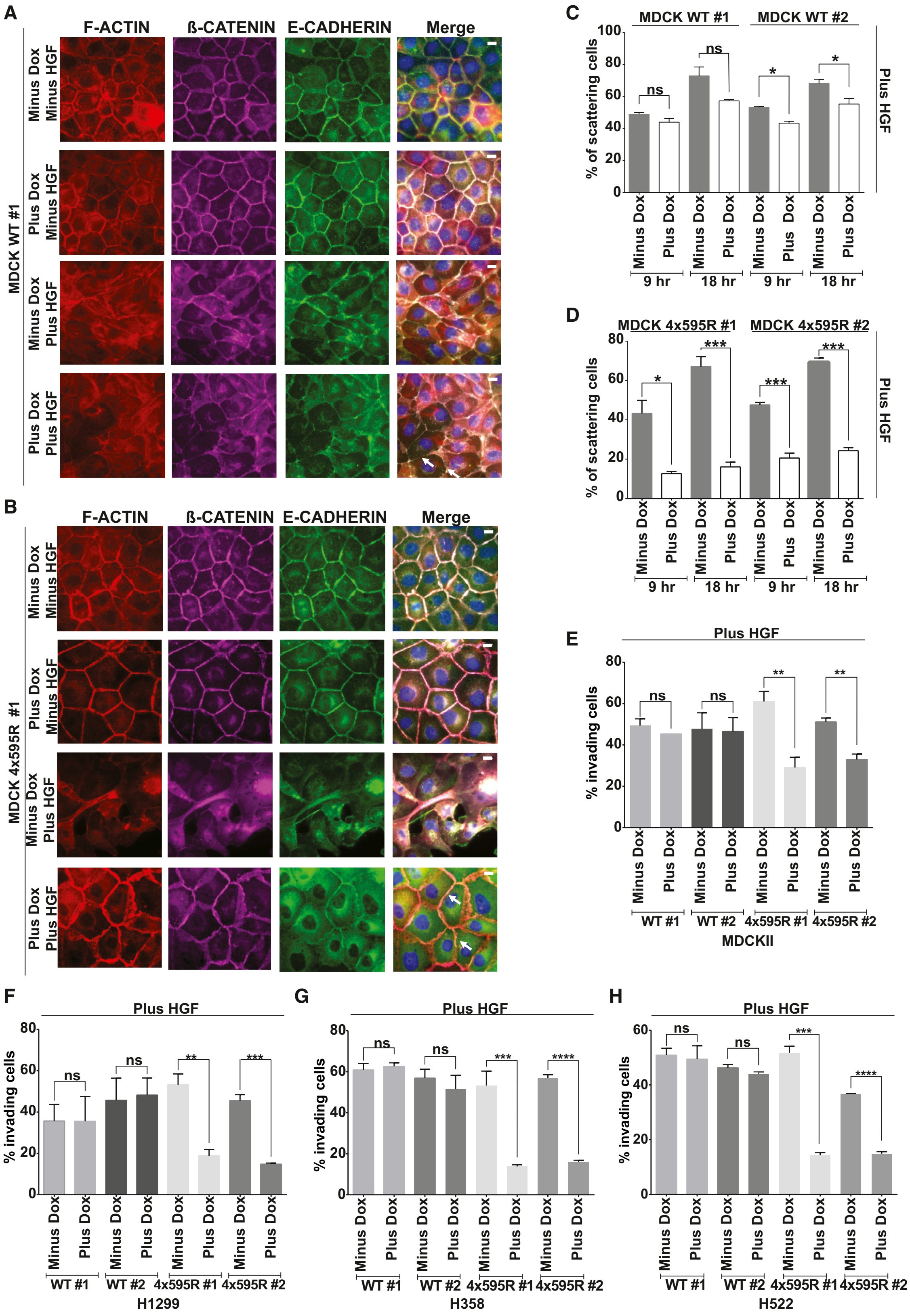

(legend on next page)

coefficient r = −0.4812, p < 0.0001, 95% CI −0.6582 to −0.2539; Figure 7E) and TIAM1 and c-MET expression (Spearman coefficient r = −0.3497, p = 0.0057, 95% CI −0.5582 to −0.09977; Figure S7D). Taken together, these results suggest that the spatiotemporal regulation of TIAM1 protein expression by the E3 ligase HUWE1 could be deregulated during lung tumorigenesis.

DISCUSSION

In this study, we have demonstrated a critical role for the E3 ligase HUWE1 in regulating cell-cell adhesion, cell motility, and invasion. We show that in epithelial cells, including lung carcinoma cells, stimulated with HGF, TIAM1—a critical regulator of cadherin-associated adhesion— is rapidly targeted for proteasome-dependent degradation via HUWE1-mediated ubiquitylation. Failure to degrade TIAM1 or depletion of HUWE1 in cells results in delayed adhesion disassembly and prevents the HGF-induced stimulation of migration and invasion. These findings reveal a molecular mechanism by which MET/HGF signaling, whose hyperactivation is associated with invasive growth of many neoplasms, stimulates epithelial cell motility and invasion.

Intriguingly, both oncogenic (Adhikary et al., 2005) and tumor suppressor (Inoue et al., 2013) roles have been attributed to HUWE1. HUWE1 is overexpressed in cancers of the lung, breast, colon, prostate, liver, pancreas, and thyroid but downregulated in stomach and uterine cancer as well as glioblastomas (Adhikary et al., 2005; Confalonieri et al., 2009). The conflicting reports surrounding HUWE1 function can in part be explained by the nature of its varying substrates, which are involved in a wide variety of processes including apoptosis (p53, [Adhikary et al., 2005]), DNA damage response (CDC6 [Hall et al., 2007]), and transcriptional regulation (e.g., p53, c-MYC [Adhikary et al., 2005; Inoue et al., 2013]). Our findings here suggest that effects of HUWE1 on cell-cell adhesion and invasion via regulating TIAM1 degradation would promote epithelial tumor progression.

Although both HUWE1 and TIAM1 are expressed in most tissues, it is possible that the mechanism described here for regulating migration is restricted to cells capable of forming cell-cell adhesions, because it is the pool of TIAM1 expressed at cell-cell junctions that appears to be preferentially degraded by HUWE1, allowing cells to move away from each other and become motile and invasive. It is therefore interesting that many solid tumors of epithelial origin demonstrate deregulation of both HUWE1 and TIAM1 protein expression. Indeed, we have shown here that an inverse correlation exists between TIAM1 and HUWE1 (and TIAM1 and c-MET) in squamous cell lung carcinoma. We speculate that, in neoplasms characterized by aberrant HGF/MET signaling, HUWE1 overexpression results in an increased turnover of TIAM1 at cell-cell adhesions, permitting junction disassembly and stimulating cell motility and invasion—steps vital under certain circumstances for the initiation of the metastatic cascade. Potentially, pharmacological agents that disrupt the HUWE1-TIAM1 interaction could be beneficial in decreasing the HGF/MET-driven metastatic dissemination of cancer cells.

The TIAM1-RAC signaling module is detected at various subcellular compartments and orchestrates multiple cellular processes. Spatiotemporal modulation of TIAM1 and RAC stability and/or activity is likely to be an important means of regulating TIAM1-RAC signaling. From our data, we infer that HUWE1-mediated ubiquitylation is an effective mechanism to achieve spatiotemporal modulation of TIAM1 protein levels and thereby regulate motility selectively. In our proposed model (Figure S7E), TIAM1 primarily at cell-cell adhesions is targeted for proteasomal degradation in cells stimulated with HGF by HUWE1-mediated ubiquitylation, sparing a significant cytoplasmic pool, which potentially could continue to promote cell growth, survival, and migration. Alternative mechanisms for controlling TIAM1 stability have emerged. Previously, we have shown that calpain cleavage of TIAM1 also regulates TIAM1 stability at adherens junctions but in the context of oncogenic SRC activation (Woodcock et al., 2009). Recently, it was shown that TIAM1 can also be targeted for ubiquitylation by SCF ubiquitin ligase containing β-TRCP (Magliozzi et al., 2014; Zhu et al., 2014). Intriguingly, we found that β-TRCP depletion did not impair HGF-induced scattering of MDCKII cells (data not shown), most likely due to targeting of a different pool of TIAM1 by β-TRCP compared to HUWE1. We have also recently shown ubiquitylation of RAC following HGF treatment of MDCKII cells mediated by the E3 ligase HACE (Castillo-Lluva et al., 2013). Thus, regulation of TIAM1-RAC signaling through ubiquitylation and proteasomal degradation appears pivotal to the rapid response of cells to extracellular signals.

In conclusion, we show that the E3 ligase HUWE1 is a regulator of cell-cell adhesion, migration, and invasion through the spatiotemporal modulation of the TIAM1 signaling network. In epithelial cells with activated c-MET or potentially simply overexpressing HUWE1, ubiquitylation of TIAM1 at cell-cell junctions

Figure 6. Ubiquitylation of TIAM1 at Lysine 595 Regulates Cell-Cell Adhesion Disassembly, Migration, and Invasion

(A and B) MDCKII cells noninduced (Minus Dox) or inducibly expressing (Plus Dox) either TIAM1 WT (WT #1) (A) or TIAM1 4x595R (4x595R #1) (B) were incubated for 1 hr in the presence (Plus HGF) or absence (Minus HGF) of 20 ng/ml HGF, fixed, and costained by IF for F-ACTIN, E-CADHERIN, β-CATENIN, and DAPI. Representative images from one of two independent MDCKII WT and 4x595R clones are shown. Scale bar, 10 μm.

(C and D) MDCKII cells noninduced (Minus Dox) or inducibly expressing (Plus Dox) either TIAM1 WT (WT #1 and WT #2) or TIAM1 4x595R (4x595R #1 and 4x595R #2) were incubated for the indicated times with 20 ng/ml HGF. Cell scattering was monitored by phase contrast microscopy and quantitation performed for two independent MDCK WT (C) and MDCK 4x595R (D) clones by calculating the percentage of cells with less than three cell-cell adhesions remaining in each colony. At least ten colonies were counted in each of three independent biological replicates. *p < 0.05, ***p < 0.0005; ns, not significant (unpaired t test).

(E–H) Noninduced (Minus Dox) MDCKII, H1299, H358, and H522 cells or the same cells inducibly expressing (Plus Dox) either TIAM1 WT or TIAM1 4x595R were seeded in a modified Boyden chamber to assay for invasion in the presence of 10 ng/ml HGF. Cells were left to invade for 1 day, fixed, and stained with crystal violet. Crystal violet was eluted, absorbance was measured at 600 nm, and relative invasion was determined for (E) MDCKII, (F) H1299, (G) H358, and (H) H522 by relating optical density to a standard curve of the appropriate cell type and then normalizing this to total cell number for each condition. Mean values ±SE **p < 0.001, ***p < 0.0005, ****p < 0.0001; ns, not significant (unpaired t test).

See also Figure S6.

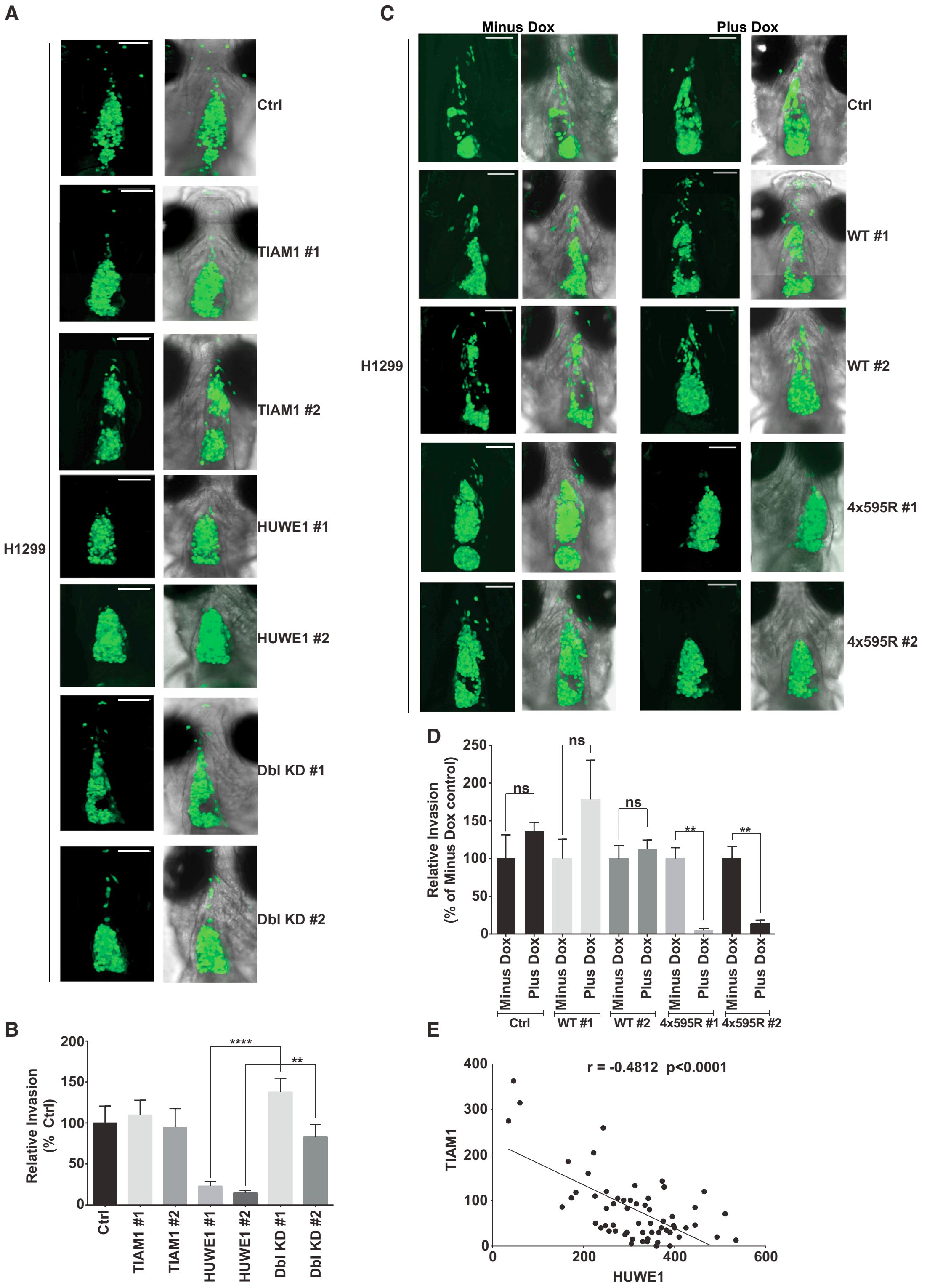

Figure 7. HUWE1-Mediated TIAM1 Degradation Controls Lung Carcinoma Cell Invasion In Vivo and HUWE1 and TIAM1 Are Negatively Correlated in Stage I and II Lung Carcinoma

(A) Green fluorescent H1299 cells were transfected with a nontargeting siRNA (Ctrl) or siRNAs targeting TIAM1 or HUWE1 alone or in combination and injected into the pericardial cavity of zebrafish embryos. Xenografted cells were imaged at 4 days postinjection (dpi). Right column of images show merge with bright field. Scale bar, 100 μm.

(legend continued on next page)

facilitates both cell-cell adhesion disassembly and invasion, thus overcoming the dissemination suppressing properties previously associated with the TIAM1/RAC signaling axis. Because ubiquitin-coupled degradation of numerous junctional proteins now appears critical in epithelial dedifferentiation and acquisition of a motile and invasive phenotype, pharmacological interventions targeting the ubiquitin-proteasome pathway could impact on carcinoma cell invasion and metastasis.

EXPERIMENTAL PROCEDURES

Constructs, antibodies, cell lines, and siRNA sequences are described in detail in the Supplemental Experimental Procedures.

Protein Analysis

Cells were lysed in lysis buffer (50 mM Tris-HCl [pH 7.5], 150 mM NaCl, 1% [v/v] Triton X-100, 10% [v/v] glycerol, 2 mM EDTA, 25 mM NaF, and 2 mM NaH_2PO4) containing protease and phosphatase inhibitor cocktails (Sigma) and proteins resolved by SDS-PAGE for western blotting. In protein turnover experiments, cells were treated with 50 μg/ml CHX for 0–8 hr prior to lysis. For proteasomal inhibition, cells were incubated for 3 hr with 5 μM MG132. Biochemical cell fractionation, immunoprecipitation, and GST pull-downs are described in the Supplemental Experimental Procedures. Rac activity assays were performed as previously described (Mack et al., 2012).

Ubiquitylation Experiments

In vivo and in vitro detection of protein ubiquitylation were performed as previously described (Choo and Zhang, 2009) with further details provided in the Supplemental Experimental Procedures.

Cell Growth

Cell growth was assessed using the Sulforhodamine B assay as previously described (Vichai and Kirtikara, 2006).

Immunofluorescence Microscopy

Cells were grown on glass coverslips and fixed with 100% ice-cold methanol or 3.7% formaldehyde. Postfixation, cells were washed with PBS and blocked with 3% BSA in 0.1% Triton/PBS for 1 hr before antibodies were added.

Halo Pulse-Chase Analysis

Halo pulse-chase analysis was carried out as previously described (Yamaguchi et al., 2009) with further details provided in the Supplemental Experimental Procedures.

Duolink PLA

Duolink PLA was performed using the Duolink II Red or Green Starter Kits (Sigma) following the manufacturer's instructions and is described in further detail in Supplemental Experimental Procedures.

Transwell Migration and Invasion Assays

Transwell migration and invasion experiments were performed as previously described (Marshall, 2011). Modifications are detailed in the Supplemental Experimental Procedures.

Xenograft Assays

Xenograft assay was performed as previously described (Chapman et al., 2014). Further details are provided in the Supplemental Experimental Procedures. Approval for this procedure was given by The University of Manchester Ethical Review Board and performed according to UK Home Office Regulations.

Immunohistochemical Analysis

The squamous cell tissue microarray included multiple tumor specimens from 83 patients. Immunopositivity for HUWE1, TIAM1, and c-MET protein expression in the TMAs was evaluated independently by two investigators (D.N. and L.V.) as described in Supplemental Experimental Procedures. Ethical approval for these studies was conferred under the MCRC Biobank Research Tissue Bank Ethics (07/H1003/161+5) from NRES Committee North West, Greater Manchester South Ethical.

Statistical Analysis

The specific statistical tests used are indicated in the figure legends alongside the p values and were carried out using GraphPad Prism version 6.0.

SUPPLEMENTAL INFORMATION

Supplemental Information includes Supplemental Experimental Procedures and seven figures and can be found with this article online at http://dx.doi.org/10.1016/j.celrep.2014.12.012.

AUTHOR CONTRIBUTITIONS

L.V. performed the majority of experiments, data analysis, and manuscript preparation. C.-T.T. generated preliminary data, identified the ubiquitylation site together with D.S., and generated Figures S1C, S3A, S3C, S3E, and S5B. A.C. of A.F.L.H.'s laboratory designed and performed the zebrafish experiments. D.N. is the pathologist who scored the TMA together with L.V. N.A.M. found and confirmed the interaction between TIAM1 and HUWE1. D.S. performed the MS analysis. R.B. was responsible for the construction of the lung TMA used in the study. A.M. was the grant holder and principal investigator who supervised the study and manuscript preparation and made intellectual contributions throughout.

ACKNOWLEDGMENTS

This work was supported by AICR (grant number 12-0037), MRC (grant number MR/L007495/1), and Cancer Research UK (grant number C5759/A12328) to A.M., by Cancer Research UK (grant number C11876/A12724) to A.F.L.H. and also by a Wellcome Trust Institutional Strategic Support Fund (ISSF) award (097820/Z/11/A) to The University of Manchester. We thank John Brognard for the lung cancer cell lines utilized in this work; G. Ashton for immunohistochemical staining; S. Bagley and K. Zeng for help with microscopy and image analysis; Caroline Wilkinson and John Brognard as well as members of the Cell Signalling Group for helpful discussions and critical reading of the manuscript. Research TMA samples were obtained from the Manchester Cancer Research Centre (MCRC) Biobank, UK.

Received: May 16, 2014
Revised: October 22, 2014
Accepted: December 5, 2014
Published: December 24, 2014

(B) Quantitation of invasion depicted in (A). Invasion was normalized to Ctrl. Mean values ±SE from three independent experiments. $^{**}p < 0.01$, $^{****}p < 0.0001$ (Kruskal-Wallis one-way analysis of variance).

(C) Parental green fluorescent H1299 cells (Ctrl), noninduced (Minus Dox), or inducibly expressing (Plus Dox) either WT TIAM1 (WT #1 and WT #2) or 4x595R TIAM1 (4x595R #1 and 4x595R #2) were injected into the pericardial cavity of zebrafish embryos. Xenografted cells were imaged at 4 dpi. Second and fourth column of images show merge with bright field. Scale bar, 100 μm.

(D) Quantitation of invasion depicted in (C). In each case, invasion was normalized to the corresponding Minus Dox. Mean values ±SE from three independent experiments. $^{**}p < 0.01$ (Kruskal-Wallis one-way analysis of variance).

(E) Scatterplot depicting the statistically significant Spearman's negative correlation between HUWE1 and TIAM1 in corresponding tissue sections.

See also Figure S7.

REFERENCES

Adams, H.C., 3rd, Chen, R., Liu, Z., and Whitehead, I.P. (2010). Regulation of breast cancer cell motility by T-cell lymphoma invasion and metastasis-inducing protein. Breast Cancer Res. *12*, R69.

Adhikary, S., Marinoni, F., Hock, A., Hulleman, E., Popov, N., Beier, R., Bernard, S., Quarto, M., Capra, M., Goettig, S., et al. (2005). The ubiquitin ligase HectH9 regulates transcriptional activation by Myc and is essential for tumor cell proliferation. Cell *123*, 409–421.

Bourguignon, L.Y., Zhu, H., Shao, L., and Chen, Y.W. (2000). Ankyrin-Tiam1 interaction promotes Rac1 signaling and metastatic breast tumor cell invasion and migration. J. Cell Biol. *150*, 177–191.

Castillo-Lluva, S., Tan, C.T., Daugaard, M., Sorensen, P.H., and Malliri, A. (2013). The tumour suppressor HACE1 controls cell migration by regulating Rac1 degradation. Oncogene *32*, 1735–1742.

Chapman, A., Fernandez del Ama, L., Ferguson, J., Kamarashev, J., Wellbrock, C., and Hurlstone, A. (2014). Heterogeneous tumor subpopulations cooperate to drive invasion. Cell Rep. *8*, 688–695.

Chau, V., Tobias, J.W., Bachmair, A., Marriott, D., Ecker, D.J., Gonda, D.K., and Varshavsky, A. (1989). A multiubiquitin chain is confined to specific lysine in a targeted short-lived protein. Science *243*, 1576–1583.

Chen, D., Kon, N., Li, M., Zhang, W., Qin, J., and Gu, W. (2005). ARF-BP1/Mule is a critical mediator of the ARF tumor suppressor. Cell *121*, 1071–1083.

Choo, Y.S., and Zhang, Z. (2009). Detection of protein ubiquitination. J. Vis. Exp. http://dx.doi.org/10.3791/1293

Confalonieri, S., Quarto, M., Goisis, G., Nuciforo, P., Donzelli, M., Jodice, G., Pelosi, G., Viale, G., Pece, S., and Di Fiore, P.P. (2009). Alterations of ubiquitin ligases in human cancer and their association with the natural history of the tumor. Oncogene *28*, 2959–2968.

Danielsen, J.M., Sylvestersen, K.B., Bekker-Jensen, S., Szklarczyk, D., Poulsen, J.W., Horn, H., Jensen, L.J., Mailand, N., and Nielsen, M.L. (2011). Mass spectrometric analysis of lysine ubiquitylation reveals promiscuity at site level. Mol. Cell. Proteomics *10*, 003590.

Fujita, Y., Krause, G., Scheffner, M., Zechner, D., Leddy, H.E., Behrens, J., Sommer, T., and Birchmeier, W. (2002). Hakai, a c-Cbl-like protein, ubiquitinates and induces endocytosis of the E-cadherin complex. Nat. Cell Biol. *4*, 222–231.

Habets, G.G., van der Kammen, R.A., Stam, J.C., Michiels, F., and Collard, J.G. (1995). Sequence of the human invasion-inducing TIAM1 gene, its conservation in evolution and its expression in tumor cell lines of different tissue origin. Oncogene *10*, 1371–1376.

Hall, J.R., Kow, E., Nevis, K.R., Lu, C.K., Luce, K.S., Zhong, Q., and Cook, J.G. (2007). Cdc6 stability is regulated by the Huwe1 ubiquitin ligase after DNA damage. Mol. Biol. Cell *18*, 3340–3350.

Hordijk, P.L., ten Klooster, J.P., van der Kammen, R.A., Michiels, F., Oomen, L.C., and Collard, J.G. (1997). Inhibition of invasion of epithelial cells by Tiam1-Rac signaling. Science *278*, 1464–1466.

Inoue, S., Hao, Z., Elia, A.J., Cescon, D., Zhou, L., Silvester, J., Snow, B., Harris, I.S., Sasaki, M., Li, W.Y., et al. (2013). Mule/Huwe1/Arf-BP1 suppresses Ras-driven tumorigenesis by preventing c-Myc/Miz1-mediated down-regulation of p21 and p15. Genes Dev. *27*, 1101–1114.

Kon, N., Zhong, J., Qiang, L., Accili, D., and Gu, W. (2012). Inactivation of arf-bp1 induces p53 activation and diabetic phenotypes in mice. J. Biol. Chem. *287*, 5102–5111.

Mack, N.A., Porter, A.P., Whalley, H.J., Schwarz, J.P., Jones, R.C., Khaja, A.S., Bjartell, A., Anderson, K.I., and Malliri, A. (2012). β2-syntrophin and Par-3 promote an apicobasal Rac activity gradient at cell-cell junctions by differentially regulating Tiam1 activity. Nat. Cell Biol. *14*, 1169–1180.

Magliozzi, R., Kim, J., Low, T.Y., Heck, A.J., and Guardavaccaro, D. (2014). Degradation of Tiam1 by casein kinase 1 and the SCFβTrCP ubiquitin ligase controls the duration of mTOR-S6K signaling. J. Biol. Chem. *289*, 27400–27409.

Malliri, A., van der Kammen, R.A., Clark, K., van der Valk, M., Michiels, F., and Collard, J.G. (2002). Mice deficient in the Rac activator Tiam1 are resistant to Ras-induced skin tumours. Nature *417*, 867–871.

Malliri, A., van Es, S., Huveneers, S., and Collard, J.G. (2004). The Rac exchange factor Tiam1 is required for the establishment and maintenance of cadherin-based adhesions. J. Biol. Chem. *279*, 30092–30098.

Marshall, J. (2011). Transwell(®) invasion assays. Methods Mol. Biol. *769*, 97–110.

Michiels, F., Habets, G.G., Stam, J.C., van der Kammen, R.A., and Collard, J.G. (1995). A role for Rac in Tiam1-induced membrane ruffling and invasion. Nature *375*, 338–340.

Minard, M.E., Herynk, M.H., Collard, J.G., and Gallick, G.E. (2005). The guanine nucleotide exchange factor Tiam1 increases colon carcinoma growth at metastatic sites in an orthotopic nude mouse model. Oncogene *24*, 2568–2573.

Rygiel, T.P., Mertens, A.E., Strumane, K., van der Kammen, R., and Collard, J.G. (2008). The Rac activator Tiam1 prevents keratinocyte apoptosis by controlling ROS-mediated ERK phosphorylation. J. Cell Sci. *121*, 1183–1192.

Sander, E.E., van Delft, S., ten Klooster, J.P., Reid, T., van der Kammen, R.A., Michiels, F., and Collard, J.G. (1998). Matrix-dependent Tiam1/Rac signaling in epithelial cells promotes either cell-cell adhesion or cell migration and is regulated by phosphatidylinositol 3-kinase. J. Cell Biol. *143*, 1385–1398.

Siegfried, J.M., Weissfeld, L.A., Singh-Kaw, P., Weyant, R.J., Testa, J.R., and Landreneau, R.J. (1997). Association of immunoreactive hepatocyte growth factor with poor survival in resectable non-small cell lung cancer. Cancer Res. *57*, 433–439.

Sleeman, J., and Steeg, P.S. (2010). Cancer metastasis as a therapeutic target. Eur. J. Cancer *46*, 1177–1180.

Stebel, A., Brachetti, C., Kunkel, M., Schmidt, M., and Fritz, G. (2009). Progression of breast tumors is accompanied by a decrease in expression of the Rho guanine exchange factor Tiam1. Oncol. Rep. *21*, 217–222.

Uehara, Y., and Kitamura, N. (1992). Expression of a human hepatocyte growth factor/scatter factor cDNA in MDCK epithelial cells influences cell morphology, motility, and anchorage-independent growth. J. Cell Biol. *117*, 889–894.

Vichai, V., and Kirtikara, K. (2006). Sulforhodamine B colorimetric assay for cytotoxicity screening. Nat. Protoc. *1*, 1112–1116.

Wang, H.M., and Wang, J. (2012). Expression of Tiam1 in lung cancer and its clinical significance. Asian Pac. J. Cancer Prev. *13*, 613–615.

Woodcock, S.A., Rooney, C., Liontos, M., Connolly, Y., Zoumpourlis, V., Whetton, A.D., Gorgoulis, V.G., and Malliri, A. (2009). SRC-induced disassembly of adherens junctions requires localized phosphorylation and degradation of the rac activator tiam1. Mol. Cell *33*, 639–653.

Yamaguchi, K., Inoue, S., Ohara, O., and Nagase, T. (2009). Pulse-chase experiment for the analysis of protein stability in cultured mammalian cells by covalent fluorescent labeling of fusion proteins. Methods Mol. Biol. *577*, 121–131.

Zhao, X., D' Arca, D., Lim, W.K., Brahmachary, M., Carro, M.S., Ludwig, T., Cardo, C.C., Guillemot, F., Aldape, K., Califano, A., et al. (2009). The N-Myc-DLL3 cascade is suppressed by the ubiquitin ligase Huwe1 to inhibit proliferation and promote neurogenesis in the developing brain. Dev. Cell *17*, 210–221.

Zhu, G., Fan, Z., Ding, M., Mu, L., Liang, J., Ding, Y., Fu, Y., Huang, B., and Wu, W. (2014). DNA damage induces the accumulation of Tiam1 by blocking β-TrCP-dependent degradation. J. Biol. Chem. *289*, 15482–15494.

Cell Reports
Supplemental Information

HUWE1 Ubiquitylates and Degrades the Rac Activator TIAM1 Promoting Cell-Cell Adhesion Disassembly, Migration, and Invasion

Lynsey Vaughan, Chong-Teik Tan, Anna Chapman, Daisuke Nonaka, Natalie Mack, Duncan Smith, Richard Booton, Adam F.L. Hurlstone, and Angeliki Malliri

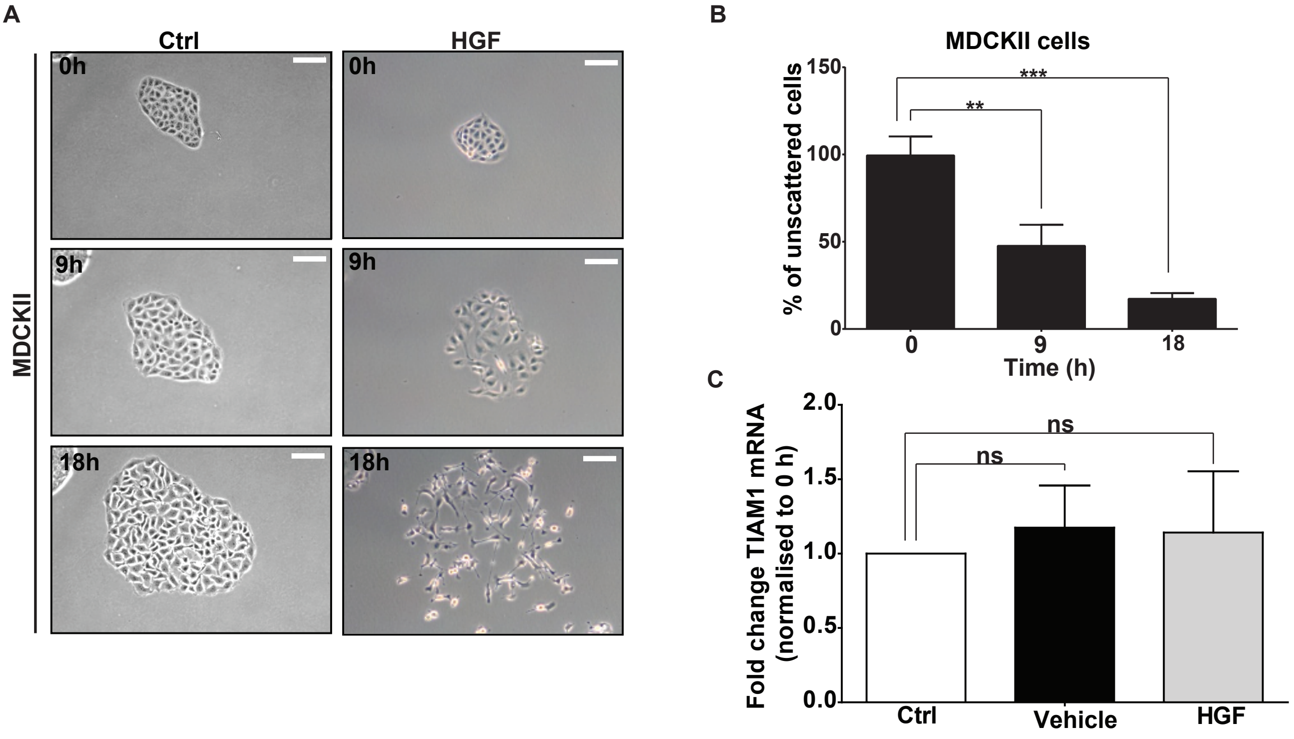
A
Ctrl
HGF
0h
0h
9h
9h
18h
18h
MDCKII
B
MDCKII cells
% of unscattered cells
150
100
50
0
***
**
0
9
18
Time (h)
C
Fold change TIAM1 mRNA (normalised to 0 h)
2.0
1.5
1.0
0.5
0.0
ns
ns
Ctrl
Vehicle
HGF

Figure S1.

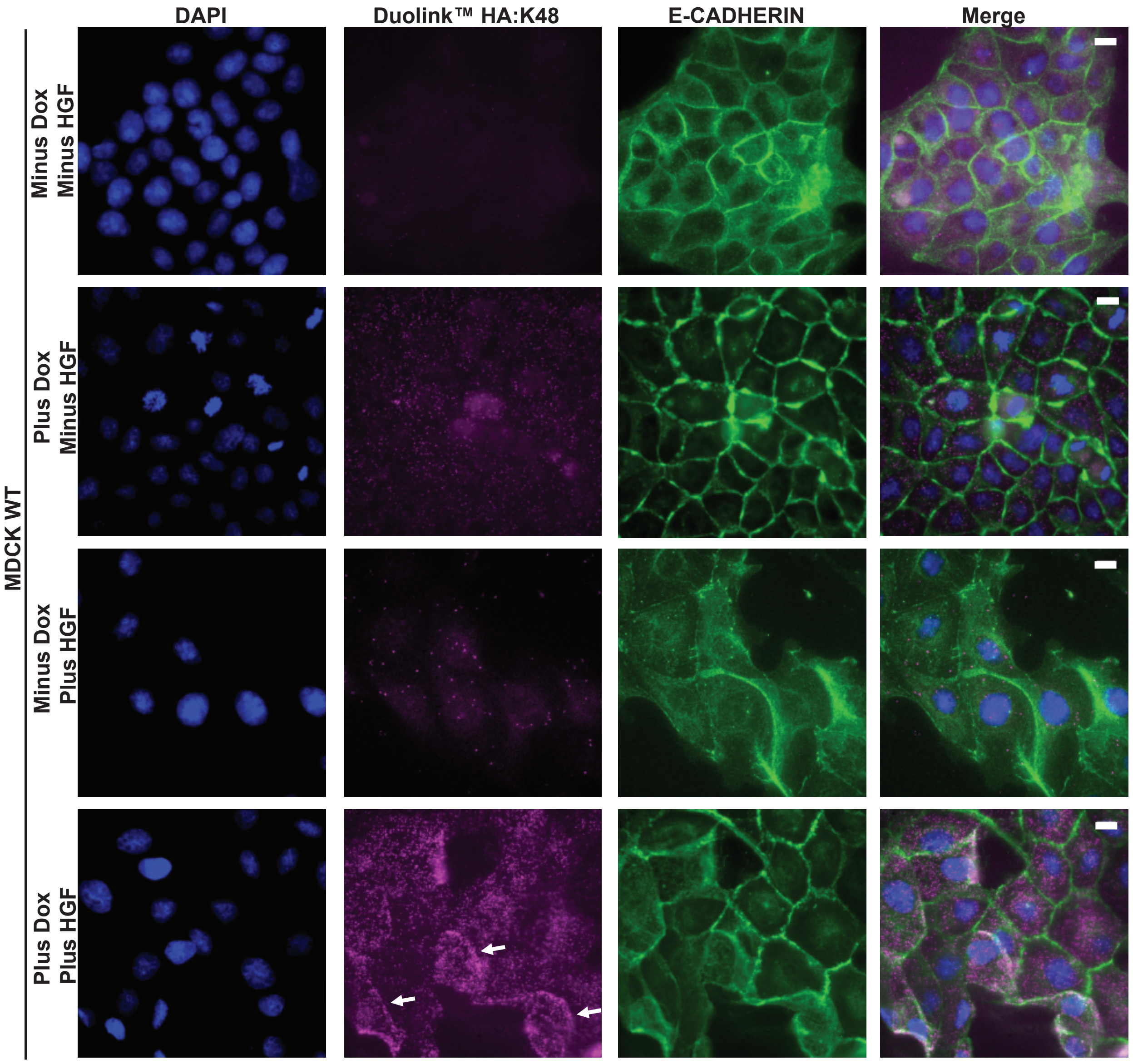
DAPI
Duolink™ HA:K48
E-CADHERIN
Merge
MDCK WT
Minus Dox
Minus HGF
Plus Dox
Minus HGF
Minus Dox
Plus HGF
Plus Dox
Plus HGF

Figure S2.

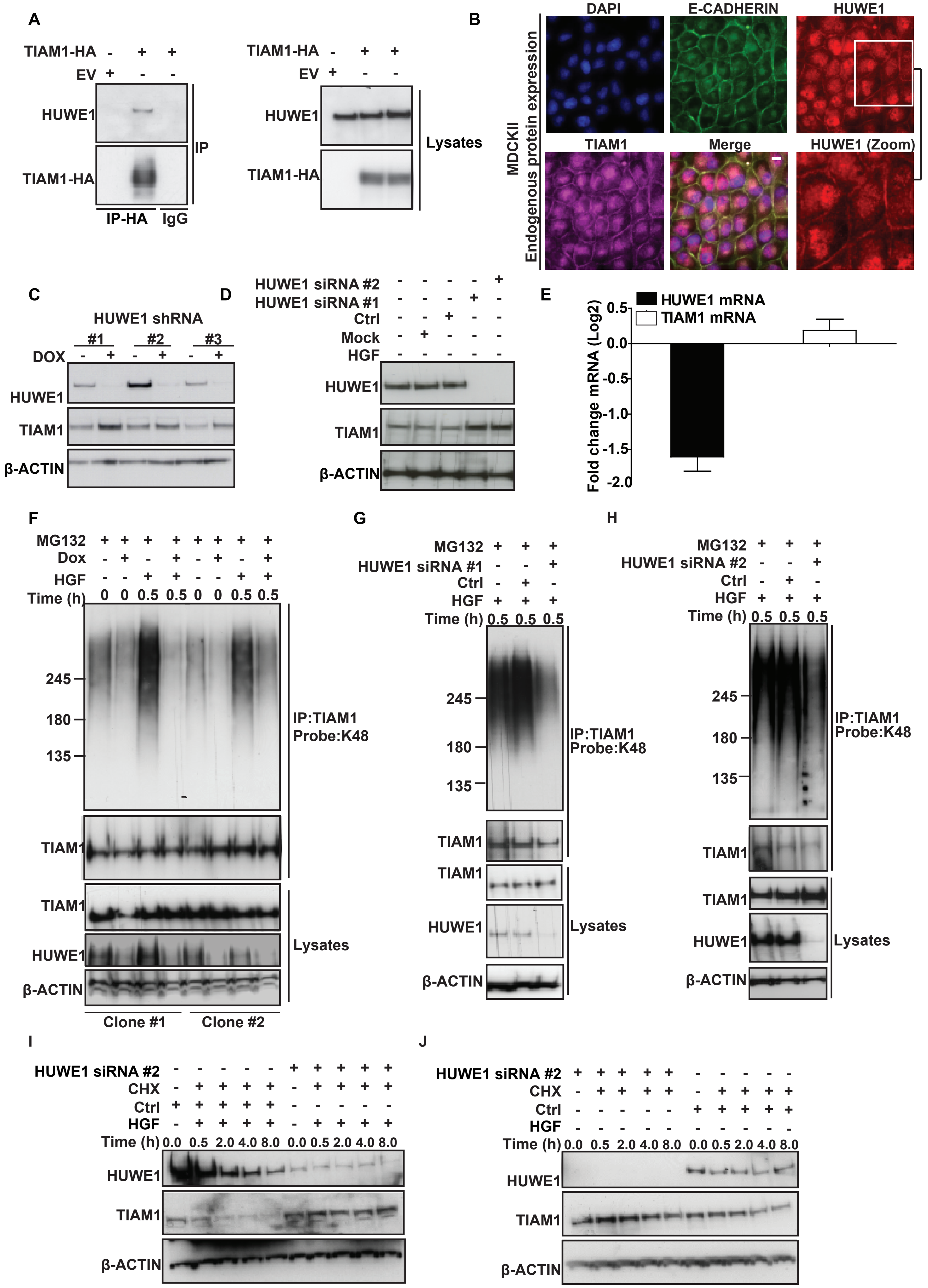
A
TIAM1-HA - + +
EV + - -
HUWE1
TIAM1-HA
IP
IP-HA IgG
TIAM1-HA - + +
EV + - -
HUWE1
TIAM1-HA
Lysates
B
MDCKII Endogenous protein expression
DAPI
E-CADHERIN
HUWE1
TIAM1
Merge
HUWE1 (Zoom)
C
HUWE1 shRNA
#1 #2 #3
DOX - + - + - +
HUWE1
TIAM1
β-ACTIN
D
HUWE1 siRNA #2 - - - - +
HUWE1 siRNA #1 - - - + -
Ctrl - - + - -
Mock - + - - -
HGF - - - - -
HUWE1
TIAM1
β-ACTIN
E
HUWE1 mRNA
TIAM1 mRNA
Fold change mRNA (Log2)
0.5
0.0
-0.5
-1.0
-1.5
-2.0
F
MG132 + + + + + + + +
Dox - + - + - + - +
HGF - - + + - - + +
Time (h) 0 0 0.5 0.5 0 0 0.5 0.5
245
180
135
IP:TIAM1
Probe:K48
TIAM1
TIAM1
HUWE1
β-ACTIN
Lysates
Clone #1
Clone #2
G
MG132 + + +
HUWE1 siRNA #1 - - +
Ctrl - + -
HGF + + +
Time (h) 0.5 0.5 0.5
245
180
135
IP:TIAM1
Probe:K48
TIAM1
TIAM1
HUWE1
β-ACTIN
Lysates
H
MG132 + + +
HUWE1 siRNA #2 - - +
Ctrl - + -
HGF + + +
Time (h) 0.5 0.5 0.5
245
180
135
IP:TIAM1
Probe:K48
TIAM1
TIAM1
HUWE1
β-ACTIN
Lysates
I
HUWE1 siRNA #2 - - - - - + + + + +
CHX - + + + + - + + + +
Ctrl + + + + + - - - - -
HGF - + + + + - + + + +
Time (h) 0.0 0.5 2.0 4.0 8.0 0.0 0.5 2.0 4.0 8.0
HUWE1
TIAM1
β-ACTIN
J
HUWE1 siRNA #2 + + + + + - - - - -
CHX - + + + + - + + + +
Ctrl - - - - - + + + + +
HGF - - - - - - - - - -
Time (h) 0.0 0.5 2.0 4.0 8.0 0.0 0.5 2.0 4.0 8.0
HUWE1
TIAM1
β-ACTIN

Figure S3.

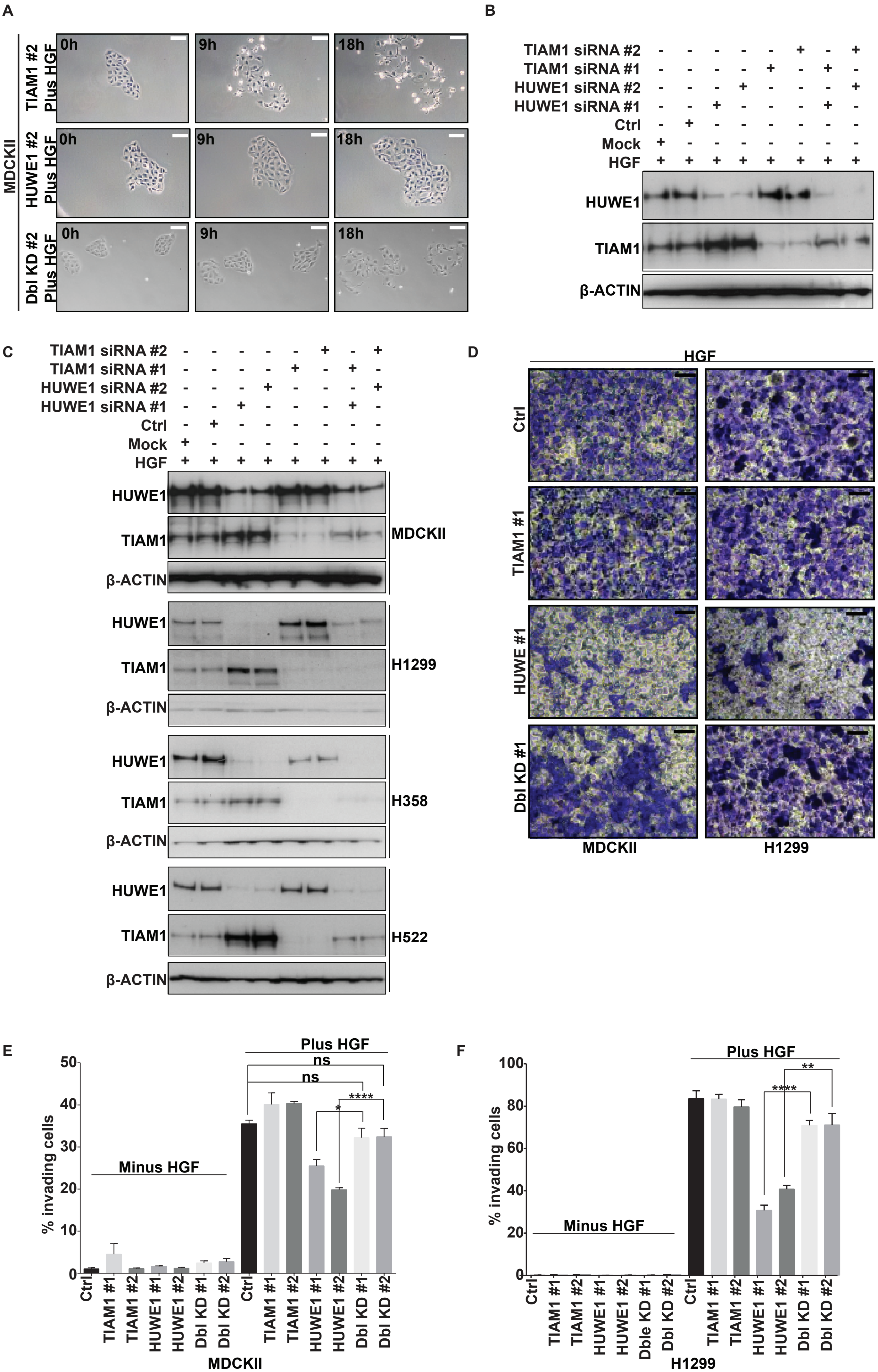
A
MDCKII
TIAM1 #2 Plus HGF
HUWE1 #2 Plus HGF
Dbl KD #2 Plus HGF
0h
9h
18h
B
TIAM1 siRNA #2
TIAM1 siRNA #1
HUWE1 siRNA #2
HUWE1 siRNA #1
Ctrl
Mock
HGF
HUWE1
TIAM1
β-ACTIN
C
TIAM1 siRNA #2
TIAM1 siRNA #1
HUWE1 siRNA #2
HUWE1 siRNA #1
Ctrl
Mock
HGF
HUWE1
TIAM1
β-ACTIN
MDCKII
H1299
H358
H522
D
HGF
Ctrl
TIAM1 #1
HUWE #1
Dbl KD #1
MDCKII
H1299
E
Plus HGF
ns
ns
****
*
Minus HGF
% invading cells
0
10
20
30
40
50
Ctrl
TIAM1 #1
TIAM1 #2
HUWE1 #1
HUWE1 #2
Dbl KD #1
Dbl KD #2
MDCKII
F
Plus HGF
**
****
Minus HGF
% invading cells
0
20
40
60
80
100
Ctrl
TIAM1 #1
TIAM1 #2
HUWE1 #1
HUWE1 #2
Dble KD #1
Dbl KD #2
H1299

Figure S4.

A

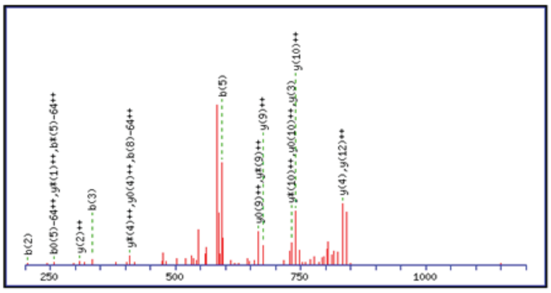


```
Monoisotopic mass of neutral peptide Mr(calc): 1811.8393
Variable modifications:
M1      : Oxidation (M), with neutral losses 0.0000(shown in table), 63.9983
Q5      : Deamidated (NQ)
K13     : LeuArgGlyGly (K)
```

#	b	b++	b*	b*++	b0	b0++	Seq.	y	y++	y*
1	148.0427	74.5250					M			
2	*205.0641*	103.0357					G	1665.8112	833.4093	1648.7847
3	*334.1067*	167.5570			316.0962	158.5517	E	1608.7898	804.8985	1591.7632
4	465.1472	233.0772			447.1367	224.0720	M	1479.7472	740.3772	1462.7206
5	*594.1898*	297.5985	577.1633	289.0853	576.1792	288.5933	Q	1348.7067	674.8570	1331.6801
6	707.2739	354.1406	690.2473	345.6273	689.2633	345.1353	L	1219.6641	610.3357	1202.6375
7	794.3059	397.6566	777.2794	389.1433	776.2953	388.6513	S	1106.5800	553.7937	1089.5535
8	881.3379	441.1726	864.3114	432.6593	863.3274	432.1673	S	1019.5480	510.2776	1002.5215
9	980.4063	490.7068	963.3798	482.1935	962.3958	481.7015	V	932.5160	466.7616	915.4894
10	1081.4540	541.2307	1064.4275	532.7174	1063.4435	532.2254	T	**833.4476**	417.2274	816.4210
11	1196.4810	598.7441	1179.4544	590.2308	1178.4704	589.7388	D	**732.3999**	366.7036	715.3733
12	1283.5130	642.2601	1266.4864	633.7469	1265.5024	633.2549	S	617.3729	309.1901	600.3464
13							K	530.3409	265.6741	513.3144

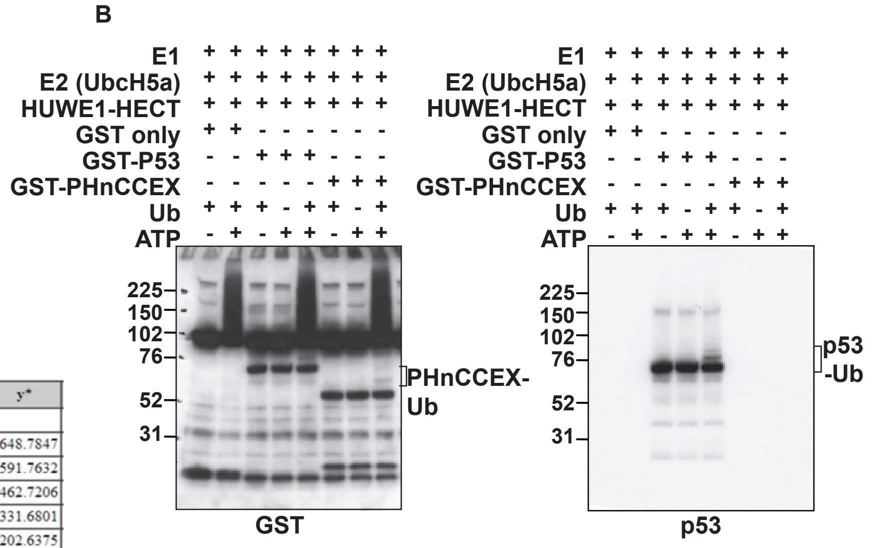

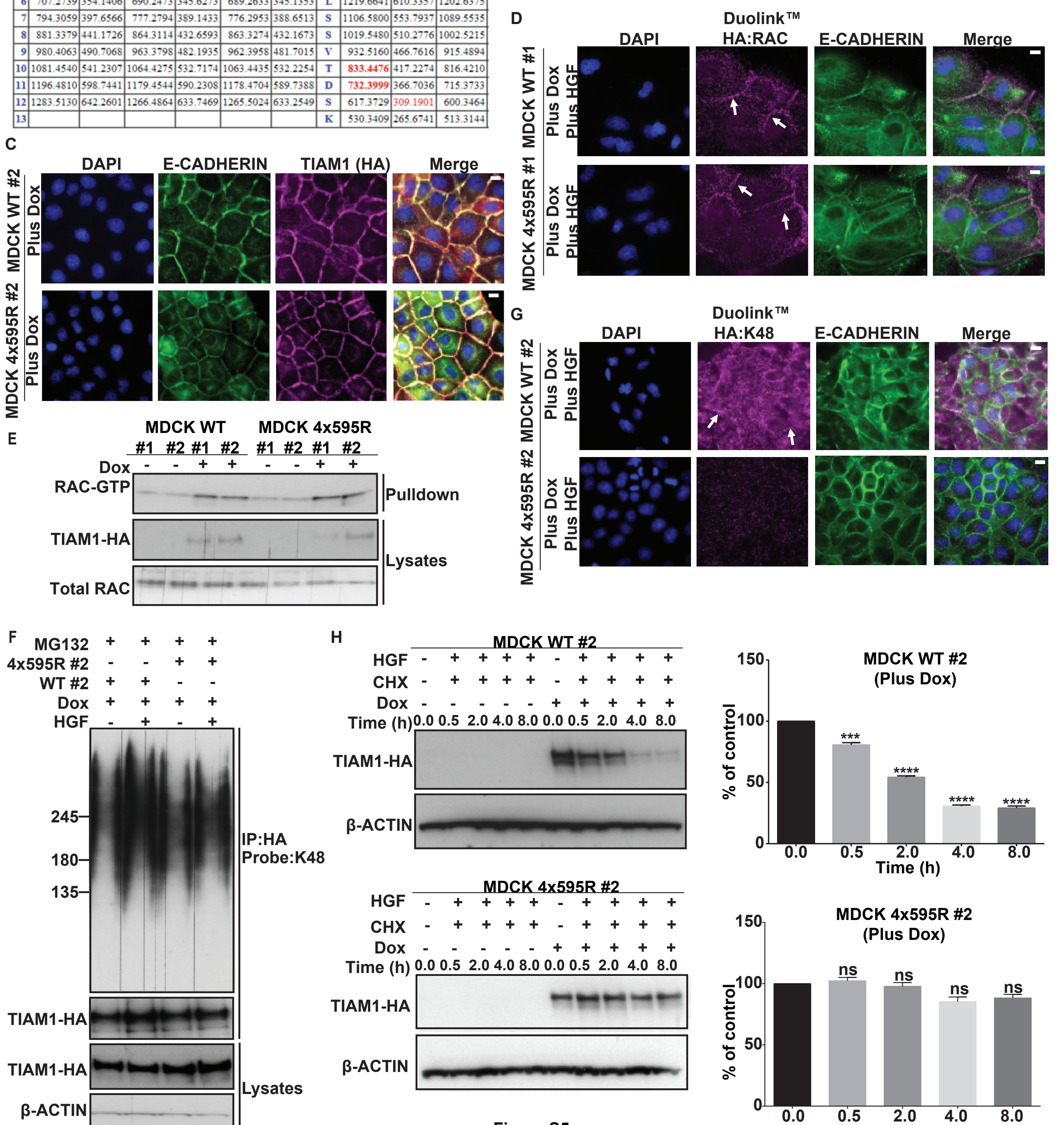

Figure S5.

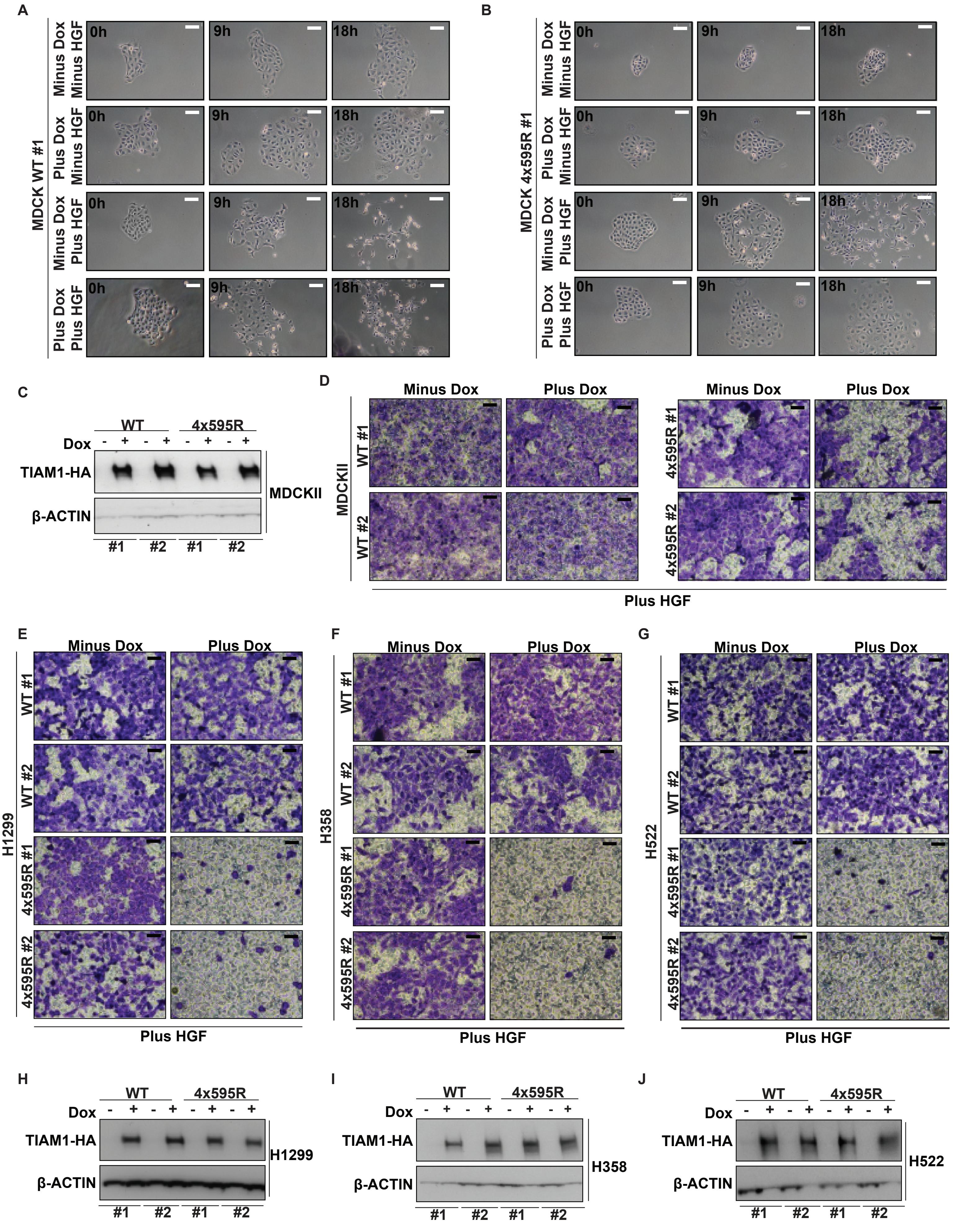

A
B
MDCK WT #1
MDCK 4x595R #1
Minus Dox Minus HGF
Plus Dox Minus HGF
Minus Dox Plus HGF
Plus Dox Plus HGF
0h
9h
18h
C
WT
4x595R
Dox
TIAM1-HA
β-ACTIN
MDCKII
#1
#2
D
Minus Dox
Plus Dox
MDCKII
WT #1
WT #2
4x595R #1
4x595R #2
Plus HGF
E
F
G
H1299
H358
H522
H
I
J

Figure S6.

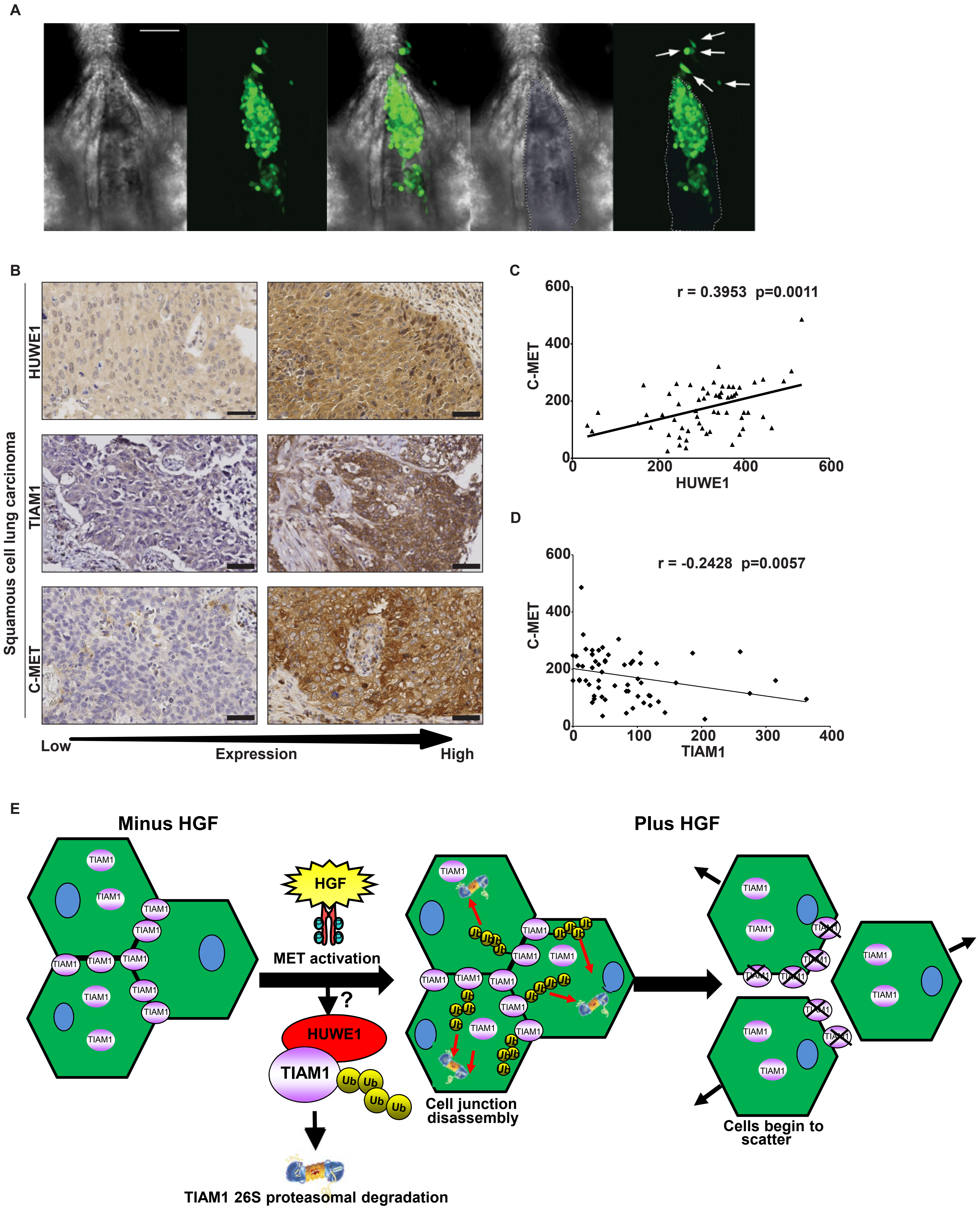
A
B
HUWE1
TIAM1
C-MET
Squamous cell lung carcinoma
Low
Expression
High
C
r = 0.3953 p=0.0011
C-MET
HUWE1
600
400
200
0
D
r = -0.2428 p=0.0057
C-MET
TIAM1
100
300
E
Minus HGF
Plus HGF
TIAM1
HGF
MET activation
?
HUWE1
Ub
TIAM1 26S proteasomal degradation
Cell junction disassembly
Cells begin to scatter

Figure S7.

SUPPLEMENTAL FIGURE LEGENDS

Figure S1, related to Figure 1. HGF stimulation induces scattering of epithelial MDCKII cells. (A) MDCKII cells were treated in the presence (HGF) or absence (Ctrl) of 10 ng/ml HGF for the indicated times and resultant cell scattering monitored by phase contrast microscopy. Scale bar = 50 µm. (B) Quantification of (A) from three independent experiments is shown. Results are mean values ± SE. ** signifies $p<0.001$ and *** $p<0.0005$ (Unpaired t test). (C) MDCKII cells were treated with 10 ng/ml HGF (HGF), 0.1% BSA (vehicle) or left untreated (Ctrl) for 6 h and relative TIAM1 mRNA levels determined by quantitative RT-PCR. Data were normalised to the hypoxanthine phosphoribosyl transferase 1 (HPRT1) housekeeping gene and were obtained from three biological replicates. Error bars indicate ± SD, ns = not significant.

Figure S2, related to Figure 2. HGF stimulation induces ubiquitylation of TIAM1. MDCKII cells inducibly expressing HA-tagged TIAM1 WT were left untreated (Minus Dox, Minus HGF), pre-treated with 1 µg/ml Doxycycline (Dox) for 24 h to induce exogenous TIAM1-HA expression (Plus Dox, Minus HGF), treated with 10 ng/ml HGF alone (Minus Dox, Plus HGF) or with both Dox and HGF (Plus Dox, Plus HGF) and fixed. The Duolink protocol was then performed to monitor the extent and localisation of K48 ubiquitylated TIAM1. White arrows indicate intercellular junctions. Scale bar = 10 µm.

Figure S3, related to Figure 3. TIAM1 is ubiquitylated by HUWE1. (A) HEK293T cells were transfected with an empty vector (EV, pCDNA3-HA) or an expression vector containing HA-tagged wild-type TIAM1 (TIAM1-HA). 24 hours post-transfection, protein extracts were immunoprecipitated with a mouse HA-specific antibody or non-specific mouse IgG as a negative control and lysates probed for endogenous HUWE1. (B) MDCKII cells were stained by immunofluorescence for HUWE1, E-CADHERIN, TIAM1 and DAPI. Panels show representative images from one of three independent experiments. Scale bar = 10 µm. (C) MDCKII single cell clones were generated allowing inducible knockdown of HUWE1 (shRNA clones #1 - #3). Representative immunoblots of HUWE1 knockdown and TIAM1 protein levels are shown. (D) MDCKII cells were transfected with 2 different siRNAs (#1 and #2) or a non-targeting control siRNA (Ctrl). Cells were lysed, protein extracted and subsequently immunoblotted for TIAM1 and HUWE1 96 h post transfection. (E) HUWE1 was depleted in MDCKII

cells using shRNA and relative TIAM1 and HUWE1 mRNA expression levels determined by quantitative RT-PCR. Data were normalised to the house-keeping gene HPRT1. Results are mean values ± SD from four independent experiments. (F) Endogenous HUWE1 was inducibly depleted in 2 different HUWE1 shRNA knockdown clones and at 48 h post knockdown cells were pre-treated with 5 µM MG132 for 3 h and 10 ng/ml HGF for 0.5 h as indicated and protein harvested. TIAM1 was immunoprecipitated under denaturing conditions with a TIAM1 antibody, and immunoprecipitates probed for K48-linked ubiquitin using a K48-linkage specific antibody. (G and H) MDCKII cells were transfected with 2 different siRNAs (#1 and #2) or a non-targeting control siRNA (Ctrl). At 96 h post knockdown cells were pre-treated (3 h) with 5 µM MG132 and 10 ng/ml HGF for 0.5 h. Protein was then harvested and TIAM1 was immunoprecipitated under denaturing conditions with a TIAM1 antibody, and immunoprecipitates probed for K48-linked ubiquitin using a K48-linkage specific antibody. (I and J) MDCKII cells were transfected with HUWE1 siRNA #2 or non-targeting siRNA (Ctrl) as indicated. Cycloheximide (CHX, 50 µg/ml) was added to cells 96 h post transfection where indicated and cells were treated with HGF for the indicated times (I) or left untreated (J). Western blot analysis was performed to analyse amounts of TIAM1 and HUWE1. In C, D and F-J β-ACTIN was used as loading control.

Figure S4, related to Figure 4. HUWE1 mediated TIAM1 degradation controls HGF-induced motility and invasion. (A) MDCKII cells were transfected with siRNA targeting TIAM1 or HUWE1 alone or in combination. At 96 h post transfection cells were stimulated with 20 ng/ml HGF for 18 h and resultant cell scattering was monitored by phase contrast microscopy. Representative images are shown. Scale bar = 50 µm. (B) Western blot analysis of HUWE1 and TIAM1 expression of cells used in Figure 4A and S4A. (C) Western blot analysis of HUWE1 and TIAM1 expression of cells used in Figure 4C-4G. β-ACTIN was used as a loading control in (B) and (C). (D) MDCKII and H1299 cells were transfected with non-targeting siRNA (Ctrl), or siRNA targeting TIAM1 or HUWE1 alone or in combination and seeded in a modified Boyden chamber (where inserts had been coated with 100 µl layer of 500 µg/ml collagen) to assay for invasion in the presence (HGF) of 10 ng/ml HGF. After 5 days, invading cells were stained with Crystal Violet. Panels show representative images from one of at least three independent experiments. Scale bar = 150 µm. (E-F) Crystal Violet was eluted, absorbance measured at 600 nm and relative invasion determined for (E) MDCKII and (F) H1299

cells by plotting optical density against a standard curve of the appropriate cell type previously prepared and normalising this to total cell number for each condition. Results are mean values ± SE. * signifies $p<0.05$, ** $p<0.001$ 5 and **** $p<0.0001$, ns = not significant (Unpaired t test).

Figure S5, related to Figure 5. TIAM1 is ubiquitylated on lysine residue 595. (A) Composite MS/MS spectrum and fragment ion matrix for MGEMQLSSVTDSK (LeuArgGlyGly) supporting the site of TIAM1 ubiquitylation as K595. The peptide sequence and the absence of the LeuArgGlyGly modification N terminal to K13 are confirmed by a b ion series including b2, b3, & b5. The peptide sequence and localisation of the site of Gly Gly modification as K13 is confirmed by a y/y^{++}ion series including y3, y4, $y2^{++}$, $y9^{++}$,$y10^{++}$, & $y12^{++}$. (B) *In vitro* ubiquitylation assays were performed using E1 activating enzyme (UBE1/Uba1), E2 conjugating enzyme (UBE2D1/UbcH5a), E3 ubiquitin ligase (HUWE1-HECT domain), GST-tagged p53 (positive control), TIAM1 PHnCCEx (tagged with GST-His dual tags), GST-only, ubiquitin (Ub) and ATP. ATP or ubiquitin were omitted from the negative controls. Brackets indicate the ubiquitylated isoforms of TIAM1 and p53. Following the *in vitro* reactions protein samples were immunoblotted for either GST (left panel) or p53 (right panel). (C) MDCKII cells inducibly expressing (Plus Dox) either WT TIAM1 (WT #2) or mutant 4x595R TIAM1 (4x595R #2) were seeded on glass coverslips for 48 h. Cells were then fixed and stained by immunofluorescence for E-CADHERIN, TIAM1-HA and DAPI. Panels show representative images from MDCK WT #2 and MDCK 4x595R #2 clones. Scale bar = 10 µm. (D) MDCKII cells inducibly expressing (Plus Dox) either WT TIAM1 (WT #1) or mutant 4x595R TIAM1 (4x595R #1) were seeded on glass coverslips for 48 h, treated with 10 ng/ml HGF for 0.5 h and fixed. The Duolink protocol was then performed to monitor the extent and co-localisation of RAC1 with TIAM1-HA. White arrows indicate co-localisation at intercellular junctions. Scale bar = 10µm. (E) MDCKII cells inducibly expressing (+ Dox) either WT TIAM1 (WT #1 and #2) or mutant 4x595R TIAM1 (4x595R #1 and #2) were lysed and assayed for RAC activity. (F) MDCKII cells inducibly expressing (+ Dox) either WT TIAM1 (WT #2) or mutant 4x595R TIAM1 (4x595R #2) were treated with 5 µM MG132 for 3 h and either left untreated or treated with 10 ng/ml HGF and protein harvested 0.5 h post stimulation. HA-tagged TIAM1 was immunoprecipitated under denaturing conditions with an HA antibody and probed for K48-linked ubiquitin using a K48-linkage specific antibody. (G) MDCKII cells inducibly expressing (Plus Dox) either exogenous HA-tagged WT TIAM1 or mutant 4x595R TIAM1 were treated for 3 h

with 5 µM MG132 and with 20 ng/ml HGF for 1 h, fixed and the Duolink protocol performed to monitor the extent and localisation of K48-linked ubiquitylated TIAM1. White arrows indicate intercellular junctions. Scale bar = 10 µm. (H) MDCKII cells inducibly expressing (+ Dox) either WT TIAM1 (WT #2) or mutant 4x595R TIAM1 (4x595R #2) were treated with 50 µg/ml cycloheximide (CHX) as indicated and HGF (10 ng/ml) for the indicated times, after which protein was extracted. Lysates were immunoblotted for TIAM1-HA expression. β-ACTIN was used as a loading control in F and H. Results are mean values ± SE. *** signifies $p<0.005$ and **** $p<0.0001$, ns = not significant (Unpaired t test).

Figure S6, related to Figure 6. Ubiquitylation of TIAM1 at lysine residue 595 regulates cell migration and invasion. (A-B) MDCKII cells inducibly expressing (Plus Dox) either TIAM1 WT (WT #1) (A) or TIAM1 4x595R (4x595R #1) (B) were stimulated with 20 ng/ml HGF as indicated (Plus HGF). Resultant cell scattering was monitored by phase contrast microscopy. Scale bar = 50 µm. (C) Representative western blot analysis of WT TIAM1 and 4x595R mutant TIAM1 induction in cells used in Figure 6C and 6D and (A) and (B) above. (D) MDCKII, (E) H1299**,** (F) H358 and (G) H522 cells inducibly expressing (Plus Dox) either TIAM1 WT (WT #1 and WT #2) or TIAM1 4x595R (4x595R #1 and 4x595R #2) were seeded in a modified Boyden chamber coated with collagen and left to invade for 1 day in the presence of 10 ng/ml HGF. Invading cells were fixed in 100% methanol and stained with 0.4% Crystal Violet. Panels show representative images from one of at least three independent experiments. Scale bar = 150 µm. (H-J) Representative western blot analysis of WT TIAM1 and 4x595R mutant TIAM1 induction in cells used in Figure 6F-6H and E-G above.

Figure S7, related to Figure 7. HUWE1 mediated TIAM1 degradation controls lung cancer cell invasion *in vivo*. HUWE1 and TIAM1 protein expression are negatively correlated in stage I and stage II squamous cell lung carcinoma. (A) Green fluorescent H1299 cells injected into the pericardial cavity of a Zebrafish embryo. Engrafted cells were imaged at 4 days post injection (dpi). Dotted line in fourth panel indicates the pericardial cavity of the Zebrafish embryo and white arrows in the fifth panel invading cells. Scale bar = 100 µm. (B) Representative immunohistochemistry images of lung squamous cell carcinomas expressing low or high HUWE1, TIAM1 and C-MET protein. Scale bar = 50 µm. (C) Scatter plot depicting the statistically significant Spearman's positive correlation between HUWE1 and C-MET in corresponding tissue sections. (D) Scatter plot depicting the

statistically significant Spearman’s negative correlation between C-MET and TIAM1 in corresponding tissue sections. (E) Model describing the HGF-induced ubiquitylation and degradation of TIAM1 by HUWE1 predominantly at cell–cell adhesions.

SUPPLEMENTAL EXPERIMENTAL PROCEDURES

Constructs. cDNA encoding full-length mouse TIAM1 (GenBank accession NM_009384) carrying a C-terminal HA tag in pCDNA3 expression vector has previously been described (Mack et al., 2012; Michiels et al., 1997; Woodcock et al., 2009). pET-TIAM1 PHCCEx-His-GST was a kind gift from T. Hakoshima (Nara Institute of Science and Technology) and has been previously described (Terawaki et al., 2010). pRetroXT-TIAM1-HA (puro) was cloned by N. Mack (Mack et al., 2012). HA-EGFP dual tags version of this construct was made by subcloning the EGFP tag fused to TIAM1 in pEFGP-TIAM1-HA into pRetro-XT-TIAM1-HA (puro). pRetro-XT-TIAM1-Halo (puro) was made by insertion of the Halo tag amplified from pFC14A-Halo (Promega). pFastBac-TIAM1-HA-His-CTOPO was cloned by sub-cloning full-length Tiam1 into pFastBac-His-CTOPO expression vector (Invitrogen). pCDNA3.1(+)-myc-HisA, pcDNA3, pCDNA3-HA and pCDNA4-TO were purchased from Invitrogen. pGEX-GST-HUWE1-HECT (GST-ARF-BP1 3760-4374) was provided by W. Gu (Columbia University) and has been previously described (Chen et al., 2005) and pSuperior.si5635(puro) was a kind gift from W. Xiaodong and has been previously described (Zhong et al., 2005).

Antibodies. All antibodies used are detailed below.

Primary Antibody	*Species*	*Supplier*
ACTIN	Mouse	Sigma-Aldrich
CAVEOLIN 1	Mouse	BD Biosciences
E-CADHERIN	Rat	Abcam
ERK	Rabbit	Cell Signaling Technology
RAC	Mouse	BD Biosciences
HA-tag (12CA5)	Mouse	Roche
HA-tag	Rabbit	Abcam
HUWE1	Rabbit	Bethyl Lab.
MEK1/2	Rabbit	Cell signalling
Phospho-ERK (Thr202/Tyr204)	Mouse	Cell Signaling Technology
TIAM1	Rabbit	Bethyl Lab.
TIAM1	Sheep	R&D System
Ubiquitin (P4D1)	Mouse	Enzo Lifesciences
Ubiquitin (K48)	Rabbit	Enzo Lifesciences

Secondary antibodies utilized for immunofluorescence were as follows: IgG peroxidase-conjugated (GE Healthcare); Alexa Fluor 488, 568 and 647 (Molecular Probes).

Cell culture. MDCKII and HEK293T cells were maintained in DMEM (Sigma) with 10% FBS (GIBCO) or 10% tetracycline-free FBS (Autogen Bioclear) and supplemented with the appropriate antibiotics (G418, 1 mg/ml; puromycin, 2 µg/ml; blasticidin 1 µg/ml, all from Sigma). The H1299, H358, H522 and H596 lung carcinoma cell lines were all maintained in RPMI (Sigma) with 4mM Glutamine and tetracycline-free 10% FBS. Antibiotic selection was withheld during the experiments.

Generation of cell lines. Retroviral transduction was performed as previously described (Woodcock et al., 2009) and transfections were performed with TransIT-LT1 (Mirus) according to the Manufacturer's instructions followed by appropriate antibiotic selection. For inducible expression, cells were treated with doxycycline (100 ng/ml for confluent cells, 50 ng/ml for sub confluent cells) for at least one day before experimental analysis.

Transient siRNA silencing. Transient silencing of HUWE1 or TIAM1 was achieved by transfection of siRNA oligos from Eurofins MWG operon using RNAiMAX (Invitrogen) following the manufacturer's instructions. Cells were subjected to 'reverse' (cells in suspension) transfection on day 1 and processed and analysed 48-96 h after transfection as indicated in each individual experiment.

Three different siRNA oligos were used to target HUWE1:

Huwe1#1 5'-GAGUUUGGAGUUUGUGAAGUU-3'

Huwe1 #2 5'- UGCCGCAAUCCAGACAUAU-3'

Huwe1 #3 5-AAUUGCUAUGUCUCUGGGACA-3'

Three different siRNA oligos were used to target TIAM1:

Tiam1 #1 5'-GAGGUUGCAGAUCUGAGCA-3'

Tiam1 #2 5'-5'-AGAGCGCACCUACGUGAAA-3'

Tiam1 #3 5'-GCUUGAGACCUGUGUCUUA-3'

In all of the reported assays, the targeting oligos yielded comparable results and for each experiment a non-targeting oligo control was used (Dharmacon – non-targeting siRNA).

Biochemical fractionation. Fractionation was carried out according to a previously described protocol (Holden and Horton, 2009). Briefly cells treated as indicated were permeabilised on ice using digitonin lysis buffer (50mM Tris-HCl pH 7.5, 150mM NaCl, 1mM EDTA and 0.01% digitonin) for 5

min. After a brief centrifugation the cytoplasmic fraction was removed. The insoluble pellet was resuspended in NP40 lysis buffer (150mM NaCl, 50mM HEPES pH 7.4 and 1% NP40) and incubated on ice for a further 30 min. After centrifugation the membrane fraction was removed. Both buffers contained a protease inhibitor cocktail (Sigma), phosphatase inhibitor cocktails 1 and 2 (Sigma) and 5mM sodium vanadate (Sigma). Western blotting was then performed.

Immunoprecipitation. Protein G beads were pre-blocked with an equal volume of 5%BSA/PBS to make a 50% Protein G slurry and lysates pre-cleared using 50% Protein G beads. Cells were treated as indicated in figure legends and lysed in immunoprecipitation lysis buffer (50mM Tris-HCl pH 7.5, 150mM NaCl, 1% (v/v) Triton-X-100, 10% (v/v) glycerol, 2mM EDTA, 25mM NaF and 2mM NaH_2PO4) containing protease and phosphatase inhibitor cocktails (Sigma). Lysates were then incubated with beads conjugated to the appropriate antibody for 3 h at 4ºC with gentle mixing. Beads were then washed 3 times and eluted with 30 µl of 2X SDS sample buffer. Western blotting was then performed.

***In vivo* ubiquitylation experiments.** Detection of protein ubiquitylation in cultured cells was performed using the method described previously (Choo and Zhang, 2009). Briefly either control cells (to detect endogenous TIAM1 ubiquitylation) or cells expressing HA-tagged TIAM1 WT or TIAM1 4x595R (to detect exogenous TIAM1 ubiquitylation) were washed in ice-cold PBS, lysed in 2% SDS denaturing lysis buffer and boiled for 10 min. DNA was then sheared by sonication and samples diluted in dilution buffer (Choo and Zhang, 2009). Samples were mixed for 30 min at 4ºC and spun at 20000 g for 30 min. Protein concentrations were then measured and lysates incubated overnight at 4ºC with beads pre-coated in the antibody of choice (indicated in the individual figure legend). Post incubation beads were spun down at 5000 g for 5 min and washed twice with wash buffer. The remaining resin was then boiled with 2X SDS loading buffer and samples loaded onto an SDS-PAGE gel for immunoblotting.

***In vitro* ubiquitylation experiments.** *In vitro* ubiquitylation assay was performed as previously described (Choo and Zhang, 2009) with some modifications. Briefly, the ubiquitylation reaction was reconstituted using 10 ng of E1 activating enzyme (UBE1/Uba1) (Ubiquigent), 100 ng of E2 conjugating enzyme (UBE2D1/UbcH5a) (Ubiquigent), 400 ng of E3 ubiquitin ligase (HUWE1 HECT

only), 20 ng of GST-tagged p53 (Millipore), 20 ng of PHnCCEX-TIAM1 tagged with GST-His or GST-only, 5 µg of bovine ubiquitin (Sigma-Aldrich), 3 mM ATP in a reaction buffer containing 50 mM Tris, 5 mM $MgCl_2$ and 2 mM DTT at pH7.5. HUWE1 HECT only, PHnCCEX-TIAM1 and GST only were expressed in Rosetta bacteria and purified by GST-pull down as described below. The reaction was incubated at 30°C for 2 hours and subjected to western blot analysis.

GST-pull down. Bacterial cultures expressing HUWE1 HECT domain, PHnCCEX-TIAM1 or GST-only were centrifuged, bacterial pellets washed in PBS and re-pelleted. The pellets were then lysed and sonicated in PBS-based GST lysis buffer containing 0.5 M NaCl, 1 mM EDTA and complete protease inhibitor cocktail (Roche). Lysates were incubated with Glutathione Sepharose beads (GE healthcare) for 2 h and beads washed in GST lysis buffer. Protein was eluted with 10 mM Glutathione (Sigma-Aldrich), 40 mM Tris-HCl pH 8.0 and dialysed overnight into buffer A containing 50 mM Tris-HCl pH 8.0, 0.5 mM $MgCl_2$ and 1 mM β-mercaptoethanol using a Dialysis cassette (Thermo-scientific).

Mass spectrometry analysis. TIAM1 was immunoprecipitated from HEK293T cells as detailed above and after SDS-PAGE the gel was stained using Coomasie (SimplyBlueSafeStain, Invitrogen). The putative bands containing TIAM1 were excised, de-stained and tryptically digested. Gel sections were de-stained by addition of 1 ml of 200 mM ammonium bicarbonate in 40% (v/v) acetonitrile for 20 min at 37°C (3 changes in total). Gel pieces were then dehydrated by addition of 1 ml acetonitrile for 10 min followed by air drying for 20 min. Proteins were digested by adding 100 µl of 40 mM ammonium bicarbonate, 9% (v/v) acetonitrile containing 100 ng of sequencing grade trypsin (Sigma-Aldrich), gel bands rehydrated and 200 µl of 40 mM ammonium bicarbonate, 9% (v/v) acetonitrile added and incubated with the proteins for 16 h at 37ºC. Resulting peptides were dried and resuspended in water containing 0.1% formic acid. Nano flow liquid chromatography was performed using a nano Acquity Ultra High pressure Liquid Chromatography (LC) system. Sample was loaded onto a trap column (Waters Symmetry C18, 180 µm ID, 20 mm 5 µm particles) at 7 µl/min in 1% acetonitrile, 0.1% formic acid for 5 min. Peptides were separated with a linear gradient of acetonitrile 0.1% formic acid from 1-40% over 40 min at a flow rate of 400 nl/min at 65°C (Waters BEH 130 C18 75 µm, ID 250 mm 1.7 µm particles). The LTQ-Orbitrap XL mass spectrometer was used in a data dependent mode. This consisted of a 60,000 resolution FTMS scan followed by up to 6 (most intense

multiply charged precursors between m/z 400-2000) CID MS/MS (at a normalised collision energy of 35%) events performed in parallel in the linear ion trap. Dynamic exclusion was set to 20 s to reduce oversampling of the same precursor. Data was database searched using mascot 2.0 utilising custom variable K modifications of GG and LRGG in addition to conventional variable modifications of M132 oxidation and S,T,Y phosphorylation. Any potential sites of GG or LRGG modification were manually validated. Similarly *in vitro* reactions were carried out as described above and after SDS-PAGE the gel was stained using Coomasie (SimplyBlueSafeStain, Invitrogen). The putative bands containing ubiquitylated TIAM1 were excised, de-stained and tryptically digested and LC-MS/MS performed as above to identify PHnCCEx TIAM1 domain ubiquitylation sites.

RT-PCR. Total RNA was extracted from MDCKII cells either treated with HGF or expressing the inducible HUWE1 knock-down system using RNeasy® Plus Mini kit (Qiagen) following the supplier's protocol. Cells were lysed in 350 µl of Buffer RLT Plus provided in the kit and homogenised by passing through a 1 ml syringe and 0.5 mm needle 6 times. Subsequent steps were carried out according to the protocol with the following modification: total RNA was eluted in 25 µl of RNase-free water provided. The concentration of total RNA extracted was determined using a nanodrop® spectrometer (Thermo Scientific). cDNA was synthesised from 1 µg of the total RNA using oligo dT_{12-18} primer (Invitrogen) and First-Strand cDNA Synthesis kit (Invitrogen), following the supplier's protocol. The cDNA was then subjected to quantitative real-time PCR (qRT-PCR) using an ABI 7900 system (Applied Biosystems). Relative quantitation was performed using the ΔΔCT method.

Halo pulse chase analysis. Briefly MDCKII cells expressing inducible Halo-tagged TIAM1 were seeded at a density of $2x10^3$ cells on a 35 mm glass bottom dish. The following day, cells were treated with 1 µg/ml of Dox to induce expression of the Halo-tagged TIAM1 protein for 24 h before being serum starved for 18 h. Cells were then labelled with 50 nM of HaloTag® TMR Ligands (Promega, Cat. # G8252) in DMEM for 10 min, washed 3 times and Leibovitz medium (Invitrogen) in the presence or absence of 10 ng/ml HGF was added. During the duration of the HGF (or control) treatment 50 µM of blocking reagent Succinimidyl Ester O4 (Promega) was also added. Cells were visualized by live-imaging on an Olympus IX71 microscope. Z-stacks of 0.2 µm per section for a total height of 10 µm were taken for each cell, every 5 min for a total of 1 h. ImageJ was used to quantify Halo-TIAM1 signal at the cell–cell adhesions by manually selecting the regions and then analysed

using the ‘Intensity’ function. Average intensity values of Halo-TIAM1signals for different time points were normalized to 0 min which was set as 1. At least 30 cells per experiment were quantified from 3 independent experiments.

Duolink PLA®. This technique was performed using the Duolink II Red Starter Kit (Sigma) and its basis can be found in (Soderberg et al., 2006). A video summarizing the steps of this technique can be also found online (www.olink.com/products-services/duolink/how-useduolink). Briefly, cells were seeded at low density on glass coverslips and left to attach for 24 h. Cells were then fixed with 3.7 % formaldehyde in PBS and permeabilised for 10 min in TBS (25 mM Tris-HCl [pH 8.0], 150 mM NaCl, 0.1 % Tween-20) containing 0.5 % Triton X-100. Samples were incubated with 3% BSA for 1 h at 37ºC in a humidity chamber and then overnight at 4ºC with an anti-HA mouse and rabbit polyclonal antibody against K48 linked Ub or alternatively a polyclonal rabbit TIAM1 antibody and mouse monoclonal HUWE1 antibody. Cells were also stained with a rat monoclonal E-CADHERIN antibody to mark cell–cell adhesions. Slides were then incubated for 1 h at 37ºC with a mix of the MINUS (anti-mouse) and PLUS (anti-rabbit) PLA probes. Hybridized probes were ligated using the Ligation-Ligase solution for 30 min at 37ºC and then amplified utilizing the Amplification-Polymerase solution for 100 min at 37ºC. Slides were finally mounted using Duolink II Mounting Medium with DAPI and imaged using the Zeiss Axiovert 200M enclosed in a full environmental chamber (Solent Scientific).

Transwell migration and invasion experiments. MDCKII cells or H1299, H358, H522 or H596 lung cancer cells transfected with non-targeting TIAM1, HUWE1 or a combination of TIAM1/HUWE1 siRNAs or overexpressing WT or 4x595R TIAM1 were seeded onto 8 mm transwell migration chambers (Corning, #3422) whose membranes had been pre-coated with either 500 µg/ml (long term invasion assays) or 50 µg/ml (24 h invasion assays) Collagen type I derived from Rat tail (BD Biosciences, #354429). Cells were incubated with serum-free DMEM (MDCKII cell line) or RPMI (lung cancer cell lines) or DMEM (MDCKII) or RPMI (lung cancer cell lines) with 20ng/ml HGF in 10% FBS, added to the lower chamber. Media, including HGF, was replaced every 2 days for the long term 120 h invasion assay. In addition transfection of TIAM1/HUWE1 in MDCKII and H1299 cells was repeated 72 h after the original transfection. MDCKII cells were left to invade for 5 days and H1299 4 days. Cells on the upper part of the membrane were scraped off using a cotton swab and the migrated cells

were fixed in 100% methanol and stained with 0.4% Crystal Violet in 10% ethanol. The experiment was performed in triplicates for all conditions described. From every transwell at least 10 images were taken under a phase-contrast microscope at x 20 magnification. Crystal Violet was then eluted with 33% (v/v) acetic acid in distilled H_2O and 100 µl transferred to a 96-well plate. Absorbance was read at 600 nM and relative invasion determined by plotting optical density against a standard curve of MDCKII, H1299, H358 or H522 or H596 cells previously prepared and normalizing for cell numbers.

Zebrafish xenograft experiments. Zebrafish xenograft experiments were performed as has been previously described (Chapman et al., 2014). Briefly, adult Zebrafish (*Danio rerio*) were maintained at The University of Manchester Biological Services Unit according to National Home Office regulations under the Animals (Scientific Procedures) Act 1986. *Casper* strain (*roy*$^{-/-}$, *nacre*$^{-/-}$) Zebrafish were used as a transparent model to facilitate imaging. H1299 cells were stained using 20 μM Cell Trace™ CFSE Cell Proliferation Kit (Life Technologies) for 15 min at 37°C, followed by a 30 min wash in RPMI medium. Cells were resuspended on ice at 1.3x10^{7}/ml in PBS with 0.5% polyvinylpyrrolidone K60 solution (PVP, Sigma). 48 hours post fertilisation (hpf) embryos were anaesthetised with 0.1mg/ml MS222 (Sigma) and approximately 500 cells were injected into the pericardial cavity using a micropipette and pump (World Precision Instruments). Engrafted embryos were sorted to remove uninjected embryos and maintained at 34°C for a further 3 days. 4 days post-injection (dpi) engrafted Zebrafish were mounted in 1.5% low melting agarose (LMP, Flowgen Biosciences). Tumours were imaged using a Leica TCS SP5 AOBS upright confocal (Leica Microsystems) using a 20 x 0.50 Plan Fluotar dipping objective and 1.3 x confocal zoom. Captured z stacks were processed using Volocity software (Perkin Elmer, Cambridge, UK). All experiments consist of a minimum of three independent repeats. Relative Invasion Index (RII) is defined as the average number of cells invaded outside the pericardial cavity at 4dpi normalized to the average number in a control group.

Immunohistochemical analysis. Scoring was assessed using semi quantitative scoring (H-scores) of the nucleus, membrane and cytoplasm. H-scores were derived from a semi quantitative assessment of both staining intensity (scale 0–3) and the percentage of positive cells (0–100%), which, when multiplied, generated a score ranging from 0 to 900 for all three proteins. TMA immunohistochemistry was evaluated independently by two investigators (D.N. and L.V) and where

discordance (i.e. different scores given by different investigators) was found cases were re-evaluated and a consensus reached. Ethical approval for these studies was conferred under the MCRC Biobank Research Tissue Bank Ethics (ref: 07/H1003/161+5) from NRES Committee North West - Greater Manchester South Ethical.

SUPPLEMENTAL REFERENCES

Chapman, A., Fernandez del Ama, L., Ferguson, J., Kamarashev, J., Wellbrock, C., and Hurlstone, A. (2014). Heterogeneous tumor subpopulations cooperate to drive invasion. Cell Rep *8*, 688-695.

Chen, D., Kon, N., Li, M., Zhang, W., Qin, J., and Gu, W. (2005). ARF-BP1/Mule is a critical mediator of the ARF tumor suppressor. Cell *121*, 1071-1083.

Choo, Y.S., and Zhang, Z. (2009). Detection of protein ubiquitination. J Vis Exp.

Holden, P., and Horton, W.A. (2009). Crude subcellular fractionation of cultured mammalian cell lines. BMC Res Notes *2*, 243.

Mack, N.A., Porter, A.P., Whalley, H.J., Schwarz, J.P., Jones, R.C., Khaja, A.S., Bjartell, A., Anderson, K.I., and Malliri, A. (2012). beta2-syntrophin and Par-3 promote an apicobasal Rac activity gradient at cell-cell junctions by differentially regulating Tiam1 activity. Nat Cell Biol *14*, 1169-1180.

Michiels, F., Stam, J.C., Hordijk, P.L., van der Kammen, R.A., Ruuls-Van Stalle, L., Feltkamp, C.A., and Collard, J.G. (1997). Regulated membrane localization of Tiam1, mediated by the NH2-terminal pleckstrin homology domain, is required for Rac-dependent membrane ruffling and C-Jun NH2-terminal kinase activation. J Cell Biol *137*, 387-398.

Soderberg, O., Gullberg, M., Jarvius, M., Ridderstrale, K., Leuchowius, K.J., Jarvius, J., Wester, K., Hydbring, P., Bahram, F., Larsson, L.G.*, et al.* (2006). Direct observation of individual endogenous protein complexes in situ by proximity ligation. Nat Methods *3*, 995-1000.

Terawaki, S., Kitano, K., Mori, T., Zhai, Y., Higuchi, Y., Itoh, N., Watanabe, T., Kaibuchi, K., and Hakoshima, T. (2010). The PHCCEx domain of Tiam1/2 is a novel protein- and membrane-binding module. EMBO J *29*, 236-250.

Woodcock, S.A., Rooney, C., Liontos, M., Connolly, Y., Zoumpourlis, V., Whetton, A.D., Gorgoulis, V.G., and Malliri, A. (2009). SRC-induced disassembly of adherens junctions requires localized phosphorylation and degradation of the rac activator tiam1. Mol Cell *33*, 639-653.

Zhong, Q., Gao, W., Du, F., and Wang, X. (2005). Mule/ARF-BP1, a BH3-only E3 ubiquitin ligase, catalyzes the polyubiquitination of Mcl-1 and regulates apoptosis. Cell *121*, 1085-1095.
